# Transcriptome Profiling Reveals the Response of Seed Germination of *Peganum harmala* to Drought Stress

**DOI:** 10.3390/plants13121649

**Published:** 2024-06-14

**Authors:** Zhen Zhang, Hongyan Su, Qingen Li, Mengfei Li

**Affiliations:** 1 State Key Laboratory of Aridland Crop Science, Gansu Agricultural University, Lanzhou 730070, China; zhzh@gsau.edu.cn (Z.Z.); shy922322@163.com (H.S.); 2 Station of Forestry and Grassland, Alxa Right Banner 737300, China; ayqlqe@163.com

**Keywords:** *Peganum harmala*, drought stress, seed germination, transcriptome profiling

## Abstract

*Peganum harmala* L. is a perennial herbaceous plant that plays critical roles in protecting the ecological environment in arid, semi-arid, and desert areas. Although the seed germination characteristics of *P. harmala* in response to environmental factors (i.e., drought, temperature, and salt) have been investigated, the response mechanism of seed germination to drought conditions has not yet been revealed. In this study, the changes in the physiological characteristics and transcriptional profiles in seed germination were examined under different polyethylene glycol (PEG) concentrations (0–25%). The results show that the seed germination rate was significantly inhibited with an increase in the PEG concentration. Totals of 3726 and 10,481 differentially expressed genes (DEGs) were, respectively, generated at 5% and 25% PEG vs. the control (C), with 1642 co-expressed DEGs, such as drought stress (15), stress response (175), and primary metabolism (261). The relative expression levels (RELs) of the key genes regulating seed germination in response to drought stress were in accordance with the physiological changes. These findings will pave the way to increase the seed germination rate of *P. harmala* in drought conditions.

## 1. Introduction

*Peganum harmala* L. (family Zygophyllaceae) is a perennial herbaceous plant mainly distributed in arid, semi-arid, and desert areas in China (e.g., Xinjiang, Ningxia, and the Inner Mongolia province), and it plays critical roles in preventing land degradation [1]. Furthermore, it has been widely used to treat diabetes, hypertension, nervous system disorders, etc.; these uses largely rely on its bioactive compounds, including alkaloids, flavonoids, and anthraquinones [2]. 

As is known, drought significantly limits plant biomass, crop yield, and agricultural cultivation [3]. Plants can respond to drought stress through a series of physiological, biochemical, and nutritional networks [4]. Extensive studies have demonstrated that drought stress inhibits plant growth while improving the activity of antioxidant enzymes (e.g., SOD, CAT, and APX) by inducing the expression levels of related genes and stress-inducible genes encoding the enzymes involved in ABA biosynthesis [5,6]. Previous studies on *Peganum multisectum* Bobr (the same genus with *P. harmala*) have found that drought stress significantly increased the SOD and CAT activities as well as the soluble sugar, MDA, and Pro contents [7]. 

Seed germination is the first step toward continuous growth and development, especially under adverse conditions [8,9]. Generally, drought stress significantly affects the seed germination parameters (e.g., cumulative percent germination, maximum seed germination, and seed germination rate) [10]. A series of physiological and biochemical changes occur during seed germination in response to drought stress [11]. Early studies showed that the seed germination rate of *P. harmala* significantly decreased under drought stress, high temperature (>30 °C), and high salt concentrations (>100 mmol/L NaCl) [12,13]; in contrast, it increased under low salt concentrations (<50 mmol/L NaCl), low concentrations of a Ni^2+^, Cu^2+^, and Co^2+^ solution, and fluctuating temperatures (e.g., 30 °C/15 °C) [13,14]. 

To date, although the seed germination characteristics of *P. harmala* in response to different stress factors (i.e., drought, temperature, and salt) have been investigated [12,13,14], the regulatory mechanisms in the seed germination stage have not yet been revealed. In this study, the physiological characteristics and transcriptional profiles of *P. harmala* at the seed germination stage were examined under PEG 6000-induced drought stress.

## 2. Results

### 2.1. Germination Characteristics and Physiological Indices under PEG Treatments

**Figure 1:**
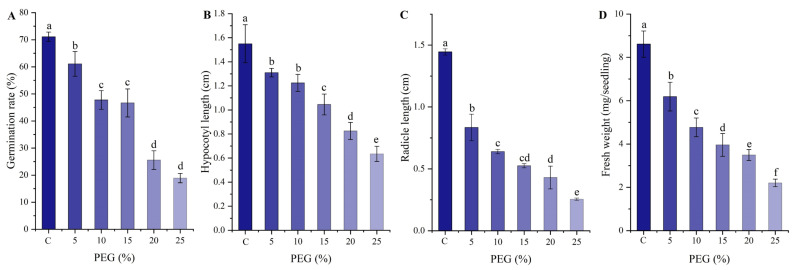
Seed germination characteristics of *Peganum harmala* under PEG treatments. Images (**A**–**D**) show the germination rate, hypocotyl length, radicle length, and fresh weight, respectively. Different letters indicate a significant difference at the *p* < 0.05 level.

The seed germination was significantly affected by the PEG-induced drought stress (Figure 1). The germination characteristics (i.e., seed germination rate, length of hypocotyl and radicle, and fresh weight) significantly decreased with an increase in the PEG concentration (Figure 1A–D). Meanwhile, the PEG treatments significantly adjusted the activities of antioxidant enzymes (i.e., SOD, POD, CAT, and APX) and the contents of osmolytes (i.e., soluble sugar, protein, MDA, and Pro) (Figures S1 and S2). 

### 2.2. Transcriptome Profiles under PEG Treatments

#### 2.2.1. Transcriptomic Analysis

After filtered sequences at C, 5%, and 25% PEG, totals of 67.48, 78.91, and 80.30 million high-quality reads were assembled, and 53.99, 63.17, and 63.97 million unique reads with 2.08, 2.43, and 2.49 million multiple reads were obtained, respectively (Table 1; Figure S3).

A total of 81,625 unigenes were annotated on the KEGG (37,725), KOG (24,809), Nr (41,636), and Swissprot databases (29,846) (Figure S4). Specifically, 46.22% transcripts were enriched in 139 biochemical pathways on the KEGG (Figure S5); 30.39% transcripts encoded the proteins on the KOG (Figure S6); the top 10 species (i.e., *Pistacia vera*, *Citrus clementine*, *Carica papaya*, *Coffea arabica*, *Citrus sinensis*, *Vitis vinifera*, *Prunus dulcis*, *Actinidia chinensis*, *Theobroma cacao*, and *Rhodamnia argentea*) were located on the NR (Figure S7); 36.56% transcripts were annotated on the Swissprot; and three ontologies were partitioned on the GO (Figure S8).

#### 2.2.2. Excavation of DEGs

**Figure 2:**
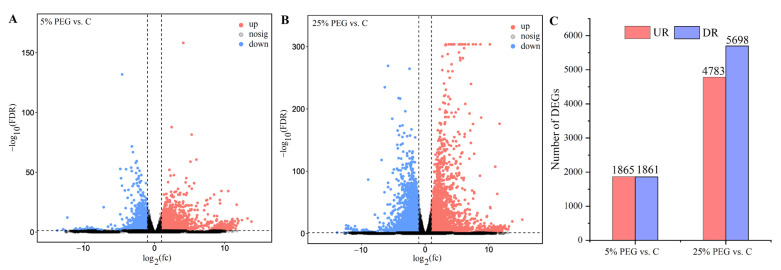
Volcano plot of unigenes and number of DEGs under 5% and 25% PEG vs. C. Images (**A**,**B**) show the volcano plots; image (**C**) shows the number of DEGs.

Totals of 3726 (1865 up-regulated (UR), 1861 down-regulated (DR)) and 10,481 (4783 UR, 5698 DR) DEGs were excavated from the 5% and 25% PEG vs. C, respectively (Figure 2), based on the comprehensive analysis (Figures S9–S11). 

#### 2.2.3. Identification of DEGs

**Figure 3:**
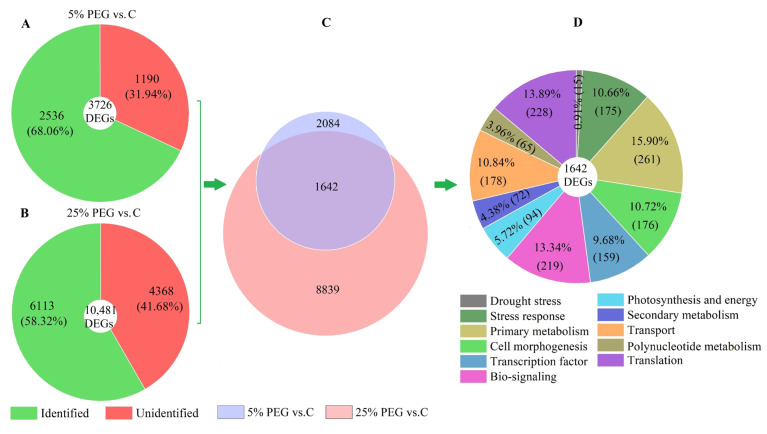
Classification of DEGs under PEG treatments. Images (**A**,**B**) show the identified and unidentified DEGs; image (**C**) shows their Venn diagram; and image (**D**) shows the classification of the co-expressed DEGs.

After function retrieval from the databases, 2536 and 6113 genes were identified from the above 3726 and 10,481 DEGs, respectively, with 1642 genes co-expressed at C, 5%, and 25% PEG (Figure 3A–C). Furthermore, the 1642 genes were classified into 11 categories, such as drought stress (15 genes), cell morphogenesis (176 genes), and transcription factor (159 genes) (Figure 3D). 

### 2.3. Specific Classification of DEGs and Expression Levels Validated via qRT-PCR

#### 2.3.1. DEGs Associated with Drought Stress 

**Figure 4:**
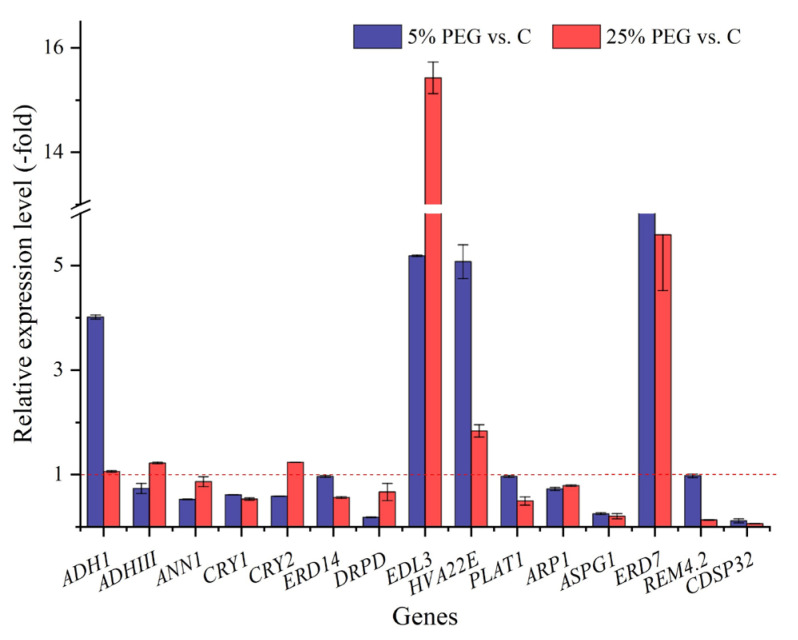
The RELs of drought stress genes in *P. harmala* (mean ± SD, n = 3). Abbreviations: *ADH1*, alcohol dehydrogenase class P; *ADHIII*, alcohol dehydrogenase class 3; *ANN1*, annexin D1; *CRY1*, cryptochrome 1; *CRY2*, cryptochrome 2; *ERD14*, dehydrin ERD14; *DRPD*, desiccation-related protein PCC27-45; *EDL3*, EID1-like F-box protein 3; *HVA22E*, HVA22-like protein e; *PLAT1*, PLAT domain-containing protein 1; *ARP1*, probable RNA-binding protein ARP1; *ASPG1*, protein ASPARTIC PROTEASE IN GUARD CELL 1; *ERD7*, protein EARLY-RESPONSIVE TO DEHYDRATION 7; *REM4.2*, remorin 4.2; *CDSP32*, thioredoxin-like protein CDSP32; vs., versus.

In this study, 15 genes were directly associated with drought stress (Table S1). The RELs of 4 genes showed 4.02- (*ADH1*) to 11.53-fold (*ERD7*) UR and those of 11 genes showed 0.12- (*CDSP32*) to 0.98-fold (*REM4.2*) DR under 5% PEG vs. C, while those of 6 genes showed 1.01- (*ADH1*) to 15.43-fold (*EDL3*) UR and those of 9 genes showed 0.06- (*CDSP32*) to 0.87-fold (*ANN1*) DR under 25% PEG vs. C (Figure 4). The RELs of most genes (e.g., *ADH1*, *ANN1*, and *CRY1*) were consistent with their RPKM values (Table S1).

#### 2.3.2. DEGs Associated with Antioxidant Enzyme Activities

**Figure 5:**
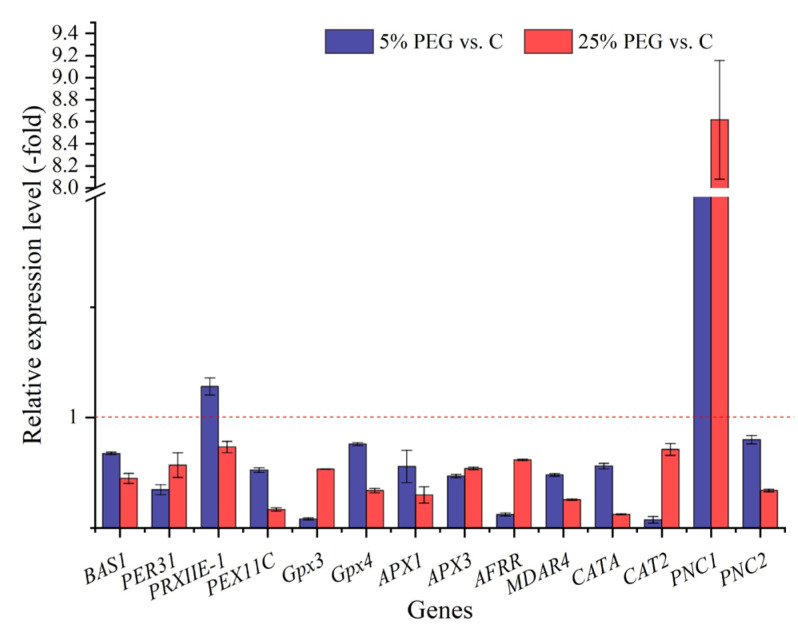
The RELs of antioxidant enzymes genes in *P. harmala* under 5% and 25% PEG vs. C (mean ± SD, n = 3). Abbreviations: *BAS1*, 2-Cys peroxiredoxin BAS1; *PER31*, peroxidase 31; *PRXIIE-1*, peroxiredoxin-2E-1; *PEX11C*, peroxisomal membrane protein 11C; *Gpx3*, glutathione peroxidase 3; *Gpx4*, phospholipid hydroperoxide glutathione peroxidase; *APX1*, L-ascorbate peroxidase; *APX3*, L-ascorbate peroxidase 3; *AFRR*, monodehydroascorbate reductase; *MDAR4*, monodehydroascorbate reductase 4; *CATA*, catalase; *CAT2*, catalase isozyme 2; *PNC1*, cationic peroxidase 1; *PNC2*, cationic peroxidase 2; vs., versus.

A total of 20 antioxidant enzymes genes were excavated from the 175 stress response genes (Tables S2 and S3). The RELs of 2 genes showed 1.28- (*PRXIIE-1*) and 6.07-fold (*PNC1*) UR, and those of 12 genes showed 0.07- (*CAT2*) to 0.80-fold (*PNC2*) DR under 5% PEG vs. C, while those of 1 gene showed 8.62-fold (*PNC1*) UR, and those of 13 genes showed 0.12- (*CATA*) to 0.73-fold (*PRXIIE-1*) DR under 25% PEG vs. C (Figure 5). The RELs of most genes (e.g., *BAS1*, *PER31*, and *PEX11C*) were consistent with their RPKM values (Table S2).

#### 2.3.3. DEGs Associated with Osmolytes

**Figure 6:**
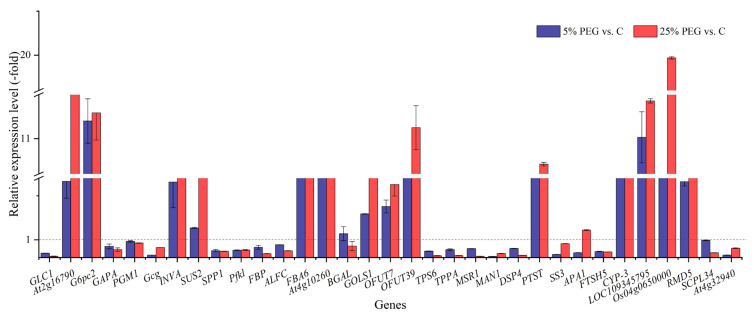
The RELs of osmolytes genes in *P. harmala* under 5% and 25% PEG vs. C (mean ± SD, n = 3). Abbreviations: *GLC1*, glucan endo-1,3-beta-glucosidase; *At5g58090*, glucan endo-1,3-beta-glucosidase 6; *G6pc2*, glucose-6-phosphatase 2; *GAPA*, glyceraldehyde-3-phosphate dehydrogenase A; *PGM1*, phosphoglucomutase; *Gcg*, pro-glucagon; *INVA*, alkaline/neutral invertase A; *SUS2*, sucrose synthase 2; *SPP1*, sucrose-phosphatase 1; *Pfkl*, ATP-dependent 6-phosphofructokinase; *FBP*, fructose-1,6-bisphosphatase; *ALFC*, fructose-bisphosphate aldolase; *FBA6*, fructose-bisphosphate aldolase 6; *At4g10260*, probable fructokinase-5; *BGAL*, beta-galactosidase; *GOLS1*, galactinol synthase 1; *OFUT7*, O-fucosyltransferase 7; *OFUT39*, O-fucosyltransferase 39; *TPS6*, alpha,alpha-trehalose-phosphate synthase (UDP-forming) 6; *TPPA*, trehalose-phosphate phosphatase A; *MSR1*, protein MANNAN SYNTHESIS-RELATED 1; *MAN1*, mannan endo-1,4-beta-mannosidase 1; *DSP4*, phosphoglucan phosphatase DSP4; *PTST*, protein PTST homolog 3; *SS3*, soluble starch synthase 3; *APA1*, aspartic proteinase A1; *FTSH5*, ATP-dependent zinc metalloprotease FTSH 5; *CYP-3*, cysteine proteinase 3; *LOC109345795*, gamma conglutin 1; *Os04g0650000*, oryzain alpha chain; *RMD5*, protein RMD5 homolog; *SCPL34*, serine carboxypeptidase-like 34; *At4g32940*, vacuolar-processing enzyme gamma-isozyme; vs., versus.

A total of 68 osmolytes genes were excavated from the 261 primary metabolism genes (Tables S4 and S5). The RELs of 33 genes were selected for validation, with those of 15 genes showing 1.35- (*BGAL*) and 12.01-fold (*G6pc2*) UR and those of 18 genes showing 0.06- (*MAN1*) to 0.98-fold (*SCPL34*) DR under 5% PEG vs. C, while those of 15 genes showed 1.56- (*APA1*) to 19.85-fold (*Os04g0650000*) UR, and those of 18 genes showed 0.07- (*MSR1*) to 0.82-fold (*PGM1*) DR under 25% PEG vs. C (Figure 6). The RELs of most genes (e.g., *GLC1*, *At2g16790*, and *G6pc2*) were consistent with their RPKM values (Table S4).

#### 2.3.4. DEGs Associated with Seed Germination

**Figure 7:**
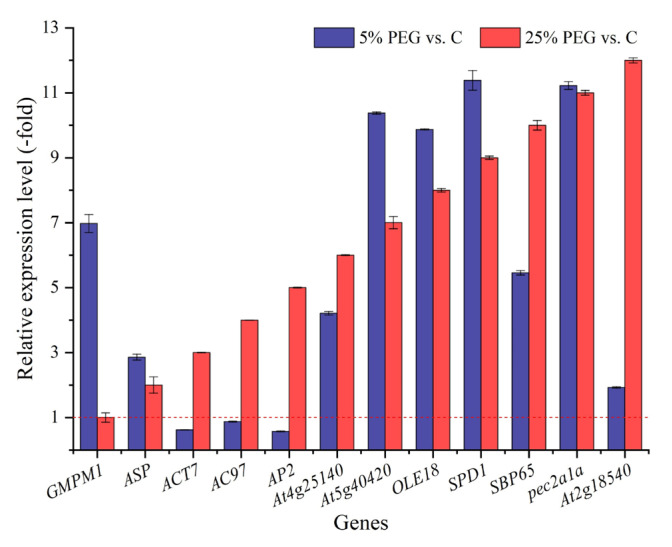
The RELs of genes directly associated with seed germination of *P*. *harmala* under 5% and 25% PEG vs. C (mean ± SD, n = 3). Abbreviations: *GMPM1*, 18 kDa seed maturation protein; *ASP*, 21 kDa seed protein; *ACT7*, actin 7; *AC97*, actin 97; *AP2*, floral homeotic protein APETALA 2; *At4g25140*, oleosin 18.5 kDa; *At5g40420*, oleosin 21.2 kDa; *OLE18*, oleosin Zm-II; *SPD1*, protein SEEDLING PLASTID DEVELOPMENT 1; *SBP65*, seed biotin-containing protein SBP65; *pec2a1a*, Vicilin Car i 2.0101; *At2g18540*, vicilin-like seed storage protein At2g18540; vs., versus.

A total of 12 genes directly associated with seed germination were excavated from the 176 cell morphogenesis genes (Tables S6 and S7). The RELs of 9 genes showed 1.93- (*At2g18540*) and 11.38-fold (*SPD1*) UR and those of 3 genes showed 0.57- (*AP2*) to 0.87-fold (*AC97*) DR under 5% PEG vs. C, while those of 10 genes showed 1.06- (*AP2*) to 17.59-fold (*At4g25140*) UR and those of 2 genes showed 0.60-(*ACT7*) to 0.63-fold (*AC97*) DR under 25% PEG vs. C (Figure 7). The RELs of most genes (e.g., *GMPM1*, *ASP*, and *At4g25140*) were consistent with their RPKM values (Table S6).

#### 2.3.5. TFs Associated with Seed Germination and Stress Response 

**Figure 8:**
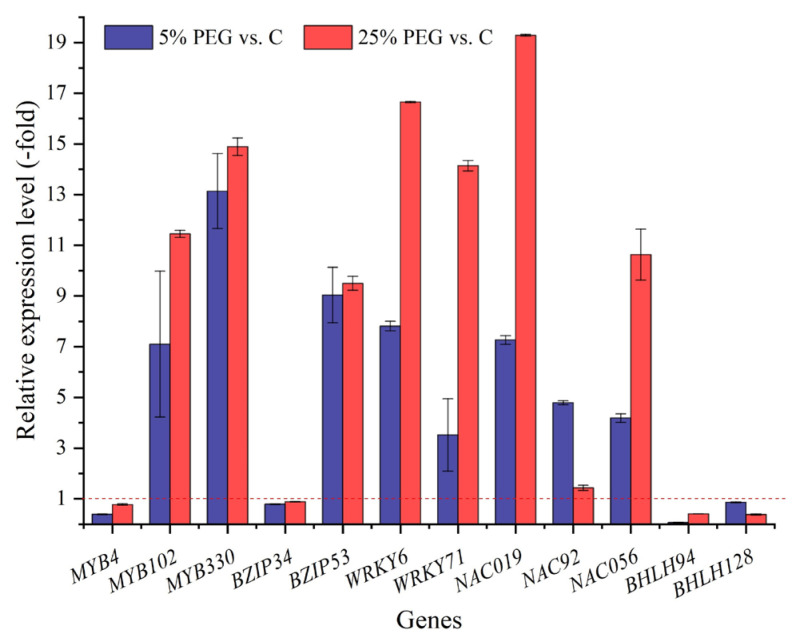
The RELs of TFs directly associated with seed germination and stress response in *P*. *harmala* under 5% and 25% PEG vs. C (mean ± SD, n = 3). Abbreviations: *MYB4*, transcription factor MYB4; *MYB102*, transcription factor MYB102; *MYB330*, Myb-related protein 330; *BZIP34*, basic leucine zipper 34; *BZIP53*, bZIP transcription factor 53; *WRKY6*, WRKY transcription factor 6; *WRKY71*, WRKY transcription factor 71; *NAC019*, NAC domain-containing protein 19; *NAC92*, NAC domain-containing protein 92; *NAC056*, NAC transcription factor 56; *BHLH94*, transcription factor bHLH94; *BHLH128*, transcription factor bHLH128; vs., versus.

A total of 42 TFs directly associated with seed germination and the stress response were excavated from the 159 TFs (Tables S8 and S9). The RELs of 12 genes were selected for validation, with those of 8 genes showing 3.52- (*WRKY71*) to 13.14-fold (*MYB330*) UR and those of 4 genes showing 0.06- (*BHLH94*) to 0.86-fold (*BHLH128*) DR under 5% PEG vs. C, while those of 8 genes showed 1.43- (*NAC92*) to 19.29-fold (*NAC019*) UR and those of 4 genes showed 0.38- (*BHLH128*) to 0.88-fold (*BZIP34*) DR under 25% PEG vs. C (Figure 8). The RELs of most genes (e.g., *MYB4*, *MYB102*, and *MYB330*) were consistent with their RPKM values (Table S8).

#### 2.3.6. DEGs Associated with Hormones

**Figure 9:**
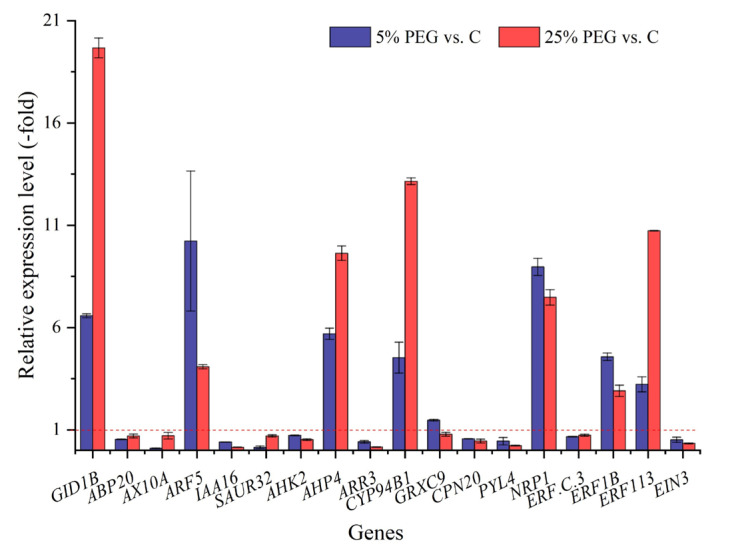
The RELs of genes associated with hormones in *P. harmala* under 5% and 25% PEG vs. C (mean ± SD, n = 3). Abbreviations: *GID1B*, gibberellin receptor GID1B; *ABP20*, auxin-binding protein ABP20; *AX10A*, auxin-induced protein X10A; *ARF5*, auxin response factor 5; *IAA16*, auxin-responsive protein IAA16; *SAUR32*, auxin-responsive protein SAUR32; *AHK2*, histidine kinase 2; *AHP4*, histidine-containing phosphotransfer protein 4; *ARR3*, two-component response regulator ARR3; *CYP94B1*, cytochrome P450 94B1; *GRXC9*, glutaredoxin-C9; *CPN20*, 20 kDa chaperonin; *PYL4*, abscisic acid receptor PYL4; *NRP1*, nodulin-related protein 1; *ERF.C.3*, ethylene-response factor C3; *ERF1B*, ethylene-responsive TF 1B; *ERF113*, ethylene-responsive TF ERF113; *EIN3*, protein ETHYLENE INSENSITIVE 3; vs., versus.

A total of 52 genes directly associated with hormones were excavated from the 219 bio-signaling genes (Tables S10 and S11). The RELs of 18 genes were selected for validation, with those of 8 genes showing 1.47- (*GRXC9*) to 10.23-fold (*ARF5*) UR and those of 10 genes showing 0.10- (*AX10A*) to 0.72-fold (*AHK2*) DR under 5% PEG vs. C, while those of 7 genes showed 2.90- (*ERF1B*) to 19.68-fold (*GID1B*) UR and those of 11 genes showed 0.14- (*IAA16*) to 0.78-fold (*GRXC9*) DR under 25% PEG vs. C (Figure 9). The RELs of most genes (e.g., *GID1B*, *ABP20*, and *AX10A*) were consistent with their RPKM values (Table S10).

#### 2.3.7. Other Functional DEGs 

Moreover, 490 DEGs were identified from the *P. harmala* in response to drought stress. The specific data are shown in Figure 3 and Tables S12–S16. 

## 3. Discussion

Plants are frequently exposed to unfavorable environments, restricting their growth and development [15]. Drought seriously inhibits plant growth and development by interrupting various biological processes [16]. In this study, the physiological characteristics and transcriptional profiles of *P. harmala* were significantly affected during seed germination in response to PEG-induced drought stress.

Previous studies on drought have found that plants undergo changes in their phenotypes, antioxidant enzyme activities, and osmolyte contents [17]. For example, the germination rate of *P. harmala* significantly decreased under water limitations, PGE treatments, and dry cold storage [12,18]. The antioxidant enzymes and osmolytes in *Peganum multisectum* Bobr were affected under soil drought stress [7]. The contents of soluble sugar, free amino acids, and Pro significantly increased, while MDA gradually increased in *Seabuckthorn* under drought stress [19]. Furthermore, significant fluctuations of genes involved in protein kinases and TFs have been identified in response to drought stress [20]; for example, the expression levels of *DHN1*, *RCD1*, and *LIPC* were found to be related to the response to water-deficient up-regulation under PEG treatments [9]. 

In this study, the seed germination was significantly inhibited with an increase in the PEG concentrations, and the activities of antioxidant enzymes and contents of osmolytes were significantly altered under different PEG treatments. Meanwhile, 1642 DEGs were observed to be co-expressed in *P. harmala*, with 11 categories classified (e.g., drought stress, stress response, and primary metabolism) (see Figure 3).

Specifically, 15 genes directly participate in drought stress. For example, *ADH1* and *ADHIII* are involved in the response to water deprivation [21]; *ANN1* is differentially regulated under drought conditions [22]; *CRY1* and *CRY2* participate in the response to water deprivation [23]; *ERD7* and *ERD14* participate in the rapid response to dehydration [24]; *DRPD* participates in the response to desiccation and is abundantly expressed in dried leaves [25]; *EDL3* participates in the response to drought stress via the plant hormones [26]; *HVA22E* plays a role in stress tolerance and is differentially regulated by dehydration stress [27]; *PLAT1* acts as a positive regulator in response to abiotic stress [28]; *ARP1* is involved in the response to water deprivation, affecting ABA-regulated seed germination [29]; *ASPG1* is involved in drought avoidance through ABA signaling [30]; *REM4.2* plays a role in various abiotic stresses and participates in the SnRK1-mediated signaling pathway [31]; and *CDSP32* participates in the plastid defense against oxidative damage in response to water deprivation [32]. Based on the results of this study, we believe that these DEGs largely contribute to the seed germination under drought stress.

Generally, plants have evolved an antioxidant enzyme system to protect them from damage [33]. Here, 20 genes were found to be directly associated with antioxidant enzyme activities. For example, *BAS1*, *PER31*, and *PRXIIE-1* play critical roles in cell protection against oxidative stress [34]; *PEX11C* is involved in peroxisomal proliferation [35]; *Gpx3* participates in catalyzing the reduction of peroxides [36]; *Gpx4* participates in preventing membrane lipid peroxidation [37]; *APX1* and *APX3* play key roles in hydrogen peroxide removal [38]; *AFRR* and *MDAR4* are involved in the detoxification of H_2_O_2_ [39,40]; *CATA* and *CAT2* protect cells from peroxide toxicity [41]; and *PNC1* and *PNC2* are involved in the response to the removal of H_2_O_2_ [42]. Based on the results of this study, these DEGs participate in regulating the antioxidant enzyme activities under drought stress.

For the 68 genes directly associated with osmolytes, 52 genes participate in soluble sugar metabolism. For example, *At2g16790* is involved in D-gluconate degradation [43]; *G6pc2* is involved in gluconeogenesis and carbohydrate biosynthesis [44]; *PGM1* is involved in glucose metabolism [45]; *Gcg* plays a key role in glucose metabolism and homeostasis [46]; *INVA* cleaves sucrose into glucose and fructose [47]; *SUS2* catalyzes sucrose synthesis and cleavage [48]; *SPP1* is involved in sucrose biosynthesis [49]; *Pfkl* participates in the biosynthesis of D-glyceraldehyde 3-phosphate and glycerone phosphate [50]; *FBP* participates in the biosynthesis of fructose-1,6-bisphosphate and fructose-6-phosphate [51]. In this study, these DEGs must participate in regulating the soluble sugar and protein contents under drought stress. 

Regarding the 12 genes directly associated with seed germination, briefly, *ACT7* and *AC97* are involved in seed germination and play an important role in cell division, organelle movement, and extension growth [52]; *AP2* is required during seed development [53]; *SBP65* plays roles in determining the seed germination capacity [54]; *pec2a1a* and *At2g18540* act as seed storage proteins and play critical roles in seed development [55]. In this study, these DEGs must play dominant roles in seed germination under drought stress.

TFs play significant roles in regulating plant growth and development as well as signal networks [56]. Based on the results of this study, 42 TFs participate in seed germination and stress response. For example, MYB TFs participate in the drought, salt, and cold stress responses [57]; BZIP TFs (*BZIP34* and *BZIP53*) act as transcriptional activators of seed development [58]; WRKY TFs (*WRKY6* and *WRKY71*) are involved in the control of processes related to senescence and pathogen defense [59]; NAC TFs (*NAC019*, *NAC92,* and *NAC056*) are involved in the response to water deprivation and gene regulation during seed germination [60,61]; and BHLH TFs (*BHLH94* and *BHLH143*) are involved in the response to abiotic stresses [62]. The results of this study indicate that these IFs may play critical roles in regulating the seed germination of *P. harmala*. 

As is known, plant hormones play critical roles in the adaptation to various stresses [9]. Based on the results of this study, 52 genes participate in the hormone response; for example, *GID1B* acts as a GA receptor and is required for GA signaling, which controls seed germination [63]; *AHK2* regulates several developmental processes, including seed germination, cell division, and shoot promotion [64]; and *PYL4* is involved in ABA-mediated responses, such as germination inhibition [65]. These DEGs must regulate the fluctuation of hormones to control the seed germination of *P. harmala*.

**Figure 10:**
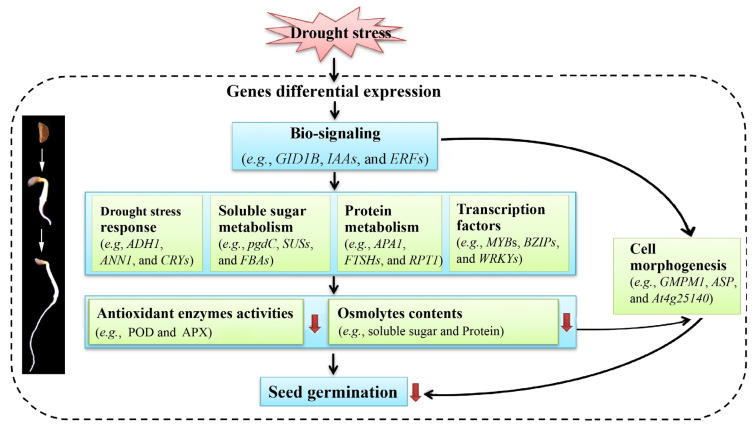
An outlined model of seed germination of *P. harmala* in response to drought stress. Abbreviations: *ADH1*, alcohol dehydrogenase class P; *ANN1*, annexin D1; *CRYs*, cryptochromes; *pgdC*, 6-phosphogluconate dehydrogenase, decarboxylating 1; *SUSs*, sucrose synthase; *FBAs*, fructose-bisphosphate aldolases; *APA1*, aspartic proteinase A1; *FTSHs*, ATP-dependent zinc metalloprotease FTSHs; *RPT1*, 26S proteasome regulatory subunit 7; *MYBs*, transcription factor MYBs; *BZIPs*, bZIP transcription factors; *WRKYs*, WRKY transcription factors; *GMPM1*, 18 kDa seed maturation protein; *ASP*, 21 kDa seed protein; *At4g25140*, oleosin 18.5 kDa.

Based on the physiological characteristics and transcriptome profiles, a model of the seed germination of *P. harmala* in response to drought stress is outlined in Figure 10. Briefly, when seeds are exposed to drought stress, stress genes are induced to show differential expressions to switch on bio-signaling. Subsequently, low expression levels of genes participating in drought stress responses, soluble sugar metabolism, protein metabolism, and TFs will result in lower antioxidant enzyme activities and osmolyte contents, leading to membrane injury under drought stress; finally, these changes lead to cell morphogenesis, inhibiting the seed germination of *P. harmala*. 

## 4. Materials and Methods

### 4.1. Plant Materials

*P. harmala* L. seeds were harvested from Alxa Left Banner of China in October 2019, air-dried, stored at 4 °C for two months, and then sown in a 15 cm Petri dish with 5 mL of PEG-6000, including: 0 (C, 0 MPa), 5% (−0.05 MPa), 10% (−0.15 MPa), 15% (−0.30 MPa), 20% (−0.50 MPa), and 25% (−0.77 MPa) [66]. There were 40 independent biological replicates for each treatment, with 30 seeds per dish.

### 4.2. Measurement of Germination Characteristics

After 3 d, the seed germination rate was measured. After 6 d, the lengths of the hypocotyls and radicles as well as the fresh weights of germinated seedlings were measured. Forty independent biological replicates were used for each measurement.

### 4.3. Determination of Antioxidant Enzyme Activities and Osmolyte Contents

The activities of SOD, POD, CAT, and APX were determined using a spectrometer at 560, 470, 240, and 290 nm, respectively [67,68,69,70]. The osmolyte contents of soluble sugar, protein, MDA, and Pro were determined with the methods of phenolsulfuric acid, coomassie brilliant blue colorimetric, thiobarbituric acid, and sulfosalicylic acid–acid ninhydrin, respectively [71,72,73,74]. Three biological replicates with 40 independent biological replicates were used for each determination.

### 4.4. Transcriptomic Analysis

High-quality RNA samples of the C, 5, and 25% PEG-6000 seedlings were used to construct the cDNA library, and the biosynthesis of second-strand cDNA and purification of cDNA fragments were performed according to previous protocols [75]. Their transcriptome profiles were analyzed using the Illumina HiSeqTM 4000 platform, with the filtration of raw reads, the assembly of clean reads, the normalization of transcripts to RPKM values, the expression analysis of different treatments, and the annotation of DEGs [76,77,78,79]. 

### 4.5. qRT-PCR Validation of Selected Genes

Primer-BLAST in the NCBI was used to design the primer sequence (Table S17). The cDNA synthesis, PCR amplification, and melting curve analysis were performed according to the manufacturers of the kits. The RELs of genes were calculated using the 2^−^^ΔΔ*Ct*^ method, with *actin* used as a reference control [80]. 

### 4.6. Statistical Analysis

Significant differences at the *p* < 0.05 level were analyzed using ANOVA and Duncan’s post hoc test in SPSS 22.0.

## 5. Conclusions

The above observations reveal that the seed germination of *P. harmala* was inhibited under drought stress, with significant physiological changes in its antioxidant enzyme activities and osmolyte contents. The differential expressions of related genes play critical roles in regulating seed germination under drought stress. To identify the specific roles of genes related to drought resistance, additional studies are required. 

## Figures and Tables

**Table 1:** Summary of sequencing data under different PEG treatments (mean ± SD, n = 3).

	C	5% PEG	25% PEG
**Filtered data**			
Data of reads number (million)	67.48 ± 7.63	78.91 ± 6.68	80.30 ± 11.65
Data of reads number×read length (million)	10,122 ± 1144	11,836 ± 1002	12,044 ± 1747
Q20 (%)	96.32 ± 0.23	96.49 ± 0.10	96.69 ± 0.17
Q30 (%)	90.92 ± 0.43	91.17 ± 0.13	91.56 ± 0.32
**Mapped data**			
Data of unique mapped reads (million)	53.99 ± 6.06	63.17 ± 5.37	63.97 ± 9.35
Data of multiple mapped reads (million)	2.08 ± 0.84	2.43 ± 0.84	2.49 ± 0.83
Mapping ratio (%)	83.84 ± 0.18	83.68 ± 0.05	83.32 ± 0.10

## Data Availability

The datasets are publicly available at NCBI (https://www.ncbi.nlm.nih.gov/bioproject/PRJNA782232, accessed on 31 December 2022) and Sequence Read Archive (SRA) accession: C (SRR17012455, SRR17012461, and SRR17012462), 5% PEG (SRR17012448, SRR17012459, and SRR17012460), and 25% PEG (SRR17012456, SRR17012457, and SRR17012458).

## Supplementary Materials

The following supporting information can be downloaded at: https://www.mdpi.com/article/10.3390/plants13121649/s1. Figure S1: Changes in the antioxidant enzymes activities of *P. harmala* at the seed germination stage under different PEG treatments; Figure S2: Changes in the osmolyte contents of *P. harmala* at the seed germination stage under different PEG treatments; Figure S3: Length distribution of assembled unigenes; Figure S4: Basic annotation of all unigenes of *P. harmala*; Figure S5: Annotation of unigenes in KEGG database; Figure S6: Distribution of unigenes with KOG functional classification; Figure S7: Annotation of unigenes in NR database; Figure S8: Annotation of unigenes in GO database; Figure S9: Principal component analysis of C, 5% and 25% PEG; Figure S10: Pearson correlation analysis of C, 5%, and 25% PEG; Figure S11: Heat map of the DEGs under 5% and 25% PEG vs. C. Table S1: Genes associated with drought stress; Table S2: Genes associated with antioxidant enzymes; Table S3: Genes associated with other stress responses; Table S4: Genes associated with soluble sugar and protein metabolism; Table S5: Genes associated with other primary metabolism; Table S6: Genes associated with cell morphogenesis for seed germination; Table S7: Genes associated with other cell morphogenesis; Table S8: TFs associated with stress response and seed germination; Table S9: Other TFs at 5% and 25% PEG vs. C; Table S10: Genes associated with hormone response; Table S11: Genes associated with other bio-signaling; Table S12: Genes associated with photosynthesis and energy; Table S13: Genes associated with secondary metabolism; Table S14: Genes associated with transport; Table S15: Genes associated with polynucleotide metabolism; Table S16: Genes associated with translation; Table S17: Sequences of primers used for qRT-PCR validation.
