# Supplementary material for: Transcriptome Profiling Reveals the Response of Seed Germination of Peganum harmala to Drought Stress
Source: Plants (Basel). 2024 Jun 14;13(12):1649. doi: 10.3390/plants13121649 (PMC11207268; doi:10.3390/plants13121649)
Supplement: Supplementary file 1 [file plants-13-01649-s001.zip › plants-3042795-supplementary.pdf]

Supplementary Materials

Figure supplementary legends

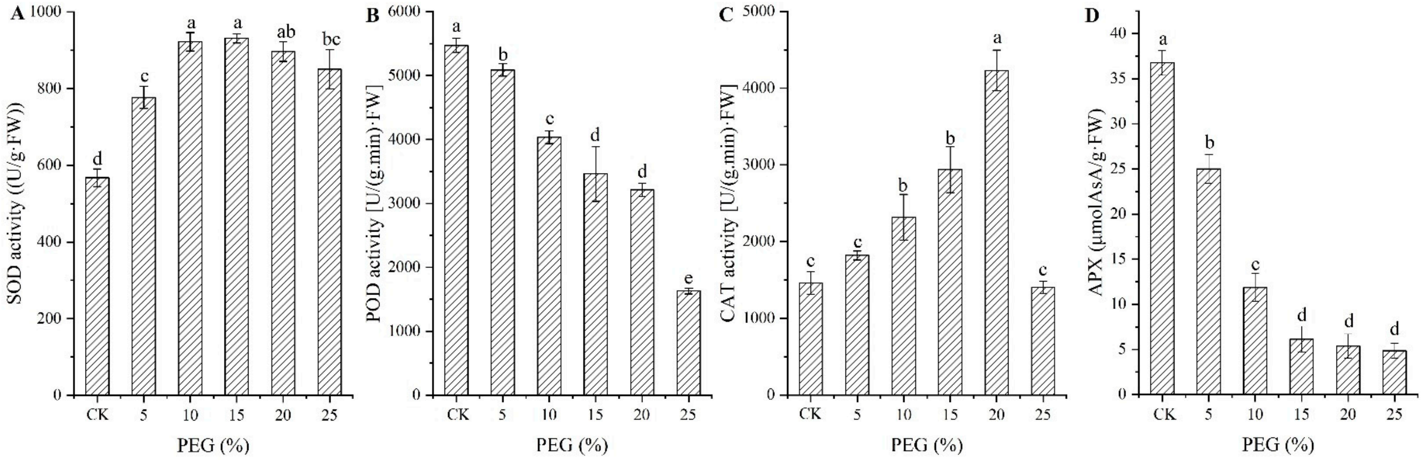

Figure S1. Changes in the activities of the four antioxidant enzymes SOD (A), POD (B), CAT (C), and APX (D) in *P. harmala* at seed germination stage under different PEG treatments. Different letters represent a significant difference ($p < 0.05$) among different PEG treatments.

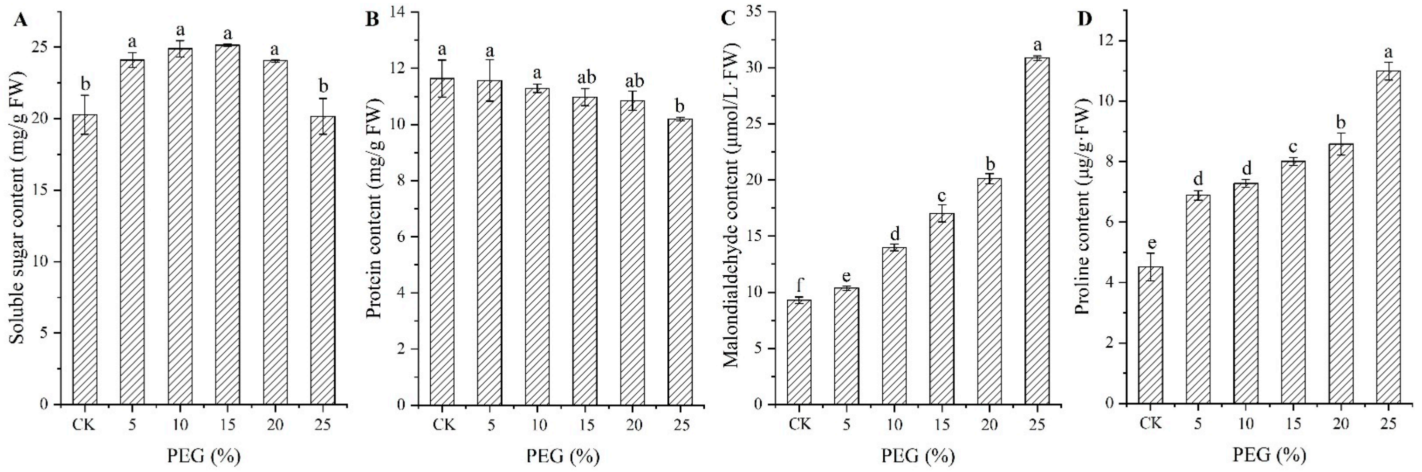

Figure S2. Changes in the contents of soluble sugar (A), protein (B), Malondialdehyde (C), and proline (D) in *P. harmala* at seed germination stage under different PEG treatments. Different letters represent a significant difference ($p < 0.05$) among different PEG treatments.

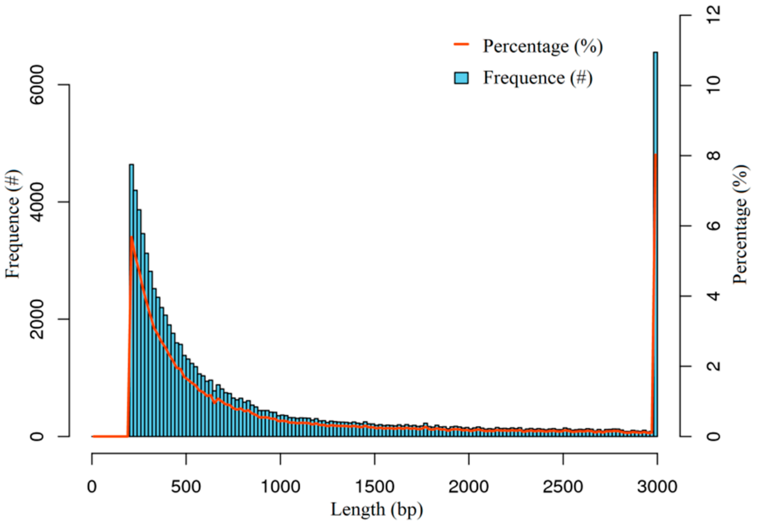

Figure S3. Length distribution of assembled unigenes.

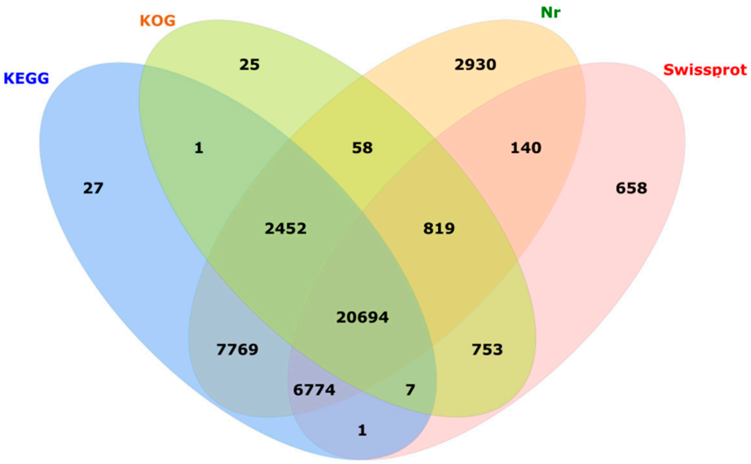

Figure S4. Basic annotation for all unigenes in *P. harmala* on KEGG, KOG, Nr, and Swissprot databases.

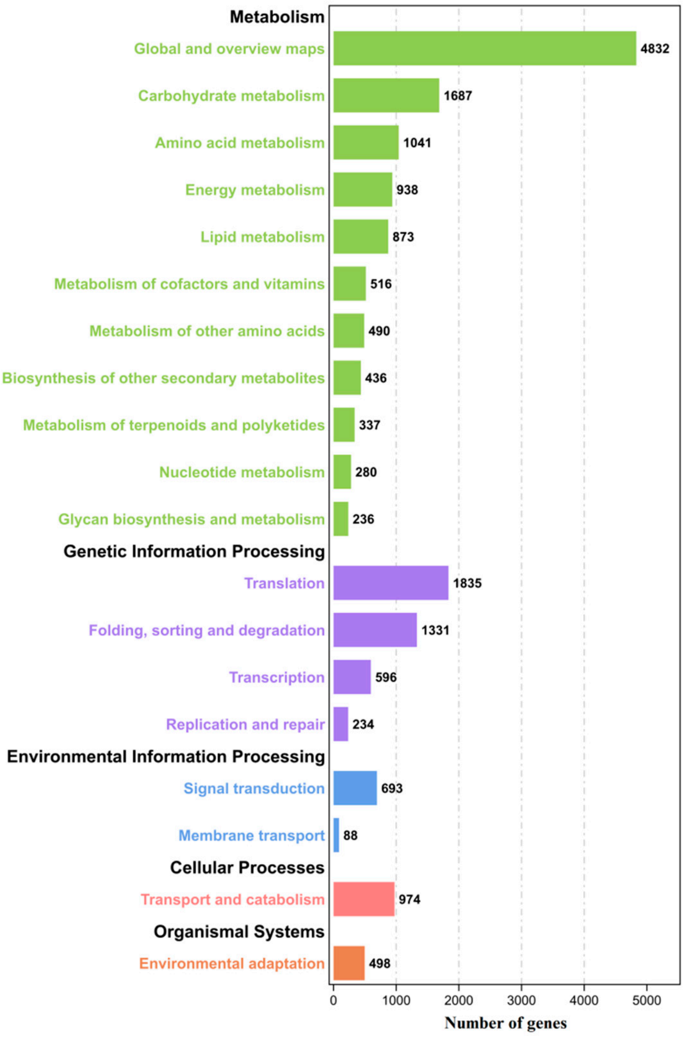

Figure S5. Annotation of unigenes on KEGG database and biochemical pathways.

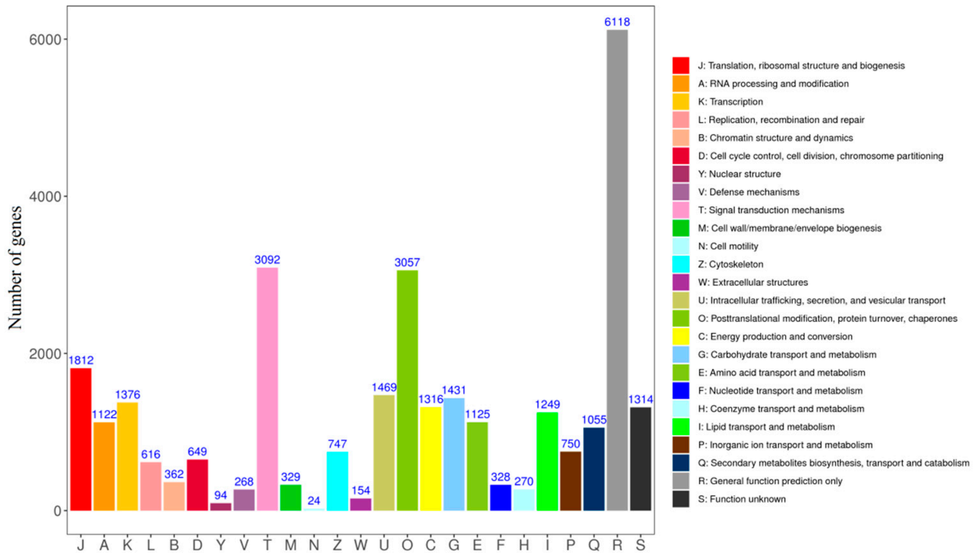

Figure S6. Distribution of unigenes in the transcriptome with KOG functional classification.

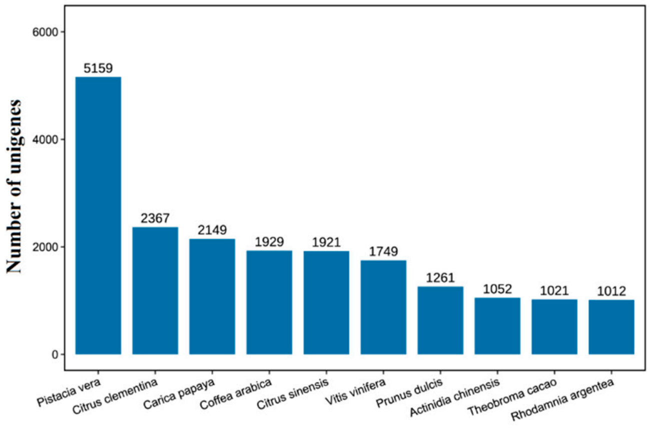

Figure S7. Annotation of unigenes on NR database and distribution of the top 10 species.

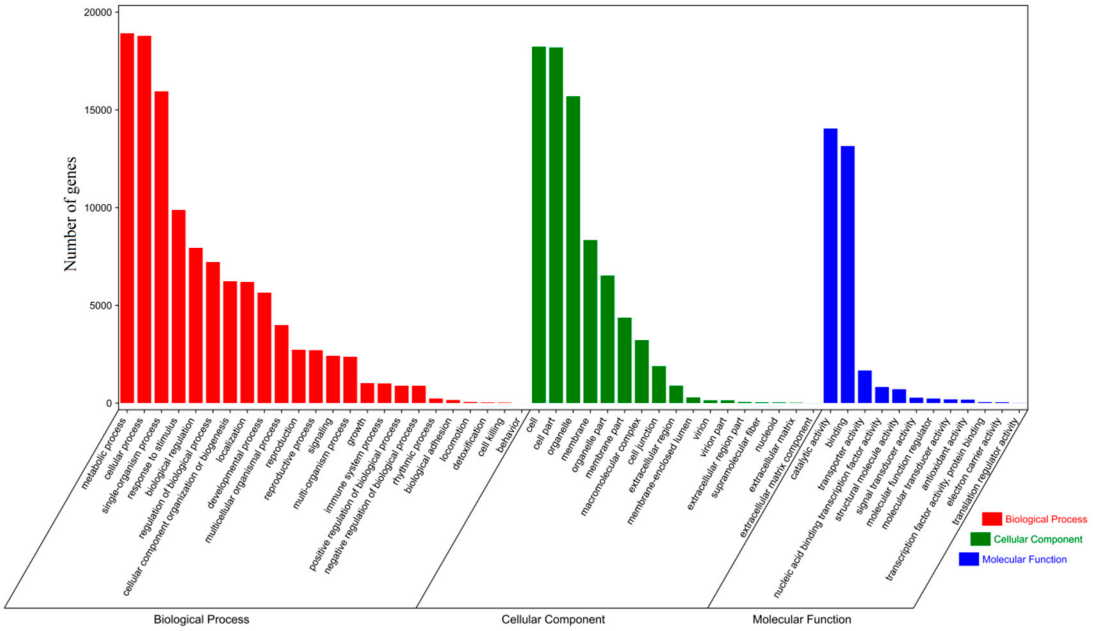

Figure S8. Annotation of unigenes on GO database and biological classification.

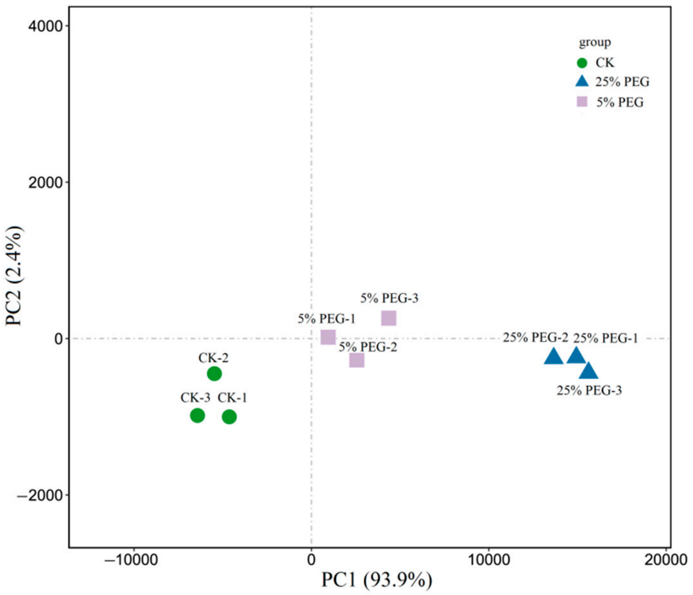

Figure S9. Principal component analysis (PCA) of CK, 5 and 25% PEG.

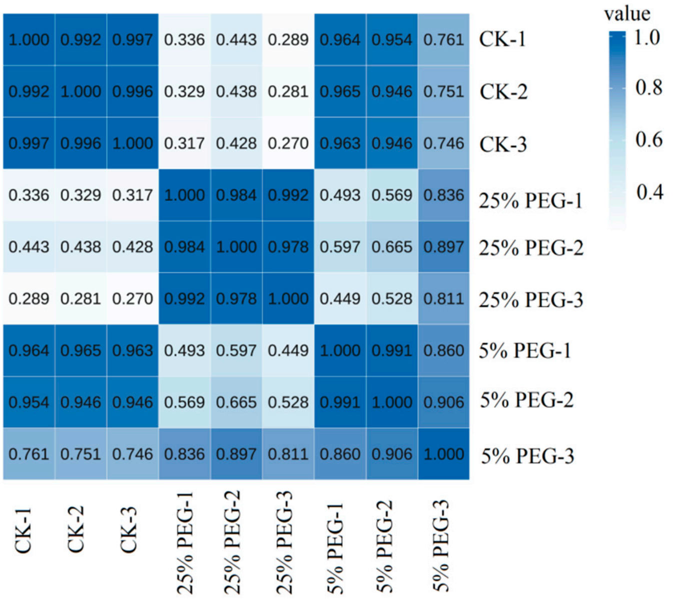

Figure S10. Pearson correlation analysis of CK, 5 and 25% PEG.

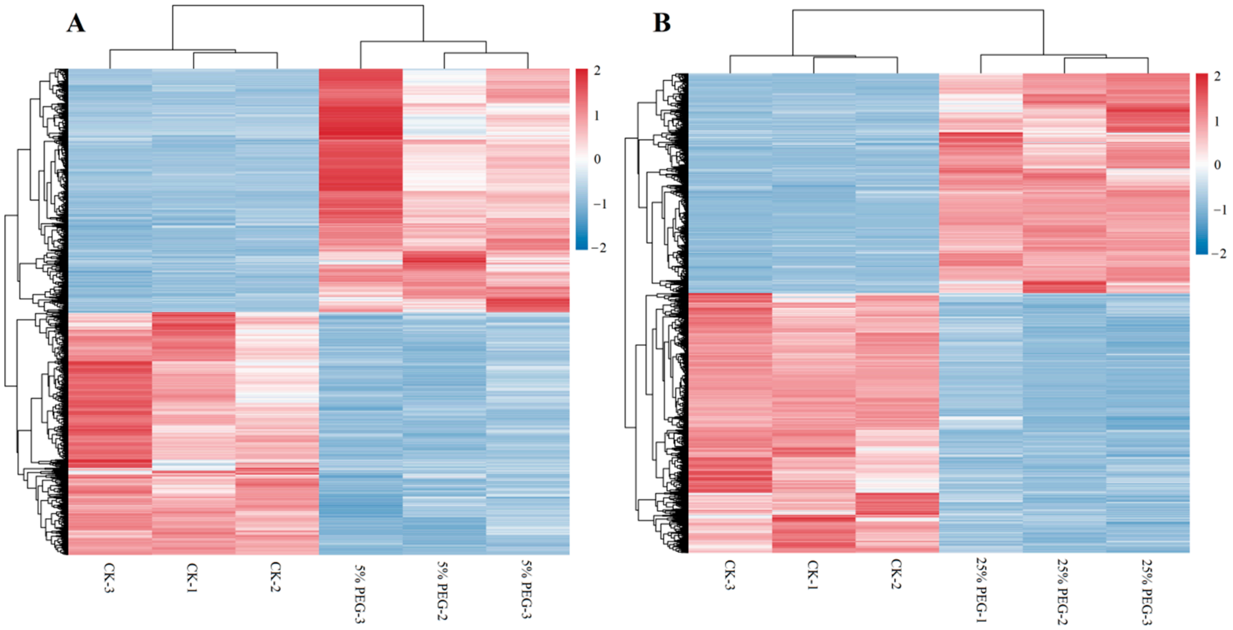

Figure S11. Cluster heat map of the DEGs at 5% PEG vs. CK (A) and 25% PEG vs. CK (B).

Table supplementary legends

Table S1. Fifteen genes directly associated with drought stress under 5% and 25% PEG vs. CK.

Gene name	SwissProt ID	Protein name	log_2FC (5% PEG vs. CK)	log_2FC (25% PEG vs. CK)
ADH1	P06525	Alcohol dehydrogenase class-P	4.19	5.42
ADHIII	A2XAZ3	Alcohol dehydrogenase class-3	− 2.22	− 1.65
ANN1	Q9SYT0	Annexin D1	− 2.56	− 2.16
CRY1	Q43125	Cryptochrome-1	− 2.43	− 2.37
CRY2	Q96524	Cryptochrome-2	− 4.34	− 8.40
ERD14	P42763	Dehydrin ERD14	− 2.43	− 2.32
DRPD	P22241	Desiccation-related protein PCC27-45	− 2.47	− 2.03
EDL3	Q93ZT5	EID1-like F-box protein 3	4.51	5.56
HVA22E	Q9FED2	HVA22-like protein e	1.72	1.75
PLAT1	O65660	PLAT domain-containing protein 1	− 1.90	− 2.03
ARP1	Q9M1S3	Probable RNA-binding protein ARP1	− 2.67	− 2.16
ASPG1	Q9LS40	Protein ASPARTIC PROTEASE IN GUARD CELL 1	− 2.33	− 2.03
ERD7	O48832	Protein EARLY-RESPONSIVE TO DEHYDRATION 7	2.48	4.59
REM4.2	P93758	Remorin 4.2	1.80	1.75
CDSP32	Q9SGS4	Thioredoxin-like protein CDSP32	− 3.06	− 2.60

Table S2. Twenty genes directly associated with antioxidant enzyme activities under 5% and 25% PEG vs. CK.

Gene name	SwissProt ID	Protein name	log_2FC (5% PEG vs. CK)	log_2FC (25% PEG vs. CK)
**POD (12)**				
REHY	Q6E2Z6	1-Cys peroxiredoxin	4.36	6.07
BAS1	Q96291	2-Cys peroxiredoxin BAS1	− 2.18	− 2.23
PER12	Q96520	Peroxidase 12	1.10	2.06
PER31	Q9LHA7	Peroxidase 31	− 1.07	− 2.55
PER42	Q9SB81	Peroxidase 42	− 2.10	− 2.13
PRDX1	Q06830	Peroxiredoxin-1	9.70	9.17
PRXIIE-1	Q69TY4	Peroxiredoxin-2E-1	− 2.23	− 2.14
PEX11C	Q9LQ73	Peroxisomal membrane protein 11C	− 2.09	− 2.18
PEX13	Q9SRR0	Peroxisomal membrane protein 13	1.32	1.68
Gpx3	P46412	Glutathione peroxidase 3	− 3.09	− 3.30
Gpx4	O70325	Phospholipid hydroperoxide glutathione peroxidase	− 1.72	− 1.69
GPX6	O48646	Probable phospholipid hydroperoxide glutathione peroxidase 6	− 1.17	− 0.21
**APX (4)**				
APX1	P48534	L-ascorbate peroxidase	− 2.56	− 2.02
APX3	Q42564	L-ascorbate peroxidase 3	− 2.56	− 1.93
AFRR	Q43497	Monodehydroascorbate reductase	− 2.48	− 2.85
MDAR4	Q9LK94	Monodehydroascorbate reductase 4	− 2.32	− 2.03
**CAT (4)**				
CATA	O24339	Catalase	− 2.05	− 2.37
CAT2	P30567	Catalase isozyme 2	− 2.08	− 1.87
PNC1	P22195	Cationic peroxidase 1	1.50	− 0.04
PNC2	P22196	Cationic peroxidase 2	− 1.31	− 2.74

Table S3. One hundred and fifteen-five genes associated with other stress response at 5 and 25% PEG vs. CK.

Gene name	SwissProt ID	Protein name	log_2FC (5% PEG vs. CK)	log_2FC (25% PEG vs. CK)
At5g59530	Q9LTH8	1-aminocyclopropane-1-carboxylate oxidase homolog 11	5.22	6.77
HSP15.4	O49710	15.4 kDa class V heat shock protein	-1.03	-3.35
HSP17.7	O81822	17.7 kDa class II heat shock protein	5.33	7.10
HSP17.9	P46516	17.9 kDa class II heat shock protein	9.79	11.30
HSP17.9-D	P05477	17.9 kDa class II heat shock protein	1.86	2.13
HSP18.1	Q84Q72	18.1 kDa class I heat shock protein	10.92	12.42
HSP18.2	P27880	18.2 kDa class I heat shock protein	2.19	3.27
HSP23.6	Q96331	23.6 kDa heat shock protein	2.42	2.94
At3g50940	Q147F9	AAA-ATPase At3g50940	2.22	0.35
ACR4	Q8LJW3	ACT domain-containing protein ACR4	-1.87	-2.74
ACR8	Q9LNA5	ACT domain-containing protein ACR8	-1.40	-2.69
ACR11	Q9FZ47	ACT domain-containing protein ACR11	-2.57	-2.65
ALDH2B4	Q9SU63	Aldehyde dehydrogenase family 2 member B4	-2.38	-2.46

BTG-26	Q41247	Aldehyde dehydrogenase family 7 member A1	1.84	3.02
ALDH7B4	Q9SYG7	Aldehyde dehydrogenase family 7 member B4	1.09	2.23
N/A	P23901	Aldose reductase	11.82	13.71
ALD1	Q9ZQI7	Aminotransferase ALD1	4.10	4.38
NPR4	A2CIR5	Ankyrin repeat-containing protein NPR4	1.12	1.41
ANXA2	P07355	Annexin A2	11.46	10.59
ANXA5	P08758	Annexin A5	9.31	8.31
APF2	Q9LNJ3	Aspartyl protease family protein 2	-0.93	-2.47
N/A	P37707	B2 protein	-0.37	-2.38
BAG6	O82345	BAG family molecular chaperone regulator 6	2.04	3.00
BAG7	Q9LVA0	BAG family molecular chaperone regulator 7	-2.22	-1.79
B2M	P61770	Beta-2-microglobulin	10.31	9.54
BPA1	Q9LFD5	Binding partner of ACD11 1	-1.16	-3.31
RD22	Q08298	BURP domain protein RD22	-2.34	-2.09
CATHB3	Q94K85	Cathepsin B-like protease 3	-2.24	-2.37
CBSCBSPB3	Q9LF97	CBS domain-containing protein CBSCBSPB3	-2.62	-2.47
CBSX1	O23193	CBS domain-containing protein CBSX1	-2.95	-0.51
CBSX3	Q9LEV3	CBS domain-containing protein CBSX3	-2.09	-2.25
CBSX5	Q84WQ5	CBS domain-containing protein CBSX5	-2.26	-1.76
CLPB1	Q6F2Y7	Chaperone protein ClpB1	4.79	6.42
CLPD	P42762	Chaperone protein ClpD	2.98	4.41
ATJ8	Q9SAG8	Chaperone protein dnaJ 8	-2.09	-2.52
ATJ10	Q8GYX8	Chaperone protein dnaJ 10	1.71	2.98
CEQORH	Q9SV68	Chloroplast envelope quinone oxidoreductase homolog	1.32	2.10
P23-1	Q9FR62	Co-chaperone protein p23-1	1.52	2.47
Cfd	P03953	Complement factor D	-2.34	-2.91
COR413PM1	Q9XIM7	Cold-regulated 413 plasma membrane protein 1	-3.03	-2.24
RD19A	P43296	Cysteine protease RD19A	1.25	1.79
N/A	P25804	Cysteine proteinase 15A	-2.65	-2.13
Os01g0915200	Q5N806	Cysteine proteinase inhibitor 4	5.04	6.07
RD21A	P43297	Cysteine proteinase RD21A	0.42	1.17
CRRSP38	Q9LRJ9	Cysteine-rich repeat secretory protein 38	-1.36	-4.19
CYP76C1	O64636	Cytochrome P450 76C1	4.00	5.72
CYP78A5	Q9LMX7	Cytochrome P450 78A5	1.43	2.10
CYP82A3	O49858	Cytochrome P450 82A3	-0.03	1.49
CYP89A9	Q9SRQ1	Cytochrome P450 89A9	-0.17	0.01
N/A	H2DH18	Cytochrome P450 CYP736A12	3.43	4.17
N/A	H2DH17	Cytochrome P450 CYP749A22	1.10	1.96
At1g52590	Q9SSR1	DCC family protein At1g52590	1.27	2.31
N/A	Q07502	Defensin-like protein	9.18	10.06
PDF2.4	Q9C947	Defensin-like protein 5	7.20	8.22
DGK1	Q39017	Diacylglycerol kinase 1	1.58	2.30
RPP13	Q9M667	Disease resistance protein RPP13	-1.07	-0.68
RPS5	O64973	Disease resistance protein RPS5	-2.12	-1.74
CHN48	P08252	Endochitinase A	1.52	1.89
At1g61340	Q8GX77	F-box protein At1g61340"	1.92	1.95
FLZ10	Q9LYE4	FCS-Like Zinc finger 10	1.78	2.44
N/A	Q9ZR41	Glutaredoxin	-2.57	-2.79
GSTF9	O80852	Glutathione S-transferase F9	1.81	3.67
HSP70	P26413	Heat shock 70 kDa protein	8.50	10.17
HSP70-14	Q9S7C0	Heat shock 70 kDa protein 14	-2.00	-2.66
HSP70	P09189	Heat shock cognate 70 kDa protein	2.32	0.76
HSPA8	A2Q0Z1	Heat shock cognate 71 kDa protein	2.84	2.37
HSC-I	P24629	Heat shock cognate 70 kDa protein 1	9.40	11.27
HSC80	P36181	Heat shock cognate protein 80	-1.93	-1.90
HSF24	P22335	Heat shock factor protein HSF24	1.28	2.21
HSP83A	P51819	Heat shock protein 83	2.97	5.13
HSP90-5	Q9SIF2	Heat shock protein 90-5	-2.02	-2.62
HSP90AA1	P07900	Heat shock protein HSP 90-alpha	8.63	7.02
HSP90AB1	P08238	Heat shock protein HSP 90-beta	9.42	8.93
HSFB2A	Q9SCW4	Heat stress transcription factor B-2a	2.53	2.59
HIPP05	Q9SJL2	Heavy metal-associated isoprenylated plant protein 5	-2.54	-2.07
HHP1	Q93ZH9	Heptahelical transmembrane protein 1	1.18	2.14
HHP4	Q9SZG0	Heptahelical transmembrane protein 4	1.91	3.00
RCI2A	Q9ZNQ7	Hydrophobic protein RCI2A	1.25	2.64
RCI2B	Q9ZNS6|RC	Hydrophobic protein RCI2B	-3.23	-2.23
IAN9	F4HT21	Immune-associated nucleotide-binding protein 9	-2.62	-3.23

JAC1	Q9C9Q4	J domain-containing protein required for chloroplast accumulation response 1	-2.43	-2.64
At5g17165	F4KFM8	Late embryogenesis abundant protein At5g17165	-2.58	-1.95
ECP63	Q9SKP0	Late embryogenesis abundant protein ECP63	3.79	4.75
LEA5	Q39644	Late embryogenesis abundant protein Lea5	-2.39	-2.53
LEA5-D	P46522	Late embryogenesis abundant protein Lea5-D	4.09	6.22
LEA14-A	P46518	Late embryogenesis abundant protein Lea14-A	3.05	4.23
FLBR	Q9SPB1	Leghemoglobin reductase	-1.89	-2.02
LRR1	Q9FPJ5	Leucine-rich repeat protein 1	-2.99	-3.04
LTI65	Q04980	Low-temperature-induced 65 kDa protein	4.82	6.12
LT101.2	Q9ARD5	Low temperature-induced protein lt101.2	1.12	1.89
LYK4	O64825	LysM domain receptor-like kinase 4	-1.41	-2.45
MIF	Q6DN04	Macrophage migration inhibitory factor	9.42	8.99
At1g73050	Q9SSM2	(R)-mandelonitrile lyase-like	2.33	3.40
N/A	O50001	Major allergen Pru ar 1	-2.98	-2.33
MLP22	Q41020	Major latex protein 22	1.05	1.24
PM19L	Q6L4D2	Membrane protein PM19L	9.55	11.33
MLO2	Q9SXB6	MLO-like protein 2	-1.18	-2.18
MLO6	Q94KB7	MLO-like protein 6	1.55	2.19
NHL10	Q9SJ52	NDR1/HIN1-like protein 10	-4.01	-1.56
ATL24	Q8LBA0	NEP1-interacting protein-like 2	-1.54	-5.95
NUDT8	Q8L7W2	Nudix hydrolase 8	-1.03	-2.86
N/A	Q41350	Osmotin-like protein	-3.21	-3.43
OSM34	P50700	Osmotin-like protein OSM34	-1.77	-0.89
AAE3	Q9SMT7	Oxalate--CoA ligase	1.56	1.69
PTI5	O04681	Pathogenesis-related genes transcriptional activator PTI5	3.47	4.36
PMAT1	Q940Z5	Phenolic glucoside malonyltransferase 1	1.77	2.80
PES2	Q9LW26	Phytyl ester synthase 2	0.24	0.32
PGIP1	Q9M5J9	Polygalacturonase inhibitor 1	-1.75	-2.13
GIP2	P0DO21	Probable aspartic proteinase GIP2	1.03	2.21
At1g59620	Q9LQ54	Probable disease resistance protein At1g59620	-2.09	-1.41
SRO5	Q9FJJ3	Probable inactive poly [ADP-ribose] polymerase SRO5	1.49	1.73
GH3.1	O82333	Probable indole-3-acetic acid-amido synthetase GH3.1	3.64	4.11
CAD	Q9ZRF1	Probable mannitol dehydrogenase	5.66	6.94
CAD1	O82515	Probable mannitol dehydrogenase	-2.06	-2.62
Os01g0794400	Q0JIL1	Probable nucleoredoxin 2	1.52	1.88
DMR6	Q9FLV0	Protein DOWNY MILDEW RESISTANCE 6	1.39	2.47
EX1	Q93YW0	Protein EXECUTER 1	-3.03	-3.92
HSA32	Q8GWL1	Protein HEAT-STRESS-ASSOCIATED 32	2.35	3.59
JOKA2	M1BJF6	Protein JOKA2	-2.16	-2.27
MPH1	Q9FL44	Protein MAINTENANCE OF PSII UNDER HIGH LIGHT 1	-4.69	-2.83
NIK2	Q8RY65	Protein NSP-INTERACTING KINASE 2	-1.19	-1.94
NIK3	Q93ZS4	Protein NSP-INTERACTING KINASE 3	-2.81	-2.61
SGT1	Q0JL44	Protein SGT1 homolog	-2.13	-1.96
SRC2	O04023	Protein SRC2 homolog	1.06	1.70
Ptma	P26350	Prothymosin alpha	8.97	8.23
At1g59780	Q9XIF0	Putative disease resistance protein At1g59780	1.62	2.47
At5g05400	Q9FLB4	Putative disease resistance protein At5g05400	1.70	2.32
RPP13L3	Q9STE7	Putative disease resistance RPP13-like protein 3	-1.78	-2.10
PDC2	Q9FFT4	Pyruvate decarboxylase 2	2.21	3.18
RLP51	Q9SN38	Receptor-like protein 51	-2.08	-3.85
RBOHC	Q2HXL0	Respiratory burst oxidase homolog protein C	8.85	4.06
RTNLB1	Q9SUR3	Reticulon-like protein B1	-2.99	-2.15
RTNLB2	Q9SUT9	Reticulon-like protein B2	-2.22	-0.92
RggA	sp|Q9SQ56	RGG repeats nuclear RNA binding protein A	-1.97	-2.27
STR9	O48529	Rhodanese-like domain-containing protein 9	-1.04	-2.74
STR15	Q38853	Rhodanese-like domain-containing protein 15	-2.47	-2.20
TOGT1	Q9AT54	Scopoletin glucosyltransferase	1.91	2.97
FSD2	Q9LU64	Superoxide dismutase [Fe] 2	-2.34	-2.55
N/A	P16064	Subtilisin inhibitor 1	1.97	3.31
N/A	Q43636	Thioredoxin H-type	-0.77	0.00
N/A	P29449	Thioredoxin H-type 1	-3.45	-3.45
At1g18250	P50699	Thaumatin-like protein	2.23	0.15
At1g08570	O64654	Thioredoxin-like 1-1	1.33	-0.09
THBS1	P07996	Thrombospondin-1	9.59	8.67
TDL1	Q1G3T1	TPD1 protein homolog 1	1.30	2.26
TSPO	O82245	Translocator protein homolog	5.74	6.98

TRPA1	O75762	Transient receptor potential cation channel subfamily A member 1	1.58	2.95
PHOS32	Q8VYN9	Universal stress protein PHOS32	-0.90	2.61
N/A	Q40316	Vestitone reductase	-4.86	-2.92
VIM	P08670	Vimentin	11.14	10.87
At1g70260	F4I5D5	WAT1-related protein At1g70260	-1.15	-5.26
WDR26	Q9FNN2	WD repeat-containing protein 26 homolog	1.20	2.44
WIN2	P09762	Wound-induced protein WIN2	-1.54	-1.73
SAP12	Q67YE6	Zinc finger AN1 domain-containing stress-associated protein 12	1.46	1.62
At2g40140	Q9XEE6	Zinc finger CCCH domain-containing protein 29	-3.49	-2.02

Table S4. Sixty-eight DEGs directly associated with soluble sugar and protein metabolism under 5% and 25% PEG vs. CK.

Gene name	SwissProt ID	Protein name	log_2FC (5% PEG vs. CK)	log_2FC (25% PEG vs. CK)
**Glucose (14)**				
pgdC	Q94KU1	6-phosphogluconate dehydrogenase, decarboxylating 1	− 2.24	− 2.70
SFR2	Q8L6H7	Beta-glucosidase-like SFR2, chloroplastic	− 2.40	− 1.52
GLC1	P52409	Glucan endo-1,3-beta-glucosidase	− 1.77	− 0.36
At5g58090	Q93Z08	Glucan endo-1,3-beta-glucosidase 6	− 1.06	− 1.73
At5g56590	Q9FJU9	Glucan endo-1,3-beta-glucosidase 13	− 0.39	− 0.34
HGN1	P52407	Glucan endo-1,3-beta-glucosidase, basic vacuolar isoform	− 2.76	− 4.08
At2g16790	Q9SLE0	Gluconokinase	1.00	1.94
G6pc2	Q9Z186	Glucose-6-phosphatase 2	9.21	8.46
CAISE5	Q5KTS5	Glucose and ribitol dehydrogenase	3.58	4.92
GAPA	P09043	Glyceraldehyde-3-phosphate dehydrogenase A	− 2.17	− 2.11
GAPB	P12859	Glyceraldehyde-3-phosphate dehydrogenase B	− 2.24	− 2.56
PGM1	Q9M4G4	Phosphoglucomutase	− 2.59	− 2.19
Gcg	P55095	Pro-glucagon	8.24	7.56
UGPA	P19595	UTP--glucose-1-phosphate uridylyltransferase	− 2.60	− 2.25
**Sucrose (6)**				
INVA	Q9FXA8	Alkaline/neutral invertase A	1.52	2.10
SUS1	P10691	Sucrose synthase	− 3.42	− 1.99
SUS2	O24301	Sucrose synthase 2	0.18	0.76
SPP1	Q5IH14	Sucrose-phosphatase 1	− 2.37	− 1.97
SPS3	Q8RY24	Probable sucrose-phosphate synthase 3	1.17	2.15
SPS4	F4JLK2	Probable sucrose-phosphate synthase 4	− 1.56	− 5.17
**Fructose (12)**				
PFK2	Q9FIK0	ATP-dependent 6-phosphofructokinase 2	1.24	1.79
Pfkl	P12382	ATP-dependent 6-phosphofructokinase	− 5.84	− 3.54
PFKP	Q01813	ATP-dependent 6-phosphofructokinase	8.16	7.97
INV1	P26792	Beta-fructofuranosidase, insoluble isoenzyme 1	− 2.42	− 1.89
fbp	Q7MYW6	Fructose-1,6-bisphosphatase class 1	− 2.62	− 3.27
FBP	P46275	Fructose-1,6-bisphosphatase	− 1.87	− 1.48
FBP2	Q42649	Fructose-1,6-bisphosphatase	− 1.97	− 1.85
ALFC	P16096	Fructose-bisphosphate aldolase	− 1.67	− 2.52
FBA1	Q9SJU4	Fructose-bisphosphate aldolase 1	− 2.04	− 2.30
FBA6	Q9SJQ9	Fructose-bisphosphate aldolase 6	− 2.09	− 2.11
ALDOA	P04075	Fructose-bisphosphate aldolase A	6.09	6.52
At4g10260	O82616	Probable fructokinase-5	1.19	2.82
**Galactose (4)**				
BGAL	P48980	Beta-galactosidase	− 2.37	− 3.44
BGAL3	Q9SCV9	Beta-galactosidase 3	− 2.88	− 2.32
GOLS1	O22893	Galactinol synthase 1	2.37	2.31
GOLS2	C7G304	Galactinol synthase 2	3.73	4.74
**Fucose (4)**				
OFUT7	B7ZWR7	O-fucosyltransferase 7	1.61	2.21
OFUT20	O64884	O-fucosyltransferase 20	− 1.97	− 3.80
OFUT31	Q7Y030	O-fucosyltransferase 31	− 2.31	− 0.47
OFUT39	Q0WUZ5	O-fucosyltransferase 39	1.01	1.61
**Trehalose (4)**				
TPS6	Q94AH8	Alpha,alpha-trehalose-phosphate synthase [UDP-forming] 6	− 2.16	− 2.34
TPPD	Q67XC9	Probable trehalose-phosphate phosphatase D	5.19	5.61
TPPG	Q9SUW0	Probable trehalose-phosphate phosphatase G	3.14	4.32
TPPA	O64896	Trehalose-phosphate phosphatase A	− 1.40	− 4.18
**Mannan (2)**				
MSR1	Q9LU40	Protein MANNAN SYNTHESIS-RELATED 1	− 3.07	− 2.39

MAN1	Q9FZ29	Mannan endo-1,4-beta-mannosidase 1	− 2.34	− 2.22
**Starch (6)**				
SBE1	P30924	1,4-alpha-glucan-branching enzyme	− 0.69	− 0.10
WAXY	Q00775	Granule-bound starch synthase 1	− 2.22	− 2.03
DSP4	G4LTX4	Phosphoglucan phosphatase DSP4	− 2.73	− 2.29
AMY2	Q8LFG1	Probable alpha-amylase 2	2.12	3.02
PTST	F4KFB3	Protein PTST homolog 3	1.72	2.73
SS3	Q43846	Soluble starch synthase 3	− 3.74	− 0.75
**Protein (16)**				
APA1	O65390	Aspartic proteinase A1	− 2.49	− 2.05
FTSH2	Q655S1	ATP-dependent zinc metalloprotease FTSH 2	− 2.73	− 2.91
FTSH5	Q8LQJ8	ATP-dependent zinc metalloprotease FTSH 5	− 3.48	− 2.30
RPT1	Q41365	26S proteasome regulatory subunit 7	− 2.45	− 2.69
CTSB	P07858	Cathepsin B	5.88	5.81
CYP-3	Q40143	Cysteine proteinase 3	− 2.38	− 2.38
At2g17570	Q8S2T1	Dehydrodolichyl diphosphate synthase 6	− 2.58	− 2.45
LOC109345795	Q42369	Gamma conglutin 1	1.44	2.62
3MMP	Q5XF51	Metalloendoproteinase 3-MMP	2.11	3.01
Os04g0650000	P25776	Oryzain alpha chain	1.79	3.05
PAG1	O24362	Proteasome subunit alpha type-3	− 2.22	− 1.82
RMD5	Q9T075	Protein RMD5 homolog	1.13	1.96
MPA1	Q8H0S9	Puromycin-sensitive aminopeptidase	− 2.86	− 2.62
SCPL34	Q0WPR4	Serine carboxypeptidase-like 34	− 1.18	− 2.58
SCPL40	Q0WRX3	Serine carboxypeptidase-like 40	− 1.44	− 7.11
At4g32940	Q39119	Vacuolar-processing enzyme gamma-isozyme	− 2.71	− 1.93

Table S5. One hundred and ninety-one genes associated with other primary metabolism at 5 and 25% PEG vs. CK.

Gene name	SwissProt ID	Protein name	log_2FC (5% PEG vs. CK)	log_2FC (25% PEG vs. CK)
**Carbohydrate metabolism (45)**				
ACOC	P49608	Aconitate hydratase, cytoplasmic	1.40	2.33
Aco2	Q99KI0	Aconitate hydratase, mitochondrial	-2.54	-2.63
ADH9	W8JDE0	Alcohol dehydrogenase 9	-2.26	-1.70
At4g22110	Q0V7W6	Alcohol dehydrogenase-like 5	-1.62	-4.38
At5g42250	Q9FH04	Alcohol dehydrogenase-like 7	-8.99	-5.11
PHSL	P27598	Alpha-1,4 glucan phosphorylase L isozyme, chloroplastic/amyloplastic	1.02	1.75
R1	Q8LPT9	Alpha-glucan water dikinase, chloroplastic	-2.46	-2.58
BMY1	O22585	Beta-amylase	-2.44	-2.05
BAM1	Q9LIR6	Beta-amylase 1, chloroplastic	-0.60	-0.43
CAHC	P27141	Carbonic anhydrase, chloroplastic	-2.15	-1.88
CP12-2	Q9LZP9	Calvin cycle protein CP12-2, chloroplastic	-2.63	-2.02
Cs	Q9CZU6	Citrate synthase, mitochondrial	-2.12	-2.64
Dlst	Q9D2G2	Dihydrolipoyllysine-residue succinyltransferase component of 2-oxoglutarate dehydrogenase complex, mitochondrial	-1.30	-2.05
FDH1	Q07511	Formate dehydrogenase, mitochondrial	1.46	2.47
FTHS	P28723	Formate--tetrahydrofolate ligase	-3.02	-1.76
BAM9	Q8VYW2	Inactive beta-amylase 9	-2.88	-2.59
Ins2	P01326	Insulin-2	7.96	7.26
Idh3a	Q9D6R2	Isocitrate dehydrogenase [NAD] subunit alpha, mitochondrial	-2.73	-2.30
Idh3g	P70404	Isocitrate dehydrogenase [NAD] subunit gamma 1, mitochondrial	-3.21	-2.99
GCL1	Q9FJN7	LanC-like protein GCL1	1.20	1.62
ARA1	O23461	L-arabinokinase	-2.28	-2.73
LDHB	Q4R5B6	L-lactate dehydrogenase B chain	9.12	9.27
MD1	Q9FSF0	Malate dehydrogenase	-2.25	-1.75
MDH1	O48902	Malate dehydrogenase [NADP], chloroplastic	-2.43	-3.87
MDHG	P46488	Malate dehydrogenase, glyoxysomal	-2.50	-2.05
MDH	P46487	Malate dehydrogenase, mitochondrial	-2.01	-1.86
Mdh2	P08249	Malate dehydrogenase, mitochondrial	-1.40	-2.13

MASY	P08216	Malate synthase, glyoxysomal	-1.51	-3.98
MAOX	P34105	NADP-dependent malic enzyme	-2.82	-1.99
PPCC	Q9FV65	Phosphoenolpyruvate carboxylase 2	-1.77	-1.64
PPC2	Q5GM68	Phosphoenolpyruvate carboxylase 2	1.05	1.53
PPC4	Q8GVE8	Phosphoenolpyruvate carboxylase 4	-1.18	-2.17
PGKH	Q42961	Phosphoglycerate kinase, chloroplastic	-1.87	-2.19
OsI_025867	A2YNH4	Probable 6-phosphogluconolactonase 2	1.09	2.08
RFS1	Q84VX0	Probable galactinol--sucrose galactosyltransferase 1	-1.26	-2.48
RFS2	Q94A08	Probable galactinol--sucrose galactosyltransferase 2	-0.93	-1.01
RFS5	Q9FND9	Probable galactinol--sucrose galactosyltransferase 5	2.55	3.29
Os08g0191100	Q6YZX6	Putative aconitate hydratase, cytoplasmic	-2.39	-2.03
Pdha1	P35486	Pyruvate dehydrogenase E1 component subunit alpha, somatic form, mitochondrial	-1.81	-2.16
S17P	O20252	Sedoheptulose-1,7-bisphosphatase, chloroplastic	-2.21	-2.24
SFGH	Q8LAS8	S-formylglutathione hydrolase	-2.89	-2.73
SDHAF2	Q9FI44	Succinate dehydrogenase assembly factor 2, mitochondrial	1.19	1.83
Sdhb	Q9CQA3	Succinate dehydrogenase [ubiquinone] iron-sulfur subunit, mitochondrial	-1.13	-1.79
Taldo1	Q93092	Transaldolase	-2.78	-2.63
Tkt	P40142	Transketolase	-3.71	-4.03
TKTC	Q43848	Transketolase, chloroplastic	-2.11	-2.54
**Glycolysis (14)**				
Ogdh	Q60597	2-oxoglutarate dehydrogenase, mitochondrial	-2.10	-2.48
PGM1	Q42908	2,3-bisphosphoglycerate-independent phosphoglycerate mutase	-2.08	-1.48
ENO1	P06733	Alpha-enolase	10.78	11.07
PGH1	P26300	Enolase	-2.11	-2.07
GAPDH	P04406	Glyceraldehyde-3-phosphate dehydrogenase	7.83	8.14
GAPC2	Q9FX54	Glyceraldehyde-3-phosphate dehydrogenase GAPC2, cytosolic	-2.32	-1.99
LDHA	P00338	L-lactate dehydrogenase A chain	10.41	10.38
TPI1	P60174	Triosephosphate isomerase	9.93	9.80
TPIP1	P48496	Triosephosphate isomerase, chloroplastic	-1.77	-1.74
TPIP1	P48495	Triosephosphate isomerase, cytosolic	-2.25	-2.31
PGK1	A5A6K4	Phosphoglycerate kinase 1	8.94	9.46
PGAM1	P18669	Phosphoglycerate mutase 1	9.26	9.27
PKM	P14618	Pyruvate kinase PKM	11.03	11.01
PDH-E1 ALPHA	O24457	Pyruvate dehydrogenase E1 component subunit alpha-3, chloroplastic	-2.83	-1.97
**Amino acid biosynthesis (52)**				
VIT_05s0020g04070	D7T737	1,2-dihydroxy-3-keto-5-methylthiopentene dioxygenase 1	-2.21	-2.33
At1g21400	Q9LPL5	2-oxoisovalerate dehydrogenase subunit alpha 1, mitochondrial	1.41	2.36
APR2	P92981	5'-adenylylsulfate reductase 2, chloroplastic	-2.28	-1.89
METE	Q42699	5-methyltetrahydropteroyltriglutamate--homocysteine methyltransferase	-2.52	-2.19
mtnN	P9WJM3	5'-methylthioadenosine/S-adenosylhomocysteine nucleosidase	-2.78	-2.58
ALS SURA	P09342	Acetolactate synthase 1, chloroplastic	-1.63	-2.03
SAHH	P35007	Adenosylhomocysteinase	-3.03	-1.96
LKR/SDH	Q9SMZ4	Alpha-aminoadipic semialdehyde synthase	3.68	5.19
Acy1	Q99JW2	Aminoacylase-1	-2.36	-2.30
GDCST	P49363	Aminomethyltransferase, mitochondrial	-2.18	-1.78
SPE1	Q39827	Arginine decarboxylase	-3.01	-2.69
ASNS	P31752	Asparagine synthetase [glutamine-hydrolyzing]	1.19	3.09
CTSD	P07339	Cathepsin D	8.85	8.31
CS1	Q42884	Chorismate synthase 1, chloroplastic	-3.27	-2.27
CGS1	P55217	Cystathionine gamma-synthase 1, chloroplastic	-1.98	-3.11

PGDH3	Q9LT69	D-3-phosphoglycerate dehydrogenase 3, chloroplastic	2.19	3.27
P5CS	O04015	Delta-1-pyrroline-5-carboxylate synthase	2.07	3.50
ETFB	Q9LSW8	Electron transfer flavoprotein subunit beta, mitochondrial	1.48	2.55
Os02g0220500	Q6YW46	Elongation factor 1-gamma 2	-2.40	-2.38
FAH	Q8RW90	Fumarylacetoacetase	1.02	1.67
DCE	P54767	Glutamate decarboxylase	-2.05	-1.70
GDH1	Q43314	Glutamate dehydrogenase 1	1.61	2.85
GGAT2	Q9S7E9	Glutamate--glyoxylate aminotransferase 2	-2.01	-1.70
GS1-1	P51118	Glutamine synthetase cytosolic isozyme 1	-2.20	-2.18
GLNA4	P15102	Glutamine synthetase leaf isozyme, chloroplastic	-2.06	-2.55
GSTF	P46423	Glutathione S-transferase	-1.16	-1.50
GST3	P46417	Glutathione S-transferase 3	3.12	3.44
GSTF10	P42761	Glutathione S-transferase F10	-2.46	-2.36
GSTL3	Q9LZ06	Glutathione S-transferase L3	1.22	1.91
GSTU10	Q9CA57	Glutathione S-transferase U10	2.70	2.86
GSTU7	Q9ZW24	Glutathione S-transferase U7	1.10	0.11
GSTX4	Q03666	Probable glutathione S-transferase	-1.74	-2.42
PARA	P25317	Probable glutathione S-transferase parA	1.47	1.27
GDCSH	Q39732	Glycine cleavage system H protein, mitochondrial	-2.32	-2.39
HMT1	A4ZGQ8	Homocysteine S-methyltransferase 1	-1.33	-2.85
HMT3	Q8LAX0	Homocysteine S-methyltransferase 3	1.58	3.22
HGO	Q9ZRA2	Homogentisate 1,2-dioxygenase	1.16	1.81
Os05g0125500	Q75IM9	Isovaleryl-CoA dehydrogenase, mitochondrial	2.05	2.72
At3g58610	Q05758	Ketol-acid reductoisomerase, chloroplastic	-1.52	-2.32
CAS1	Q1KLZ2	L-3-cyanoalanine synthase 1, mitochondrial	-3.02	-2.07
GLX1	O65398	Lactoylglutathione lyase GLX1	-2.00	-2.14
NNMT	P40261	Nicotinamide N-methyltransferase	8.59	10.48
At5g53970	Q9FN30	Probable aminotransferase TAT2	4.20	5.98
THA1	Q8RXU4	Probable low-specificity L-threonine aldolase 1	1.91	2.98
P4H7	Q8L970	Probable prolyl 4-hydroxylase 7	1.04	1.44
SAMDC	Q04694	S-adenosylmethionine decarboxylase proenzyme	2.34	3.08
metK	Q54F07	S-adenosylmethionine synthase	-7.58	-8.75
SAMS2	Q96552	S-adenosylmethionine synthase 2	-2.55	-1.99
SAM3	P43282	S-adenosylmethionine synthase 3	-2.26	-2.10
SAT5	Q42538	Serine acetyltransferase 5	1.02	1.82
SHM4	O23254	Serine hydroxymethyltransferase 4	-2.56	-1.65
TSB	O50046	Tryptophan synthase beta chain 2, chloroplastic	0.46	0.24
**Lipid and Fatty metbalism (74)**				
Agpat2	Q8K3K7	1-acyl-sn-glycerol-3-phosphate acyltransferase beta	-1.71	-1.97
KCS1	Q9MAM3	3-ketoacyl-CoA synthase 1	-1.83	-1.78
CUT1	Q9XF43	3-ketoacyl-CoA synthase 6	-2.17	-2.09
KCS11	O48780	3-ketoacyl-CoA synthase 11	-1.07	-2.61
KCS20	Q9FG87	3-ketoacyl-CoA synthase 20	-1.76	-2.03
Acaa2	Q8BWT1	3-ketoacyl-CoA thiolase, mitochondrial	-1.16	-1.59
PED1	Q56WD9	3-ketoacyl-CoA thiolase 2, peroxisomal	-2.09	-0.24
KAT5	Q570C8	3-ketoacyl-CoA thiolase 5, peroxisomal	1.93	3.03
KAS2	Q9C9P4	3-oxoacyl-[acyl-carrier-protein] synthase II, chloroplastic	-2.63	-2.05
DWF5	Q9LDU6	7-dehydrocholesterol reductase	-2.72	-3.88
CAC3	Q9LD43	Acetyl-coenzyme A carboxylase carboxyl transferase subunit alpha, chloroplastic	-2.61	-2.01
ACBP	O04066	Acyl-CoA-binding protein	-3.87	-3.52
Scd1	P13516	Acyl-CoA desaturase 1	-4.63	-4.90
ACX2	O65201	Acyl-coenzyme A oxidase 2, peroxisomal	1.17	2.08
Adipoq	Q60994	Adiponectin	-4.26	-4.32
FAR	Q9XGY7	Alcohol-forming fatty acyl-CoA reductase	-4.78	-2.57
AKR1B1	P15121	Aldo-keto reductase family 1 member B1	10.18	9.94
DOX2	Q9C9U3	Alpha-dioxygenase 2	3.78	4.83
Angptl4	Q9Z1P8	Angiopoietin-related protein 4	-2.42	-1.75
ACLB-2	Q9FGX1	ATP-citrate synthase beta chain protein 2	-2.75	-2.58

Cpt1b	Q924X2	Carnitine O-palmitoyltransferase 1, muscle isoform	-2.72	-3.04
Ech1	O35459	Delta(3,5)-Delta(2,4)-dienoyl-CoA isomerase, mitochondrial	-1.60	-1.96
DCI1	Q9FHR8	Delta(3,5)-Delta(2,4)-dienoyl-CoA isomerase, peroxisomal	-2.83	-2.64
FAD2	Q8GZC3	Delta(12)-fatty-acid desaturase FAD2	-2.24	-2.02
DGAT3	Q9C5W0	Diacylglycerol O-acyltransferase 3	-2.61	-2.25
Echs1	Q8BH95	Enoyl-CoA hydratase, mitochondrial	-1.73	-1.73
FAD4	Q9SZ42	Fatty acid desaturase 4, chloroplastic	3.25	4.94
Fasn	P19096	Fatty acid synthase	-4.87	-5.51
APG	Q9LU14	GDSL esterase/lipase APG	-1.01	-2.04
At1g74460	Q9CA68	GDSL esterase/lipase At1g74460	1.24	2.42
At2g30310	O22927	GDSL esterase/lipase At2g30310	3.62	4.67
At5g33370	Q8LB81	GDSL esterase/lipase At5g33370	-2.68	-1.73
At5g45670	Q9FK75	GDSL esterase/lipase At5g45670	-1.88	-3.31
At5g45960	Q9FJ40	GDSL esterase/lipase At5g45960	-1.00	-1.63
PLSB	Q39639	Glycerol-3-phosphate acyltransferase, chloroplastic	-2.35	-2.11
Gpd1	P13707	Glycerol-3-phosphate dehydrogenase [NAD(+)], cytoplasmic	-2.92	-3.53
GDPD3	Q680A6	Glycerophosphodiester phosphodiesterase GDPD3	1.55	2.54
Lipe	P54310	Hormone-sensitive lipase	-4.62	-4.70
Hadh	Q61425	Hydroxyacyl-coenzyme A dehydrogenase, mitochondrial	-1.13	-1.71
HMGS	P54873	Hydroxymethylglutaryl-CoA synthase	-3.35	-5.06
INPS1	Q9SSV4	Inositol-3-phosphate synthase	2.51	3.11
JMT	Q9SBK6	Jasmonate O-methyltransferase	1.08	2.10
LOX2.1	O24370	Linoleate 13S-lipoxygenase 2-1, chloroplastic	0.87	0.07
LACS6	Q8LPS1	Long chain acyl-CoA synthetase 6, peroxisomal	1.25	2.06
Os11g0516000	Q2R3K3	Long chain base biosynthesis protein 2a	-8.91	-2.08
Acadl	P51174	Long-chain specific acyl-CoA dehydrogenase, mitochondrial	-1.20	-1.69
MSBP2	Q9M2Z4	Membrane steroid-binding protein 2	-2.54	-2.15
FAD6C	P48627	Omega-6 fatty acid desaturase, chloroplastic	-1.83	-2.80
ADS3	Q949X0	Palmitoyl-monogalactosyldiacylglycerol delta-7 desaturase, chloroplastic	-1.18	-2.87
Pnpla2	Q8BJ56	Patatin-like phospholipase domain-containing protein 2	-2.55	-3.12
Plin1	Q8CGN5	Perilipin-1	-4.42	-4.66
ROD1	Q9LVZ7	Phosphatidylcholine:diacylglycerol cholinephosphotransferase 1	2.76	3.54
PI4KG7	Q9SI52	Phosphatidylinositol 4-kinase gamma 7	-2.42	-0.54
SAC4	Q7XZU1	Phosphoinositide phosphatase SAC4	-2.72	-3.34
At4g16820	O23522	Phospholipase A1-Ibeta2, chloroplastic	2.67	2.64
DALL4	Q941F1	Phospholipase A1-Igamma1, chloroplastic	3.53	3.10
DALL2	Q9C8J6	Phospholipase A1-Igamma3, chloroplastic	1.01	-1.24
PLD1	Q41142	Phospholipase D alpha 1	1.16	1.93
AAE16	Q9LK39	Probable acyl-activating enzyme 16, chloroplastic	1.87	2.22
CK1	Q9M9H6	Probable choline kinase 1	1.91	2.95
At4g09760	Q9SZ92	Probable choline kinase 3	1.49	1.12
GPAT3	Q9SYJ2	Probable glycerol-3-phosphate acyltransferase 3	-1.44	-3.11
AT1	Q9FJ72	Probable long-chain-alcohol O-fatty-acyltransferase 1	1.81	3.00
MP3	Q9SK39	Probable steroid-binding protein 3	-2.06	-2.10
At2g40090	O04212	Putative ABC1 protein At2g40090	1.06	1.63
YDL109C	Q12103	Putative lipase YDL109C	1.18	1.59
Bscl2	Q9Z2E9	Seipin	-2.42	-2.52
Acads	Q07417	Short-chain specific acyl-CoA dehydrogenase, mitochondrial	-1.64	-2.38
SDH	Q9FJ95	Sorbitol dehydrogenase	-3.12	-4.04
Thrsp	Q62264	Thyroid hormone-inducible hepatic protein	-1.90	-2.36
Hadha	Q8BMS1	Trifunctional enzyme subunit alpha, mitochondrial	-1.27	-1.92
Hadhb	Q99JY0	Trifunctional enzyme subunit beta, mitochondrial	-2.11	-2.48

Acadvl	P50544	Very long-chain specific acyl-CoA dehydrogenase, mitochondrial	-1.67	-1.86
HDLBP	Q00341	Vigilin	8.26	8.26
**Others (5)**				
HMGR	Q03163	3-hydroxy-3-methylglutaryl-coenzyme A reductase	-2.51	-2.22
NIR1	P38500	Ferredoxin--nitrite reductase, chloroplastic	-2.05	-2.24
NIA2	P08509	Nitrate reductase [NADH] 2	-2.01	-2.10
NUDT17	Q9ZU95	Nudix hydrolase 17, mitochondrial	-2.78	-0.53
PDCB4	Q93V72	PLASMODESMATA CALLOSE-BINDING PROTEIN 4	1.26	2.09

Table S6. Twelve DEGs directly associated with cell morphogenesis for seed germination under 5% and 25% PEG vs. CK.

Gene name	SwissProt ID	Protein name	log_2FC (5% PEG vs. CK)	log_2FC (25% PEG vs. CK)
GMPM1	Q01417	18 kDa seed maturation protein	4.83	5.43
ASP	P32765	21 kDa seed protein	2.24	2.96
ACT7	P53492	Actin-7	− 2.39	− 1.56
AC97	P30171	Actin-97	− 1.54	− 2.27
AP2	P47927	Floral homeotic protein APETALA 2	− 2.44	− 2.72
At4g25140	P29525	Oleosin 18.5 kDa	5.30	6.40
At5g40420	Q39165	Oleosin 21.2 kDa	5.32	7.37
OLE18	P21641	Oleosin Zm-II	1.26	2.03
SPD1	F4J3R7	Protein SEEDLING PLASTID DEVELOPMENT 1	1.67	1.00
SBP65	Q39846	Seed biotin-containing protein SBP65	6.08	7.53
pec2a1a	B3STU4	Vicilin Car i 2.0101	3.00	4.52
At2g18540	F4IQK5	Vicilin-like seed storage protein At2g18540	4.83	5.43

Table S7. One hundred and sixty four genes associated with other cell morphogenesis at 5 and 25% PEG vs. CK.

Gene name	SwissProt ID	Protein name	log_2FC (5% PEG vs. CK)	log_2FC (25% PEG vs. CK)
**Growth and development (87)**				
Apmap	Q9D7N9	Adipocyte plasma membrane-associated protein	-1.03	-1.97
AKR2A	Q9SAR5	Ankyrin repeat domain-containing protein 2A	-2.05	-1.72
CLPR1	Q9XJ35	ATP-dependent Clp protease proteolytic subunit-related protein 1	-2.04	-2.78
FTSH	O82150	ATP-dependent zinc metalloprotease FTSH, chloroplastic	-2.49	-2.35
DCR	Q9FF86	BAHD acyltransferase DCR	-0.41	-0.68
BSG	P35613	Basigin	9.05	8.85
BXL1	Q9FGY1	Beta-D-xylosidase 1	-0.12	1.65
CRCK2	Q8VZJ9	Calmodulin-binding receptor-like cytoplasmic kinase 2	-1.00	-1.12
MEE14	Q9XIM0	CCG-binding protein 1	-2.59	-2.28
CDC48	P54774	Cell division cycle protein 48 homolog	-2.66	-1.10
CDC48D	Q9SCN8	Cell division control protein 48 homolog D	-3.25	-2.02
CESA4	Q84JA6	Cellulose synthase A catalytic subunit 4 [UDP-forming]	-5.84	-2.27
CET1	Q9XH44	CEN-like protein 1	4.66	6.24
Cenpv	Q9CXS4	Centromere protein V	1.79	4.37
Bug22	Q9VKV8	Cilia- and flagella-associated protein 20	1.37	2.08
COBL7	Q8GZ17	COBRA-like protein 7	-1.65	-2.40
SCD2	Q8RWD5	Coiled-coil domain-containing protein SCD2	1.13	1.48
COL4A1	P02462	Collagen alpha-1(IV) chain	9.51	8.16
COL4A2	P08572	Collagen alpha-2(IV) chain	9.30	8.79
Cuzd1	P70412	CUB and zona pellucida-like domain-containing protein 1	9.32	8.63
CYCU4-1	O80513	Cyclin-U4-1	-3.13	-4.02
Dnajb13	Q80Y75	DnaJ homolog subfamily B member 13	1.23	2.41
ERDJ3A	Q9SR96	DnaJ protein ERDJ3A	1.96	3.08
DNJH	P43644	DnaJ protein homolog ANJ1	-0.69	-0.22
DRM1	O22611	Dormancy-associated protein 1	-2.45	-2.40
EPFL2	Q9T068	EPIDERMAL PATTERNING FACTOR-like protein 2	-1.17	-2.42
EPFL9	Q9SV72	EPIDERMAL PATTERNING FACTOR-like protein 9	-1.64	-3.94
EXO70A1	Q9LZD3	Exocyst complex component EXO70A1	4.10	1.72
EXLA2	Q9SVE5	Expansin-like A2	-1.15	-2.18
EXLB1	O23547	Expansin-like B1	3.67	5.49
FN1	P02751	Fibronectin	11.72	10.11

FRL3	Q67ZB3	FRIGIDA-like protein 3	-2.21	-2.49
FRL4A	Q9LUV4	FRIGIDA-like protein 4a	-1.97	-1.69
GNAI2	P04899	Guanine nucleotide-binding protein G(i) subunit alpha-2	8.57	7.97
GBLP	P93340	Guanine nucleotide-binding protein subunit beta-like protein	-2.46	-2.69
TEB	Q588V7	Helicase and polymerase-containing protein TEBICHI	2.08	2.89
PPF-1	Q9FY06	Inner membrane protein PPF-1, chloroplastic	-1.99	-2.59
IGFBP4	P22692	Insulin-like growth factor-binding protein 4	6.95	6.09
IFRD1	Q5S1U6	Interferon-related developmental regulator 1	2.86	4.15
KRT8	P05787	Keratin, type II cytoskeletal 8	8.57	8.83
LBD1	Q9LQR0	LOB domain-containing protein 1	4.15	4.28
LBD11	Q9SK08	LOB domain-containing protein 11	1.46	1.60
LBD27	Q9STS6	LOB domain-containing protein 27	-1.74	-3.78
LBD36	Q9FKZ3	LOB domain-containing protein 36	1.66	1.82
At1g80180	Q9SSC1	MAPK kinase substrate protein At1g80180	1.11	-0.51
MSN	P26038	Moesin	8.58	8.40
SKU5	Q9SU40	Monocopper oxidase-like protein SKU5	-2.86	-5.49
AHNAK	Q09666	Neuroblast differentiation-associated protein AHNAK	8.57	8.44
NAP1;2	Q70Z18	Nucleosome assembly protein 1;2	-2.51	-1.60
PVIP	Q84N38	OBERON-like protein	-2.92	-3.92
pald1	Q803E0	Paladin	-3.50	-8.56
DRP1E	Q9FNX5	Phragmoplastin DRP1E	-0.89	-0.35
BPS1	Q9LMM6	Protein BPS1, chloroplastic	-2.39	-1.97
BLT	F4I878	Protein BRANCHLESS TRICHOME	-2.21	-3.43
CRWN1	F4HRT5	Protein CROWDED NUCLEI 1	-2.45	-1.54
DMP3	Q9STW3	Protein DMP3	3.97	4.83
DMP9	Q9FK96	Protein DMP9	-1.66	-4.16
EXL5	Q9SII5	Protein EXORDIUM-like 5	-3.08	-3.16
GEX1	Q681K7	Protein GAMETE EXPRESSED 1	2.14	2.62
GIL1	F4KGE8	Protein GRAVITROPIC IN THE LIGHT 1	-1.06	-2.53
JGB	O48716	Protein JINGUBANG	-0.05	-0.05
KLCR2	Q9LII8	Protein KINESIN LIGHT CHAIN-RELATED 2	-3.25	-4.43
NOV	F4JTS8	Protein NO VEIN	1.14	1.40
PHI-1	O82161	Protein PHOSPHATE-INDUCED 1	-2.09	-2.59
SMG7L	Q9FZ99	Protein SMG7L	1.19	1.75
SP1L1	B3H4F1	Protein SPIRAL1-like 1	-2.94	-2.67
TBL20	Q9M896	Protein trichome birefringence-like 20	-2.62	-3.57
XCT	B8BDW1	Protein XAP5 CIRCADIAN TIMEKEEPER	-3.06	-2.93
PDF1	Q9S728	Protodermal factor 1	-3.23	-2.24
PLA1	Q8GT41	Putative invertase inhibitor	1.65	1.69
PSK6	Q8LA14	Putative phytosulfokines 6	1.52	2.21
RMI1	Q5XUX6	RecQ-mediated genome instability protein 1	1.04	1.36
At1g67360	Q9FYF7	REF/SRPP-like protein At1g67360	1.15	-1.44
RTNLB13	O64837	Reticulon-like protein B13	1.38	2.95
RTL1	Q52QI2	Retrotransposon-like protein 1	8.96	10.78
DOT2	Q9LFE0	SART-1 family protein DOT2	-2.25	-2.65
SBP2	Q93WN0	Selenium-binding protein 2	1.05	-0.30
DIN1	P27626	Senescence-associated protein DIN1	1.23	1.66
SPA15	Q9AXU3	Senescence-associated protein SPA15, chloroplastic	-2.71	-2.74
At1g47710	Q9S7T8	Serpin-ZX	-1.50	-1.89
At3g60660	Q9LZZ7	Spindle and kinetochore-associated protein 1 homolog	-1.81	-5.68
SPL8	Q8GXL3	Squamosa promoter-binding-like protein 8	1.05	1.21
SBT1.4	Q9LVJ1	Subtilisin-like protease SBT1.4	-2.44	-1.78
SBT1.6	O49607	Subtilisin-like protease SBT1.6	-2.21	-3.29
TGFBI	Q15582	Transforming growth factor-beta-induced protein ig-h3	9.61	9.74
TAGLN2	P37802	Transgelin-2	8.77	8.61
MSI4	O22607	WD-40 repeat-containing protein MSI4	-2.45	-2.78
**Cell wall（52)**				
CESA2	O48947	Cellulose synthase A catalytic subunit 2 [UDP-forming]	-2.80	-2.18
CESA3	Q941L0	Cellulose synthase A catalytic subunit 3 [UDP-forming]	-2.54	-2.33
CSLE1	Q8VZK9	Cellulose synthase-like protein E1	3.98	6.40
CSLG3	Q0WVN5	Cellulose synthase-like protein G3	-1.02	-1.37
CTL2	Q9LSP9	Chitinase-like protein 2	-2.70	-2.27
EXPA1	Q9C554	Expansin-A1	-1.26	-2.50
EXPA2	Q40636	Expansin-A2	1.32	1.93
EXPA4	O48818	Expansin-A4	-3.74	-3.09
FLA1	Q9FM65	Fasciclin-like arabinogalactan protein 1	-1.06	-3.32
FLA6	Q9SIL7	Fasciclin-like arabinogalactan protein 6	-3.31	-7.15

FLA12	Q8LEE9	Fasciclin-like arabinogalactan protein 12	-3.24	-2.53
GALS3	O65431	Galactan beta-1,4-galactosyltransferase GALS3	-2.24	-1.64
GDPDL4	Q9FJ62	Glycerophosphodiester phosphodiesterase GDPDL4	-1.83	-3.74
BC10	Q65XS5	Glycosyltransferase BC10	0.40	0.57
KIN4A	Q8GS71	Kinesin-like protein KIN-4A	-2.62	-2.62
LRX1	O65375	Leucine-rich repeat extensin-like protein 1	-1.16	-2.86
LRX3	Q9T0K5	Leucine-rich repeat extensin-like protein 3	-2.81	-2.22
LRX5	Q9SN46	Leucine-rich repeat extensin-like protein 5	-2.02	-4.61
HHT1	Q94CD1	Omega-hydroxypalmitate O-feruloyl transferase	0.07	0.63
PAE9	B9DFR3	Pectin acetylesterase 9	1.33	2.67
PAE12	Q9SFF6	Pectin acetylesterase 12	-2.66	-2.85
PGLR	P48979	Polygalacturonase	5.87	8.91
BXL2	Q94KD8	Probable beta-D-xylosidase 2	-1.81	-3.84
GATL3	Q0V7R1	Probable galacturonosyltransferase-like 3	-1.01	-2.77
GATL4	Q9M8J2	Probable galacturonosyltransferase-like 4	-1.02	-2.47
GATL9	O04536	Probable galacturonosyltransferase-like 9	-1.71	-1.56
At5g03795	Q9FFN2	Probable glycosyltransferase At5g03795	0.17	0.12
At4g24780	Q9C5M8	Probable pectate lyase 18	-1.50	1.94
PME53	Q8VYZ3	Probable pectinesterase 53	-1.23	-5.83
PME54	Q3E989	Probable pectinesterase/pectinesterase inhibitor 54	-1.95	-2.84
PME61	Q9FK05	Probable pectinesterase/pectinesterase inhibitor 61	-2.28	-2.24
XTH5	Q9XIW1	Probable xyloglucan endotransglucosylase/hydrolase protein 5	-2.11	-3.83
XTH8	Q8L9A9	Probable xyloglucan endotransglucosylase/hydrolase protein 8	-1.85	-4.37
XTH23	Q38910	Probable xyloglucan endotransglucosylase/hydrolase protein 23	-2.22	-3.64
XTH28	Q38909	Probable xyloglucan endotransglucosylase/hydrolase protein 28	-1.38	-1.08
XTH30	Q38908	Probable xyloglucan endotransglucosylase/hydrolase protein 30	-0.09	0.44
XTH33	Q8LC45	Probable xyloglucan endotransglucosylase/hydrolase protein 33	-7.25	-8.96
CSLC2	Q69L19	Probable xyloglucan glycosyltransferase 2	-2.32	-3.91
CSLC12	Q9ZQB9	Probable xyloglucan glycosyltransferase 12	-1.47	-3.01
AXY4L	Q9LRS2	Protein ALTERED XYLOGLUCAN 4-like	1.13	1.33
CSI1	F4IIM1	Protein CELLULOSE SYNTHASE INTERACTIVE 1	-3.12	-2.76
CER2	Q39048	Protein ECERIFERUM 2	1.46	2.18
IQD14	Q8LPG9	Protein IQ-DOMAIN 14	-1.97	-1.47
TBL39	Q9SIN2	Protein trichome birefringence-like 39	3.04	4.77
RHM1	Q9SYM5	Trifunctional UDP-glucose 4,6-dehydratase/UDP-4-keto-6-deoxy-D-glucose 3,5-epimerase/UDP-4-keto-L-rhamnose-reductase RHM1	-2.23	-1.79
AXS2	Q9SGE0	UDP-D-apiose/UDP-D-xylose synthase 2	-2.13	-2.48
XGD1	Q94AA9	Xylogalacturonan beta-1,3-xylosyltransferase	1.43	2.53
XTH2	P35694	Xyloglucan endotransglucosylase/hydrolase 2	-3.29	-3.16
XTH9	Q8LDW9	Xyloglucan endotransglucosylase/hydrolase protein 9	-1.92	-1.52
XTH31	P93046	Xyloglucan endotransglucosylase/hydrolase protein 31	2.74	3.32
CSLC4	Q9LJP4	Xyloglucan glycosyltransferase 4	-1.59	-3.47
MUR3	Q7XJ98	Xyloglucan galactosyltransferase MUR3	1.02	1.52
**Microtubule cytoskeleton organization (18)**				
AIR9	F4IIU4	187-kDa microtubule-associated protein AIR9	-5.27	-2.41
MAP65-6	Q9SIS3	65-kDa microtubule-associated protein 6	-3.35	-1.54
ACT1	Q05214	Actin	-2.81	-2.93
ACTB	O18840	Actin, cytoplasmic 1	11.86	10.90
ADF1	Q9FVI2	Actin-depolymerizing factor 1	-2.54	-2.35
CAP1	Q01518	Adenylyl cyclase-associated protein 1	8.73	7.78
COF1	Q4P6E9	Cofilin	-9.58	-9.58
CFL1	P23528	Cofilin-1	10.26	9.23
dlcB	Q86A88	Dynein light chain, cytoplasmic	4.00	4.36
MYH9	P35579	Myosin-9	7.77	6.78
PFN1	P07737	Profilin-1	10.53	10.25
WVD2	Q84ZT9	Protein WAVE-DAMPENED 2	1.36	1.96
RHOA	Q5REY6	Transforming protein RhoA	5.27	5.10
TMSB10	P63313	Thymosin beta-10	11.67	11.30
TUBA1B	P68361	Tubulin alpha-1B chain	4.63	3.87
TUBA5	B9DHQ0	Tubulin alpha-5 chain	-2.24	-2.43
TUBB	P93176	Tubulin beta chain	-1.87	-1.93
TUBB1	Q43594	Tubulin beta-1 chain	-1.99	-1.73
TUBB5	P69893	Tubulin beta-5 chain	9.99	9.39
**Cell death (6)**				
Cidec	P56198	Cell death activator CIDE-3	-3.39	-3.92
Cidea	O70302	Cell death activator CIDE-A	-6.22	-11.65
LGALS1	P09382	Galectin-1	10.79	9.52

SMG7	A9QM73	Protein SMG7	-2.61	-2.26
CYSEP	O65039	Vignain	3.15	2.08
SAG12	Q9FJ47	Senescence-specific cysteine protease SAG12	1.95	6.67

Table S8. Forty- two TFs directly associated with stress response and seed germination under 5% and 25% PEG vs. CK.

Gene name	SwissProt ID	Protein name	log_2FC (5% PEG vs. CK)	log_2FC (25% PEG vs. CK)
**MYB (9)**				
MYB4	Q7XBH4	Transcription factor MYB4	− 1.38	− 2.03
MYB20	Q9C7U7	Transcription factor MYB20	2.78	2.86
MYB73	O23160	Transcription factor MYB73	− 2.04	− 1.81
MYB77	Q9SN12	Transcription factor MYB77	− 1.36	− 3.31
MYB102	Q9LDR8	Transcription factor MYB102	3.15	3.80
MYB105	Q9SEZ4	Transcription factor MYB105	− 1.56	− 2.45
MYB108	Q9LDE1	Transcription factor MYB108	2.25	3.72
MYB330	P81395	Myb-related protein 330	1.06	1.68
MY1R1	Q2V9B0	Transcription factor MYB1R1	− 2.54	− 2.23
**BZIP (7)**				
BZIP02	Q5QNI5	Basic leucine zipper 2	− 2.37	− 4.95
BZIP9	Q9FUD3	Basic leucine zipper 9	1.31	1.99
BZIP34	F4IN23	Basic leucine zipper 34	− 1.67	− 5.46
BZIP11	O65683	bZIP transcription factor 11	2.22	3.20
BZIP44	C0Z2L5	bZIP transcription factor 44	2.24	3.67
BZIP53	Q9LZP8	bZIP transcription factor 53	1.97	2.95
BZIP60	Q9C7S0	bZIP transcription factor 60	1.04	1.30
**WRKY (5)**				
WRKY4	Q9XI90	Probable WRKY transcription factor 4	1.85	1.88
WRKY6	Q9C519	WRKY transcription factor 6	1.22	1.20
WRKY40	Q9SAH7	Probable WRKY transcription factor 40	− 0.63	0.74
WRKY71	Q93WV4	WRKY transcription factor 71	2.78	3.15
WRKY75	Q9FYA2	Probable WRKY transcription factor 75	4.03	5.61
**NAC (14)**				
NAC019	Q9C932	NAC domain-containing protein 19	4.52	5.72
NTL8	Q9XIN7	NAC domain-containing protein 40	2.21	3.43
NAC045	A4VCM0	NAC domain-containing protein 45	1.14	− 1.88
NAC048	Q7F2L3	NAC domain-containing protein 48	1.43	1.30
NAC076	O65508	NAC domain-containing protein 76	5.84	7.55
NAC087	Q9FK44	NAC domain-containing protein 87	1.40	2.62
NAC091	Q9LKG8	NAC domain-containing protein 91	0.81	1.02
NAC92	Q9FKA0	NAC domain-containing protein 92	2.44	3.65
NAC100	Q9FLJ2	NAC domain-containing protein 100	1.44	2.59
NAC101	Q9LVA1	NAC domain-containing protein 101	1.30	1.49
JA2L	A0A3Q7HH64	NAC domain-containing protein JA2L	1.09	1.58
NAC025	Q8GY42	NAC transcription factor 25	2.75	3.85
NAC047	Q84TD6	NAC transcription factor 47	2.20	3.44
NAC056	Q9LD44	NAC transcription factor 56	2.82	2.05
**BHLH (7)**				
BHLH60	Q3EAI1	Transcription factor bHLH60	− 2.06	− 3.16
BHLH62	Q9SRT2	Transcription factor bHLH62	− 1.06	− 1.96
BHLH68	Q8S3D1	Transcription factor bHLH68	− 2.55	− 0.23
BHLH94	Q9SK91	Transcription factor bHLH94	− 2.78	− 2.28
BHLH112	Q94JL3	Transcription factor bHLH112	1.63	1.91
BHLH128	Q8H102	Transcription factor bHLH128	− 1.09	− 1.61
BHLH143	Q9FY69	Transcription factor bHLH143	− 2.50	− 1.96

Table S9. Other one hundred and seventeen TFs at 5 and 25% PEG vs. CK.

Gene name	SwissProt ID	Protein name	log_2FC (5% PEG vs. CK)	log_2FC (25% PEG vs. CK)
**Zinc finger protein (21)**				

BBX32	Q9LJB7	B-box zinc finger protein 32	5.44	6.55
DOF3.4	Q39088	Dof zinc finger protein DOF3.4	1.01	1.52
DOF5.3	Q84TE9	Dof zinc finger protein DOF5.3	1.01	1.78
ATHB-12	Q9M276	Homeobox-leucine zipper protein ATHB-12	4.47	5.52
ATHB-16	Q940J1	Homeobox-leucine zipper protein ATHB-16	-2.56	-2.49
ATHB-6	P46668	Homeobox-leucine zipper protein ATHB-6	-2.07	-2.12
HAT22	P46604	Homeobox-leucine zipper protein HAT22	1.61	0.89
HOX16	A2X980	Homeobox-leucine zipper protein HOX16	1.38	1.64
TZF4	Q9ZWA1	Zinc finger CCCH domain-containing protein 2	5.93	7.71
Os02g0677700	Q0DYP5	Zinc finger CCCH domain-containing protein 17	1.99	1.85
At2g19810	O82199	Zinc finger CCCH domain-containing protein 20	2.61	4.03
O48772	O48772	Zinc finger CCCH domain-containing protein 26	1.15	1.35
ZHD4	Q9M9S0	Zinc-finger homeodomain protein 4	1.52	2.04
ZHD11	Q9SEZ1	Zinc-finger homeodomain protein 11	1.36	1.62
ZFP10	O80942	Zinc finger protein 10	-1.61	-2.55
COL2	Q96502	Zinc finger protein CONSTANS-LIKE 2	-1.37	-2.56
WIP2	Q9SVY1	Zinc finger protein WIP2	1.70	2.08
ZAT5	Q681X4	Zinc finger protein ZAT5	1.43	2.29
ZAT10	Q96289	Zinc finger protein ZAT10	2.78	3.50
ZAT11	Q9SLD4	Zinc finger protein ZAT11	2.50	4.01
ZAT12	Q42410	Zinc finger protein ZAT12	1.70	1.55
**MADS-box protein （3）**				
AGL12	Q38841	Agamous-like MADS-box protein AGL12	1.97	3.12
AGL16	A2RVQ5	Agamous-like MADS-box protein AGL16	1.30	1.91
MADS9	Q0HA25	Agamous-like MADS-box protein MADS9	1.47	1.10
**Others （93）**				
ABF4	Q9M7Q2	ABSCISIC ACID-INSENSITIVE 5-like protein 7	1.74	2.35
Ankrd13b	Q5F259	Ankyrin repeat domain-containing protein 13B	-2.23	-1.85
AHL11	Q8L7L5	AT-hook motif nuclear-localized protein 11	-2.15	-2.60
AHL20	Q8GWQ2	AT-hook motif nuclear-localized protein 20	1.49	5.48
AHL29	Q9C9K7	AT-hook motif nuclear-localized protein 29	1.30	2.52
ARID3	Q940Y3	AT-rich interactive domain-containing protein 3	-1.05	-1.98
At5g42700	Q9FMZ4	B3 domain-containing protein At5g42700	-1.21	-3.11
REM20	Q8LAV5	B3 domain-containing protein REM20	1.51	2.41
BLH1	Q9SJ56	BEL1-like homeodomain protein 1	-2.38	-1.35
CBP60E	F4IPM3	Calmodulin-binding protein 60 E	-1.34	-2.72
CCR4-2	Q9M2F8	Carbon catabolite repressor protein 4 homolog 2	-8.64	-3.74
cnot9	Q6NWL4	CCR4-NOT transcription complex subunit 9	-1.07	-2.88
C/VIF1	F4HWQ8	Cell wall / vacuolar inhibitor of fructosidase 1	4.76	3.86
CIGR1	Q69VG1	Chitin-inducible gibberellin-responsive protein 1	1.40	1.07
CPRF1	Q99089	Common plant regulatory factor 1	1.07	1.87
ATF4	P18848	Cyclic AMP-dependent transcription factor ATF-4	8.48	7.98
CDF5	Q9SEZ3	Cyclic dof factor 5	-2.49	-4.43

DREB1B	P93835	Dehydration-responsive element-binding protein 1B	4.24	3.87
DREB2D	Q9LQZ2	Dehydration-responsive element-binding protein 2D	5.84	6.73
DREB2G	P61827	Dehydration-responsive element-binding protein 2G	9.42	11.30
VCS	Q9LTT8	Enhancer of mRNA-decapping protein 4	-8.44	-3.70
EDRF1	Q3B7T1	Erythroid differentiation-related factor 1	1.16	1.70
Fhl1	P97447	Four and a half LIM domains protein 1	2.97	2.49
GATA8	Q9SV30	GATA transcription factor 8	-2.53	-3.88
HSFA2	O80982	Heat stress transcription factor A-2	1.59	1.85
HSFC1	Q9LV52	Heat stress transcription factor C-1	1.30	1.34
HD1	P46606	Homeobox protein HD1	-1.91	-1.76
KNAP2	O04135	Homeobox protein knotted-1-like 2	-2.42	-2.60
KNAP3	O04136	Homeobox protein knotted-1-like 3	-2.11	-2.11
LET12	O22300	Homeobox protein knotted-1-like LET12	-2.19	-2.64
HIRL1	Q6L4S3	Hypersensitive-induced response protein-like protein 1	-1.49	-3.01
HIF1A	Q16665	Hypoxia-inducible factor 1-alpha	8.06	8.06
At1g09660/At1g09670	Q8GWR3	KH domain-containing protein At1g09660/At1g09670	-2.71	-2.44
Letmd1	Q924L1	LETM1 domain-containing protein 1	-3.37	-4.37
LOXL2	Q9Y4K0	Lysyl oxidase homolog 2	9.68	8.95
MMP14	P50281	Matrix metalloproteinase-14	8.77	7.97
MBD10	Q9XI36	Methyl-CpG-binding domain-containing protein 10	-2.96	-1.86
MBF1C	Q9LV58	Multiprotein-bridging factor 1c	2.91	4.04
At1g73230	Q9CAT7	Nascent polypeptide-associated complex subunit beta	-2.11	-2.04
AFP2	Q9LMX5	Ninja-family protein AFP2	3.11	4.70
AFP3	Q94F39	Ninja-family protein AFP3	2.52	3.80
NFYA1	Q9LXV5	Nuclear transcription factor Y subunit A-1	3.36	4.54
NFYA3	Q93ZH2	Nuclear transcription factor Y subunit A-3	1.36	1.88
NFYB8	Q8VYK4	Nuclear transcription factor Y subunit B-8	1.12	1.66
NFYC2	Q8LCG7	Nuclear transcription factor Y subunit C-2	2.23	2.43
PTTG1IP	Q5NVI6	Pituitary tumor-transforming gene 1 protein-interacting protein	8.82	7.46
PRMT11	Q9SU94	Protein arginine N-methyltransferase 1.1	-2.31	-1.94
AGO1B	Q7XSA2	Protein argonaute 1B	-2.92	-2.20
BPC6	Q8L999	Protein BASIC PENTACYSTEINE6	-2.26	-2.83
FAR1	Q9SWG3	Protein FAR-RED IMPAIRED RESPONSE 1	-1.24	-2.41
G1L7	Q941W1	Protein G1-like7	-1.39	-1.57
IRO2	A2WZ60	Protein IRON-RELATED TRANSCRIPTION FACTOR 2	-1.76	-2.21
LSH6	Q9LMK2	Protein LIGHT-DEPENDENT SHORT HYPOCOTYLS 6	-1.01	-1.07
At2g05910	Q9ZUF7	Protein LURP-one-related 6	2.88	5.17
RL3	Q6NNN0	Protein RADIALIS-like 3	-1.24	-6.37
TIC	Q94KE2	Protein TIME FOR COFFEE	-2.35	-2.20
TPR2	Q0J7U6	Protein TOPLESS-RELATED PROTEIN 2	-3.42	-2.36
TPR1	Q5NBT9	Protein TPR1	-2.88	-3.13
CKB4	O80507	Putative casein kinase II subunit beta-4	-2.57	-2.87
SCL13	Q9M0M5	Scarecrow-like protein 13	-4.70	-2.38
SCL3	Q9LPR8	Scarecrow-like protein 3	1.26	1.41
PAT1	Q9LDL7	Scarecrow-like transcription factor PAT1	1.14	1.31
SPL1	Q9SMX9	Squamosa promoter-binding-like	-2.36	-2.57

		protein 1		
SPL14	Q8RY95	Squamosa promoter-binding-like protein 14	-9.02	-3.96
SPL16	Q700C2	Squamosa promoter-binding-like protein 16	-2.01	-2.05
TPR1	Q0WV90	Topless-related protein 1	-2.73	-1.83
TPR3	Q84JM4	Topless-related protein 3	-2.53	-1.71
Os07g0631100	Q8LHP0	Transcription elongation factor 1 homolog	-2.12	-0.48
TFIIS	Q9ZVH8	Transcription elongation factor TFIIS	3.09	3.88
BTF3	P20290	Transcription factor BTF3	-9.44	-9.44
DIVARICATA	Q8S9H7	Transcription factor DIVARICATA	1.13	1.06
GTE2	Q9LXA7	Transcription factor GTE2	-3.10	-2.39
GTE7	Q7Y214	Transcription factor GTE7	-1.83	-2.81
GTE8	Q9LK27	Transcription factor GTE8	-2.23	-1.47
HY5	O24646	Transcription factor HY5	1.02	1.24
IBL1	Q9M0B9	Transcription factor IBH1-like 1	-1.37	-2.74
RAX1	Q9FG68	Transcription factor RAX1	1.41	2.14
SRM1	Q9FNN6	Transcription factor SRM1	-2.01	-1.42
TCP4	Q8LPR5	Transcription factor TCP4	-1.85	-2.06
TCP23	Q9LQF0	Transcription factor TCP23	-2.21	-1.45
UNE12	O22768	Transcription factor UNE12	-3.44	-2.67
VOZ1	Q9SGQ0	Transcription factor VOZ1	-2.75	-3.75
OFP2	O04351	Transcription repressor OFP2	1.66	1.82
OFP7	Q9ZU65	Transcription repressor OFP7	1.63	1.03
OFP8	Q3E9B4	Transcription repressor OFP8	1.52	2.62
OFP13	Q9FMC8	Transcription repressor OFP13	-2.68	-4.64
LUG	Q9FUY2	Transcriptional corepressor LEUNIG	-2.61	-2.31
SEU	Q8W234	Transcriptional corepressor SEUSS	-3.44	-3.89
ARR1	Q940D0	Two-component response regulator ARR1	-1.93	-1.93
UBA2A	Q9LES2	UBP1-associated protein 2A	-1.79	-0.53
VQ11	Q9M8L3	VQ motif-containing protein 11	1.52	3.05
VQ19	Q9LDZ1	VQ motif-containing protein 19	1.92	2.91
VQ22	Q9LIE6	VQ motif-containing protein 22	2.86	3.14

Table S10. Fifty two DEGs directly associated with hormone response under 5% and 25% PEG vs. CK.

Gene name	SwissProt ID	Protein name	log_2FC (5% PEG vs. CK)	log_2FC (25% PEG vs. CK)
**GA (1)**				
GID1B	Q9LYC1	Gibberellin receptor GID1B	1.49	1.96
**IAA (19)**				
ABP19A	Q9ZRA4	Auxin-binding protein ABP19a	− 1.90	− 3.92
ABP20	O04011	Auxin-binding protein ABP20	− 1.69	− 1.83
AIR12	Q94BT2	Auxin-induced in root cultures protein 12	1.75	2.70
AUX22	P13088	Auxin-induced protein AUX22	− 1.48	− 3.91
AUX28	P13089	Auxin-induced protein AUX28	− 3.07	− 5.50
AX10A	P33080	Auxin-induced protein X10A	− 1.65	− 3.62
ARF5	P93024	Auxin response factor 5	1.04	1.59
ARF6	Q9ZTX8	Auxin response factor 6	− 2.48	− 2.05
IAA1	Q5VRD1	Auxin-responsive protein IAA1	8.18	9.72
IAA4	P33077	Auxin-responsive protein IAA4	− 1.30	− 1.78
IAA8	Q38826	Auxin-responsive protein IAA8	− 1.90	− 2.01
IAA14	Q38832	Auxin-responsive protein IAA14	− 1.90	− 1.90
IAA16	O24407	Auxin-responsive protein IAA16	− 2.07	− 1.60
IAA17	P93830	Auxin-responsive protein IAA17	2.33	2.96
SAUR21	Q9FJF9	Auxin-responsive protein SAUR21	− 1.57	− 3.58
SAUR32	Q9ZUZ3	Auxin-responsive protein SAUR32	− 1.17	− 0.66
SAUR36	O22150	Auxin-responsive protein SAUR36	1.29	1.68
SAUR76	Q29PU2	Auxin-responsive protein SAUR76	− 4.06	− 3.58
SAUR77	Q9LQI6	Auxin-responsive protein SAUR77	− 2.40	− 4.31
**CTK (4)**				
AHK2	Q9C5U2	Histidine kinase 2	− 1.79	− 2.15
AHP1	Q9ZNV9	Histidine-containing phosphotransfer protein 1	1.06	2.22
AHP4	Q9LU15	Histidine-containing phosphotransfer protein 4	3.04	3.80
ARR3	Q9ZWS9	Two-component response regulator ARR3	− 1.44	− 2.73
**JA (2)**				
CYP94B1	Q9FMV7	Cytochrome P450 94B1	2.43	4.00

GRXC9	Q9SGP6	Glutaredoxin-C9	− 1.02	− 1.33
**ABA (6)**				
CPN20	O65282	20 kDa chaperonin	− 2.09	− 1.60
PYL1	Q8VZS8	Abscisic acid receptor PYL1	− 1.12	− 3.86
PYL4	O80920	Abscisic acid receptor PYL4	− 2.21	− 4.28
CIP1	F4JZY1	COP1-interactive protein 1	− 1.17	− 2.26
NHL6	Q8LD98	NDR1/HIN1-like protein 6	2.31	3.29
NRP1	Q9ZQ80	Nodulin-related protein 1	1.25	− 0.91
**ETH (20)**				
ETR2	Q0WPQ2	Ethylene receptor 2	1.18	2.17
ERF.C.3	A0A3Q7I5Y9	Ethylene-response factor C3	− 1.87	− 2.32
ERF5	Q40478	Ethylene-responsive transcription factor 5	− 2.77	− 3.23
ERF1B	Q8LDC8	Ethylene-responsive transcription factor 1B	2.25	3.32
ERF3	Q9SXS8	Ethylene-responsive transcription factor 3	1.30	− 0.33
ERF4	Q40477	Ethylene-responsive transcription factor 4	1.71	1.76
ERF12	Q94ID6	Ethylene-responsive transcription factor 12	1.58	1.67
CRF2	Q9SUQ2	Ethylene-responsive transcription factor CRF2	− 1.15	− 2.59
ERF012	Q9SFE4	Ethylene-responsive transcription factor ERF012	− 4.10	− 3.80
ERF039	Q9SUK8	Ethylene-responsive transcription factor ERF039	1.54	3.00
ERF053	Q9SKT1	Ethylene-responsive transcription factor ERF053	4.37	4.05
ERF071	O22259	Ethylene-responsive transcription factor ERF071	− 2.47	− 2.87
ERF106	Q9LY05	Ethylene-responsive transcription factor ERF106	− 2.13	− 2.09
ERF110	Q70II3	Ethylene-responsive transcription factor ERF110	3.59	4.42
ERF113	Q9LYU3	Ethylene-responsive transcription factor ERF113	2.95	4.43
ERN1	A2Q5W1	Ethylene-responsive transcription factor ERN1	1.85	2.98
RAP2-2	Q9LUM4	Ethylene-responsive transcription factor RAP2-2	− 2.65	− 2.39
RAP2-4	Q8H1E4	Ethylene-responsive transcription factor RAP2-4	− 2.35	− 2.63
TINY	Q39127	Ethylene-responsive transcription factor TINY	− 3.32	− 4.86
EIN3	O24606	Protein ETHYLENE INSENSITIVE 3	− 2.40	− 2.21

Table S11. One hundred and sixty-seven genes associated with other bio-signaling at 5 and 25% PEG vs. CK.

Gene name	SwissProt ID	Protein name	log_2FC (5% PEG vs. CK)	log_2FC (25% PEG vs. CK)
**Protein phosphatase (19)**				
Phospho1	Q8R2H9	Phosphoethanolamine/phosphocholine phosphatase	-2.96	-3.31
PAP27	Q5MAU8	Probable inactive purple acid phosphatase 27	3.22	4.49
At1g47380	Q9FX08	Probable protein phosphatase 2C 12	-1.51	-2.16
At1g48040	Q9LNF4	Probable protein phosphatase 2C 13	1.11	1.55
PPC4-2	O81716	Probable protein phosphatase 2C 21	-2.81	-1.92
At2g29380	Q9ZW21	Probable protein phosphatase 2C 24	3.28	4.47
At2g30020	O80871	Probable protein phosphatase 2C 25	1.79	1.07
PPC6-1	Q9M8R7	Probable protein phosphatase 2C 33	-2.39	-0.63
PP2C6	Q94CL8	Probable protein phosphatase 2C 48	-2.11	-2.23
At3g62260	Q3EAF9	Probable protein phosphatase 2C 49	1.28	2.14
Os06g0717800	Q5Z8P0	Probable protein phosphatase 2C 60	-3.22	-2.38
PAP20	Q9LXI7	Probable purple acid phosphatase 20	3.01	4.74
PP2CA	P49598	Protein phosphatase 2C 37	1.93	3.23
PP2C51	Q65XK7	Protein phosphatase 2C 51	7.38	8.81
ABI1	P49597	Protein phosphatase 2C 56	3.08	4.30
ABI2	O04719	Protein phosphatase 2C 77	1.72	2.63
At2g20050/At2g20040	Q9SL76	Protein phosphatase 2C and cyclic nucleotide-binding/kinase domain-containing protein	-2.62	-1.99
IP5P2	Q9FUR2	Type I inositol polyphosphate 5-phosphatase 2	-1.12	-1.71
IP5P11	Q5EAF2	Type IV inositol polyphosphate 5-phosphatase 11	1.47	2.46
**Protein kinase (51)**				
CIPK11	O22932	CBL-interacting serine/threonine-protein kinase 11	-0.99	1.01
CIPK25	Q8W1D5	CBL-interacting serine/threonine-protein kinase 25	2.03	3.09
SMR5	Q9LNX4	Cyclin-dependent protein kinase inhibitor SMR5	4.86	5.92
SMR13	F4IWB3	Cyclin-dependent protein kinase inhibitor SMR13	1.43	2.16
CRK10	Q8GYA4	Cysteine-rich receptor-like protein kinase 10	-1.14	-3.29
CRK18	Q8RX80	Cysteine-rich receptor-like protein kinase 18	-2.22	-2.06
CRK25	Q9M0X5	Cysteine-rich receptor-like protein kinase 25	1.27	-0.52
At2g19130	O64477	G-type lectin S-receptor-like serine/threonine-protein kinase At2g19130	-1.37	-1.66
At4g27290	O81832	G-type lectin S-receptor-like serine/threonine-protein kinase At4g27290	-1.72	-2.37
At5g24080	Q9FLV4	G-type lectin S-receptor-like serine/threonine-protein kinase At5g24080	3.36	4.38
SD11	O81833	G-type lectin S-receptor-like serine/threonine-protein kinase SD1-1	1.51	-3.88
LRK10L-1.3	Q8VYG0	LEAF RUST 10 DISEASE-RESISTANCE LOCUS RECEPTOR-LIKE	-1.51	-1.31

		PROTEIN KINASE-like 1.3		
LRK10L-2.4	F4HQ22	LEAF RUST 10 DISEASE-RESISTANCE LOCUS RECEPTOR-LIKE PROTEIN KINASE-like 2.4	-1.82	-4.34
PHY1	P25848	Light-sensor Protein kinase	1.06	1.74
SERK2	Q7XV05	LRR receptor kinase SERK2	1.31	0.23
Q9FL28	Q9FL28	LRR receptor-like serine/threonine-protein kinase FLS2	-2.23	-4.19
RGI2	C0LGV1	LRR receptor-like serine/threonine-protein kinase RGI2	-1.07	-2.30
MPK19	Q9LUC3	Mitogen-activated protein kinase 19	-2.08	-1.85
MPK1	Q40884	Mitogen-activated protein kinase homolog 1	5.76	6.60
map3k13-b	A7J1T0	Mitogen-activated protein kinase kinase kinase 13-B	1.16	1.96
LRR-RLK	C0LGN2	Probable leucine-rich repeat receptor-like serine/threonine-protein kinase At3g14840	-1.88	-2.16
At1g12460	C0LGE4	Probable LRR receptor-like serine/threonine-protein kinase At1g12460	-1.17	-3.24
At1g53440	C0LGG9	Probable LRR receptor-like serine/threonine-protein kinase At1g53440	-1.00	-2.99
At1g56130	C0LGH2	Probable LRR receptor-like serine/threonine-protein kinase At1g56130	-1.21	-2.08
At1g56140	C0LGH3	Probable LRR receptor-like serine/threonine-protein kinase At1g56140	-1.26	-1.63
At1g74360	C0LGJ1	Probable LRR receptor-like serine/threonine-protein kinase At1g74360	2.77	3.52
At1g30570	Q9SA72	Probable receptor-like protein kinase At1g30570	-1.63	-1.80
WNK5	Q9SCU5	Probable serine/threonine-protein kinase WNK5	-2.93	-1.91
WNK11	Q6ICW6	Probable serine/threonine-protein kinase WNK11	1.21	1.82
FAB1D	Q9XID0	Putative 1-phosphatidylinositol-3-phosphate 5-kinase FAB1D	1.81	3.20
CRK30	Q9LDT0	Putative cysteine-rich receptor-like protein kinase 30	-1.47	-2.16
CRK35	Q9LDQ3	Putative cysteine-rich receptor-like protein kinase 35	-1.21	-1.84
At1g80870	Q9SAH3	Putative receptor-like protein kinase At1g80870	-1.19	-2.57
Os06g0291600	Q0DCT8	Protein kinase G11A	-2.56	-2.28
TMK4	Q9LK43	Receptor-like kinase TMK4	-1.03	-1.17
RLK7	F4I2N7	Receptor-like protein kinase 7	1.51	2.20
THE1	Q9LK35	Receptor-like protein kinase THESEUS 1	-2.27	-3.31
O81905	O81905	"Receptor-like serine/threonine-protein kinase SD1-8	2.61	3.14
Rack1	P63245	Receptor of activated protein C kinase 1	9.89	10.08
TMK1	P43298	Receptor protein kinase TMK1	-1.51	-2.23
CEPR2	Q9C7T7	Receptor protein-tyrosine kinase CEPR2	-2.28	-2.45
ATPK2	Q39030	Serine/threonine-protein kinase AtPK2/AtPK19	8.75	8.58
fray2	Q551H4	Serine/threonine-protein kinase fray2	1.43	2.32
SAPK2	A2YNT8	Serine/threonine-protein kinase SAPK2	1.49	2.46
SAPK3	P0C5D6	Serine/threonine-protein kinase SAPK3	1.78	2.54
SRK2E	Q940H6	Serine/threonine-protein kinase SRK2E	-2.39	-1.98
STN7	Q9S713	Serine/threonine-protein kinase STN7, chloroplastic	-2.22	-2.61
PP2A2	Q10BT5	Serine/threonine-protein phosphatase PP2A-2 catalytic subunit	-2.37	-2.62
NFP	Q0GXS4	Serine/threonine receptor-like kinase NFP	1.09	1.76
ASK10	Q39019	Shaggy-related protein kinase kappa	-2.42	-2.65
ASK6	Q39010	Shaggy-related protein kinase zeta	-3.13	-2.45
**Others (97)**				
TFT10	P93207	14-3-3 protein 10	-2.25	-2.48
YWHAQ	Q5RFJ2	14-3-3 protein theta	8.23	8.67
YWHAZ	Q5R651	14-3-3 protein zeta/delta	8.80	8.61
APS1	P27061	Acid phosphatase 1	-1.55	-2.40
Cryab	P23927	Alpha-crystallin B chain	3.74	3.62
APP	P05067	Amyloid-beta precursor protein	9.20	9.09
APLP2	Q06481	Amyloid-like protein 2	8.56	8.48
pstI	P08594	Aqualysin-1	-8.98	-8.87
At5g10080	Q9LX20	Aspartic proteinase-like protein 1	1.57	2.18
APF1	Q8VYV9	Aspartyl protease family protein 1	2.17	1.68
BSP	C0HJG8	Basic secretory protease	-1.06	-2.01
TMBIM6	P55061	Bax inhibitor 1	8.46	7.85
BAK1	Q94F62	BRASSINOSTEROID INSENSITIVE 1-associated receptor kinase 1	1.30	2.15
CBL3	Q75LU8	Calcineurin B-like protein 3	-3.91	-2.69
CAM81	P62199	Calmodulin-1	-2.58	-2.66
CAM2	A2Y609	Calmodulin-2	-2.05	-1.87
Calm2	P0DP30	Calmodulin-2	5.34	4.46
CALX	Q39817	Calnexin homolog	0.30	-0.46
CAPNS1	P06813	Calpain small subunit 1	8.64	8.79
CNN3	Q15417	Calponin-3	8.20	7.98
CALR	P27797	Calreticulin	10.19	9.42
HD16	Q852L0	Casein kinase 1-like protein HD16	-2.38	-1.60
POPTRDRAFT_820933	B9HMP5	CASP-like protein 1D1	1.90	3.99
CXIP4	Q84Y18	CAX-interacting protein 4	-2.33	-2.47

CCN1	O00622	CCN family member 1	8.96	9.02
CD151	P48509	CD151 antigen	9.35	9.37
CRK7	Q9LET1	CDPK-related kinase 7	-4.42	-3.16
Chga	P26339	Chromogranin-A	8.30	5.29
COTL1	Q14019	Coactosin-like protein	8.86	8.45
COL1A1	P02452	Collagen alpha-1(I) chain	9.48	6.59
CDKF-4	Q6Z8C8	Cyclin-dependent kinase F-4	-2.47	-0.12
CDKG-2	Q7XUF4	Cyclin-dependent kinase G-2	-2.31	-2.43
DGK4	Q1PDI2	Diacylglycerol kinase 4	-2.47	-2.77
At1g78850	Q9ZVA4	EP1-like glycoprotein 3	-1.87	-3.36
FLA8	O22126	Fasciclin-like arabinogalactan protein 8	-1.92	-2.95
FLZ15	Q9FH22	FCS-Like Zinc finger 15	2.41	3.73
FLNA	P21333	Filamin-A	7.48	6.74
GLR2.7	Q8LGN0	Glutamate receptor 2.7	-1.20	-3.78
GRDP1	Q9ZQ47	Glycine-rich domain-containing protein 1	-1.73	-1.88
GNB1	P62871	Guanine nucleotide-binding protein G(I)/G(S)/G(T) subunit beta-1	2.51	2.42
Gnas	P63095	Guanine nucleotide-binding protein G(s) subunit alpha isoforms short	-1.73	-2.19
HIPP24	O81464	Heavy metal-associated isoprenylated plant protein 24	2.29	3.01
HIPP27	Q67ZW1	Heavy metal-associated isoprenylated plant protein 27	1.90	3.40
HIPP39	O03982	Heavy metal-associated isoprenylated plant protein 39	2.09	3.21
MT-RNR2	Q8IVG9	Humanin	7.60	8.22
HIR1	Q5GI04	Hypersensitive-induced reaction 1 protein	-1.16	-1.69
VIP2	Q84WW3	Inositol hexakisphosphate and diphosphoinositol-pentakisphosphate kinase VIP2	-1.72	-1.87
IGFBP7	Q16270	Insulin-like growth factor-binding protein 7	9.97	10.38
ITGA3	P26006	Integrin alpha-3	9.59	8.90
ITGB1	P05556	Integrin beta-1	10.08	9.35
Reg1	P43137	Lithostathine-1	3.64	3.21
Reg2	Q08731	Lithostathine-2	5.71	5.31
METAL1	P43396	Metallothionein-like protein 1	-2.42	-2.15
MTI	P30564	Metallothionein-like protein type 2	10.18	11.94
MOB1-A	Q949G5	MOB kinase activator-like 1A	-2.14	-2.85
MYL6	Q5R844	Myosin light polypeptide 6	3.61	2.64
Myl2	P51667	Myosin regulatory light chain 2, ventricular/cardiac muscle isoform	9.49	8.97
ChlADR1	Q9FZ42	NADPH-dependent aldehyde reductase 1, chloroplastic	7.75	9.54
OLE1	Q43804	Oleosin 1	5.31	7.03
SPP1	P10451	Osteopontin	8.83	10.49
PPIA	Q0ZQL1	Peptidyl-prolyl cis-trans isomerase A	5.79	5.91
PPCK1	Q9SPK4	Phosphoenolpyruvate carboxylase kinase 1	1.40	2.73
PNSL4	Q9SCY3	Photosynthetic NDH subunit of lumenal location 4, chloroplastic	-2.98	-2.64
PHOT2	P93025	Phototropin-2	-2.47	-1.97
SERPINE1	P05121	Plasminogen activator inhibitor 1	11.12	9.89
TPS7	Q9LMI0	Probable alpha,alpha-trehalose-phosphate synthase [UDP-forming] 7	-2.83	-2.24
CML21	Q52K82	Probable calcium-binding protein CML21	3.61	5.34
CML27	Q9LE22	Probable calcium-binding protein CML27	1.89	1.35
CML41	Q8L3R2	Probable calcium-binding protein CML41	1.54	2.72
LAMP1	Q7Y228	Probable glutamate carboxypeptidase LAMP1	-1.26	-1.83
MAKR4	O80624	Probable membrane-associated kinase regulator 4	1.29	1.82
MAKR6	Q84JK8	Probable membrane-associated kinase regulator 6	-2.64	-3.97
PAP14	Q9LV04	Probable plastid-lipid-associated protein 14, chloroplastic	2.51	2.75
DAD2	J9U5U9	Probable strigolactone esterase DAD2	-2.04	-2.27
PSAP	P07602	Prosaposin	6.71	6.80
BRI1	O22476	Protein BRASSINOSTEROID INSENSITIVE 1	-1.11	-1.92
CAR5	Q9LP65	Protein C2-DOMAIN ABA-RELATED 5	1.10	2.34
EXO	Q9ZPE7	Protein EXORDIUM	3.91	5.14
FAF2	Q8GXU9	Protein FANTASTIC FOUR 2	-1.10	-5.57
MFT	Q9XFK7	Protein MOTHER of FT and TFL1	5.49	6.75
NDL2	Q9ASU8	Protein NDL2	-3.26	-4.70
S100A6	P06703	Protein S100-A6	10.85	11.05
SRF6	Q9C8M9	Protein STRUBBELIG-RECEPTOR FAMILY 6	-1.65	-1.97
At4g27745	Q9T096	Protein yippee-like At4g27745	1.56	2.11
RALF	Q945T0	Rapid alkalinization factor	-2.52	-2.70
Rhoc	Q62159	Rho-related GTP-binding protein RhoC	8.81	8.62
Scg2	Q03517	Secretogranin-2	6.78	4.14
SCPL49	P32826	Serine carboxypeptidase-like 49	-0.83	0.57
Sparc	P07214	SPARC	-2.28	-2.11
TPT1	P13693	Translationally-controlled tumor protein	10.71	11.06

TCTP	Q5J907	Translationally-controlled tumor protein homolog	-2.00	-2.48
TPM4	P67937	Tropomyosin alpha-4 chain	9.01	8.44
UCC1	O82081	Uclacyanin 1	-2.94	-8.18
ABCI8	Q9ZS97	UPF0051 protein ABCI8, chloroplastic	-2.07	-2.54
PLAU	P00749	Urokinase-type plasminogen activator	9.36	8.34
VLN2	O81644	Villin-2	-3.07	-2.57
WAKL8	Q9SA25	Wall-associated receptor kinase-like 8	-1.79	-1.87

Table S12. Ninety four genes associated with photosynthesis and energy at 5 and 25% PEG vs. CK.

Gene name	SwissProt ID	Protein name	log_2FC (5% PEG vs. CK)	log_2FC (25% PEG vs. CK)
**Photosynthesis (48)**				
CB12	P13869	Chlorophyll a-b binding protein, chloroplastic	-1.84	-2.90
LHCA1	Q01667	Chlorophyll a-b binding protein 6, chloroplastic	-1.12	-3.40
CAB6A	P12360	Chlorophyll a-b binding protein 6A, chloroplastic	-1.79	-1.92
CAB8	P27522	Chlorophyll a-b binding protein 8, chloroplastic	-1.73	-2.63
CAB13	P27489	Chlorophyll a-b binding protein 13, chloroplastic	-1.20	-4.33
CAB21	P27493	Chlorophyll a-b binding protein 21, chloroplastic	-2.02	-5.32
CAB-151	P27518	Chlorophyll a-b binding protein 151, chloroplastic	-1.52	-5.89
LHCB5	Q9XF89	Chlorophyll a-b binding protein CP26, chloroplastic	-1.61	-3.20
LHCB4.1	Q07473	Chlorophyll a-b binding protein CP29.1, chloroplastic	-1.72	-2.90
lhcA-P4	Q9SQL2	Chlorophyll a-b binding protein P4, chloroplastic	-1.53	-2.82
CAO	Q8S7E1	Chlorophyllide a oxygenase, chloroplastic	-2.66	-1.80
HEMB1	Q9SFH9	Delta-aminolevulinic acid dehydratase 1, chloroplastic	-2.30	-1.85
ELIP1	P93735	Early light-induced protein 1, chloroplastic	6.40	6.72
PETH	O04977	Ferredoxin--NADP reductase, leaf-type isozyme, chloroplastic	-2.03	-2.28
FTRC2	P41349	Ferredoxin-thioredoxin reductase catalytic chain, chloroplastic	-2.27	-2.40
GSA	Q40147	Glutamate-1-semialdehyde 2,1-aminomutase, chloroplastic	-1.78	-2.50
HEMA1	P93111	Glutamyl-tRNA reductase 1, chloroplastic	-1.98	-2.24
HPR	Q9C9W5	Glycerate dehydrogenase HPR, peroxisomal	-2.17	-2.11
GLO1	Q9LRR9	Glycolate oxidase 1	-2.34	-2.13
At3g10130	Q9SR77	Heme-binding-like protein At3g10130, chloroplastic	1.06	1.01
LIR1	Q03200	Light-regulated protein, chloroplastic	-4.67	-2.03
CHLH	Q9FNB0	Magnesium-chelatase subunit ChlH, chloroplastic	-2.81	-2.71
CRD1	Q6SJV8	Magnesium-protoporphyrin IX monomethyl ester [oxidative] cyclase, chloroplastic	-2.24	-2.07
PSBP2	P18212	Oxygen-evolving enhancer protein 2-2, chloroplastic	-2.17	-2.36
GOX	P05414	Peroxisomal (S)-2-hydroxy-acid oxidase	-1.13	-2.61
PAO	Q9FYC2	Pheophorbide a oxygenase, chloroplastic	1.36	2.36
PPH	Q9FFZ1	Pheophytinase, chloroplastic	2.86	4.25
PGLP1B	P0DKC4	Phosphoglycolate phosphatase 1B, chloroplastic	-1.81	-2.02
psaD	P12372	Photosystem I reaction center subunit II, chloroplastic	-2.25	-2.26
PSAF	P46486	Photosystem I reaction center subunit III, chloroplastic	-2.07	-1.89
PSAEA	Q41228	Photosystem I reaction center subunit IV A, chloroplastic	-2.05	-2.10
PSAN	P49107	Photosystem I reaction center subunit N, chloroplastic	-1.50	-3.11
PSAK	Q9SUI5	Photosystem I reaction center subunit psaK, chloroplastic	-1.61	-2.95
PSAG	Q9S7N7	Photosystem I reaction center subunit V, chloroplastic	-1.51	-2.94
PSAH	O04006	Photosystem I reaction center subunit VI, chloroplastic	-2.32	-1.88
PSAO	Q949Q5	Photosystem I subunit O	-1.73	-3.27
PST2	B3EWI4	Photosystem II 5 kDa protein, chloroplastic	-1.69	-3.30
PSBR	P06183	Photosystem II 10 kDa polypeptide, chloroplastic	-2.00	-2.46
PSBS	P54773	Photosystem II 22 kDa protein, chloroplastic	-1.94	-1.77
psbJ	Q49KY4	Photosystem II reaction center protein J	1.35	2.36
PSBW	Q39194	Photosystem II reaction center W protein,	-1.93	-1.95

		chloroplastic		
PPH1	P49599	Protein phosphatase 2C 57	-2.30	-1.86
SGR1	Q4JFW8	Protein STAY-GREEN 1, chloroplastic	3.29	4.58
THF1	Q7XAB8	Protein THYLAKOID FORMATION1, chloroplastic	-2.38	-1.67
POR1	Q9SDT1	Protochlorophyllide reductase, chloroplastic	-2.18	-2.12
RBCS	Q02980	Ribulose bisphosphate carboxylase small subunit, chloroplastic	-2.65	-2.44
SEP2	Q9SJ02	Stress enhanced protein 2, chloroplastic	1.14	1.71
VDE1	Q9SM43	Violaxanthin de-epoxidase, chloroplastic	4.66	5.93
**Energy (46)**				
At3g08860	Q9SR86	Alanine--glyoxylate aminotransferase 2 homolog 3, mitochondrial	2.66	4.04
CD4B	P31542	ATP-dependent Clp protease ATP-binding subunit ClpA homolog CD4B, chloroplastic	-2.24	-2.07
FTSH6	Q1PDW5	ATP-dependent zinc metalloprotease FTSH 6, chloroplastic	2.98	4.43
atpA	Q6L3A1	ATP synthase subunit alpha, chloroplastic	-1.29	-2.19
ATPB	P17614	ATP synthase subunit beta, mitochondrial	-1.86	-2.00
Atp5f1b	P56480	ATP synthase subunit beta, mitochondrial	-1.57	-1.81
MT-CYB	P00156	Cytochrome b	6.66	6.83
Uqcrc1	Q9CZ13	Cytochrome b-c1 complex subunit 1, mitochondrial	-1.87	-2.27
petB	Q9BBQ6	Cytochrome b6	2.77	3.89
petC1	P30361	Cytochrome b6-f complex iron-sulfur subunit 1, chloroplastic	-2.09	-2.49
At5g35735At5g35735	Q9FKH6	Cytochrome b561 and DOMON domain-containing protein At5g35735	1.27	2.20
Mtco1	P00397	Cytochrome c oxidase subunit 1	-1.42	-1.82
MT-CO1	P00395	Cytochrome c oxidase subunit 1	9.35	9.69
COI	P50668	Cytochrome c oxidase subunit 1	-12.72	-12.72
MT-CO2	P00403	Cytochrome c oxidase subunit 2	8.00	8.52
Mtco2	P00405	Cytochrome c oxidase subunit 2	-1.24	-1.62
MT-CO3	P00414	Cytochrome c oxidase subunit 3	8.50	8.71
mt-Co3	P00416	Cytochrome c oxidase subunit 3	-1.17	-1.54
COX4I1	P13073	Cytochrome c oxidase subunit 4 isoform 1, mitochondrial	9.07	8.96
Cox4i1	P19783	Cytochrome c oxidase subunit 4 isoform 1, mitochondrial	-1.23	-1.87
Cox6a1	P43024	Cytochrome c oxidase subunit 6A1, mitochondrial	-2.53	-2.57
COX6B-1	Q9S7L9	Cytochrome c oxidase subunit 6b-1	9.66	11.54
Cox8a	Q64445	Cytochrome c oxidase subunit 8A, mitochondrial	-1.68	-1.78
Cox8b	P48772	Cytochrome c oxidase subunit 8B, mitochondrial	-3.29	-3.81
Cyc1	Q9D0M3	Cytochrome c1, heme protein, mitochondrial	-1.26	-1.89
petA	A0A348	Cytochrome f	-2.61	-2.13
Ndufa10	Q99LC3	NADH dehydrogenase [ubiquinone] 1 alpha subcomplex subunit 10, mitochondrial	-2.17	-2.57
Ndufv1	Q91YT0	NADH dehydrogenase [ubiquinone] flavoprotein 1, mitochondrial	-1.57	-2.26
At5g08530	Q9FNN5	NADH dehydrogenase [ubiquinone] flavoprotein 1, mitochondrial	-2.56	-1.66
FRO1	Q9FJW4	NADH dehydrogenase [ubiquinone] iron-sulfur protein 4, mitochondrial	2.97	4.07
ndhF	Q06R83	NAD(P)H-quinone oxidoreductase subunit 5, chloroplastic	2.12	3.66
ndhL	Q9CAC5	NAD(P)H-quinone oxidoreductase subunit L, chloroplastic	-2.26	-3.04
ndhN	Q9LVM2	NAD(P)H-quinone oxidoreductase subunit N, chloroplastic	-2.60	-2.72
MT-ND1	P03886	NADH-ubiquinone oxidoreductase chain 1	7.45	7.40
Mtnd2	P03893	NADH-ubiquinone oxidoreductase chain 2	-1.12	-1.56
MT-ND2	P03891	NADH-ubiquinone oxidoreductase chain 2	11.98	11.89
MT-ND3	P03897	NADH-ubiquinone oxidoreductase chain 3	10.93	10.42
MT-ND4	P03905	NADH-ubiquinone oxidoreductase chain 4	8.89	9.07
Mtnd4	P03911	NADH-ubiquinone oxidoreductase chain 4	-1.11	-1.49
Mtnd5	P03921	NADH-ubiquinone oxidoreductase chain 5	-1.31	-1.85
MT-ND5	P03915	NADH-ubiquinone oxidoreductase chain 5	8.00	8.56
NCPR	P37116	NADPH--cytochrome P450 reductase	-1.88	-2.45
PGRL1A	Q8H112	PGR5-like protein 1A, chloroplastic	-2.35	-2.09

At5g40382	Q9FNE0	Putative cytochrome c oxidase subunit 5C-4	6.13	7.72
At4g36750	O23207	Probable NAD(P)H dehydrogenase (quinone) FQR1-like 2	-1.54	-1.80
SOX	Q9S850	Sulfite oxidase	-2.92	-2.09

Table S13. Seventy-two genes associated with secondary metabolism at 5 and 25% PEG vs. CK.

Gene name	SwissProt ID	Protein name	log_2FC (5% PEG vs. CK)	log_2FC (25% PEG vs. CK)
**Gibberellin biosynthetic (3)**				
GA2OX1	Q9SQ80	Gibberellin 2-beta-dioxygenase 1	1.75	2.44
GA2OX6	Q7XP65	Gibberellin 2-beta-dioxygenase 6	3.80	4.35
LE	O24648	Gibberellin 3-beta-dioxygenase 1	4.84	3.31
**Cytokinin biosynthesis (5)**				
IPT3	Q93WC9	Adenylate isopentenyltransferase 3, chloroplastic	-1.19	-3.96
IPT5	Q94ID2	Adenylate isopentenyltransferase 5, chloroplastic	2.40	2.81
CKX1	O22213	Cytokinin dehydrogenase 1	2.08	3.50
LOG1	Q8RUN2	Cytokinin riboside 5'-monophosphate phosphoribohydrolase LOG1	-1.07	-3.97
LOG4	Q9LFH3	Cytokinin riboside 5'-monophosphate phosphoribohydrolase LOG4	1.76	3.05
**Auxin biosynthesis (3)**				
YUC5	Q9LKC0	Probable indole-3-pyruvate monooxygenase YUCCA5	-3.44	-3.72
YUC10	Q9FVQ0	Probable indole-3-pyruvate monooxygenase YUCCA10	1.32	3.26
CYP725A2	Q8W4T9	Taxane 13-alpha-hydroxylase	-1.09	-1.95
**Ethylene biosynthetic process (5)**				
DK-ACO1	Q8S932	1-aminocyclopropane-1-carboxylate oxidase	1.71	3.05
ACO	P31237	1-aminocyclopropane-1-carboxylate oxidase	-2.59	-1.96
ACS1	Q07262	1-aminocyclopropane-1-carboxylate synthase	4.85	5.30
ACS-1	P37821	1-aminocyclopropane-1-carboxylate synthase	-1.69	-2.29
ACS3	Q42881	1-aminocyclopropane-1-carboxylate synthase 3	-3.53	-2.60
**Abscisic acid metabolism (3)**				
NCED1	O24023	9-cis-epoxycarotenoid dioxygenase NCED1, chloroplastic	7.50	8.69
NCED2	K4CJJ1	9-cis-epoxycarotenoid dioxygenase NCED2, chloroplastic	1.61	2.72
CYP707A1	Q949P1	Abscisic acid 8'-hydroxylase 1	2.49	2.60
**Carotenoid metabolism (3)**				
PSY1	P37272	Bifunctional 15-cis-phytoene synthase, chromoplastic	-2.90	-2.62
GGPPS1	P34802	Heterodimeric geranylgeranyl pyrophosphate synthase large subunit 1, chloroplastic	-1.79	-2.86
CCD4	O49675	Probable carotenoid cleavage dioxygenase 4, chloroplastic	-2.48	-2.18
**Terpenoid biosynthetic (4)**				
ATESY	Q6PWU2	(-)-alpha-terpineol synthase	1.17	2.03
CYC2	A0A1C9CX66	(S)-8-oxocitronellyl enol synthase CYC2	4.80	6.22
CYP76B10	D1MI46	Geraniol 8-hydroxylase	1.07	1.86
OSCBPW	Q8W3Z2	Lupeol synthase	-2.20	-1.94
**Lignin biosynthetic (4)**				
COMT1	Q8W013	Caffeic acid 3-O-methyltransferase	-2.61	-2.39
CSE	Q9C942	Caffeoylshikimate esterase	2.48	2.46
SILDF	Q94KL7	Secoisolariciresinol dehydrogenase	1.29	0.41
SILDP	Q94KL8	Secoisolariciresinol dehydrogenase	4.32	5.69
**Flavonoid biosynthetic (18)**				
UGT85A23	F8WLS6	7-deoxyloganetin glucosyltransferase	0.80	0.75
UGT85A24	F8WKW1	7-deoxyloganetin glucosyltransferase	-2.37	-1.99
UGT94E5	F8WKW8	Beta-D-glucosyl crocetin beta-1,6-glucosyltransferase	5.39	7.00
CYP75A2	P37120	Flavonoid 3',5'-hydroxylase	3.04	6.80
CYP75B2	Q9SBQ9	Flavonoid 3'-monooxygenase	1.91	2.91
CYP75B3	Q7G602	Flavonoid 3'-monooxygenase CYP75B3	5.61	7.22
UGT74F1	O22820	Flavonol 7-O-beta-glucosyltransferase UGT74F1	1.21	2.44
JOX1	Q9SRM3	Jasmonate-induced oxygenase 1	-1.50	-3.38
ANR	Q5XLY0	Putative anthocyanidin reductase	1.35	1.91
UGD2	Q9LIA8	UDP-glucose 6-dehydrogenase 2	-2.28	-2.07
UGT76A2	U3UA11	UDP-glucose iridoid glucosyltransferase	-1.52	-2.00
GT6	Q2V6K0	UDP-glucose flavonoid 3-O-glucosyltransferase 6	2.18	3.42
GT7	Q2V6J9	UDP-glucose flavonoid 3-O-glucosyltransferase 7	1.33	1.95
UGT9	W8JMV4	UDP glycosyltransferase 9	-3.36	-2.60
UGT73C3	Q9ZQ96	UDP-glycosyltransferase 73C3	1.55	1.86
UGT74F2	O22822	UDP-glycosyltransferase 74F2	1.86	2.83
UGT76E3	Q494Q1	UDP-glycosyltransferase 76E3	1.54	2.20

UGT92A1	Q9LXV0	UDP-glycosyltransferase 92A1	1.71	1.64
**Alkaloid biosynthesis (3)**				
LAMT	B2KPR3	Loganic acid O-methyltransferase	-1.05	-2.85
CYP80B2	Q9FXW4	Probable (S)-N-methylcoclaurine 3'-hydroxylase isozyme 2	-2.31	-2.02
BBE1	P93479	Reticuline oxidase	1.43	2.56
**Phenylalanine catabolism (6)**				
HPD	P93836	4-hydroxyphenylpyruvate dioxygenase	1.56	2.70
CAD1	Q2KNL5	Cinnamyl alcohol dehydrogenase 1	-0.95	-0.13
CYP73A4	P48522	Trans-cinnamate 4-monooxygenase	-2.70	-2.04
CYP73A16	Q43054	Trans-cinnamate 4-monooxygenase	1.43	2.03
PAL	P45730	Phenylalanine ammonia-lyase	1.34	1.05
PAL3	P45733	Phenylalanine ammonia-lyase	-2.36	-2.17
**Ubiquinone biosynthetic (3)**				
MENG	Q3ED65	2-phytyl-1,4-beta-naphthoquinone methyltransferase, chloroplastic	-1.00	-3.21
Coq8a	Q60936	Atypical kinase COQ8A, mitochondrial	-1.02	-1.42
D4H	O04847	Deacetoxyvindoline 4-hydroxylase	1.59	2.80
**Other (12)**				
10HGO	Q6V4H0	8-hydroxygeraniol dehydrogenase	1.71	2.46
DFRA	P51107	Dihydroflavonol 4-reductase	-1.99	-2.47
DIR11	Q67YM6	Dirigent protein 11	2.94	4.20
DIR20	Q9C891	Dirigent protein 20	-1.01	-1.54
GGP3	Q9M0A5	Gamma-glutamyl peptidase 3	1.94	-1.12
GGP5	O82225	Gamma-glutamyl peptidase 5	-2.14	-2.22
MIOX5	Q9FJU4	Inositol oxygenase 5	-1.24	-3.45
AMAT	Q3ZPN4	Methanol O-anthraniloyltransferase	1.43	1.15
BETV6	Q9FUW6	Phenylcoumaran benzylic ether reductase Betv6	-2.20	-2.08
TKT2	O78328	Probable 1-deoxy-D-xylulose-5-phosphate synthase, chloroplastic	-1.88	-1.82
AOP1.2	Q945B6	Probable 2-oxoglutarate-dependent dioxygenase AOP1.2	-1.08	1.24
THI1-2	F6H7K5	Thiamine thiazole synthase 2, chloroplastic	-2.47	-2.28

Table S14. One hundred and seventy-eight genes associated with transport at 5 and 25% PEG vs. CK.

Gene name	SwissProt ID	Protein name	log_2FC (5% PEG vs. CK)	log_2FC (25% PEG vs. CK)
**ABC (12)**				
ABCB1	Q9ZR72	ABC transporter B family member 1	-2.62	-1.87
ABCC1	Q9C8G9	ABC transporter C family member 1	-1.04	-1.47
ABCF1	Q9FJH6	ABC transporter F family member 1	-2.13	-0.27
ABCC2	Q42093	ABC transporter C family member 2	-3.13	-1.97
ABCB4	O80725	ABC transporter B family member 4	1.10	1.97
ABCF5	Q9LV93	ABC transporter F family member 5	-1.51	-1.40
ABCC12	Q9C8H0	ABC transporter C family member 12	-1.08	-1.47
ABCB12	Q9FWX8	ABC transporter B family member 12	1.08	2.02
ABCI17	Q9C9W0	ABC transporter I family member 17	1.04	1.73
ABCG21	Q7XA72	ABC transporter G family member 21	1.46	2.80
ABCG24	Q9MAG3	ABC transporter G family member 24	-1.12	-1.72
ABCG36	Q9XIE2	ABC transporter G family member 36	-1.60	-2.35
**Amino acid transport (8)**				
AAP6	P92934	Amino acid permease 6	1.31	1.89
AVT1A	O80668	Amino acid transporter AVT1A	1.29	-0.28
AVT1I	F4J1Q9	Amino acid transporter AVT1I	2.13	3.14
AVT1J	Q9LXF8	Amino acid transporter AVT1J	2.40	4.00
AVT3C	Q9SVG0	Amino acid transporter AVT3C	0.16	0.31
AVT6A	Q9LI61	Amino acid transporter AVT6A	-2.48	-1.65
AVT6C	Q9LYM2	Amino acid transporter AVT6C	-1.10	-2.92
CAT1	Q84MA5	Cationic amino acid transporter 1	-1.54	-2.46
**Transmembrane transport (11)**				
ANT	P25083	ADP,ATP carrier protein, mitochondrial	-3.05	-2.24
AAC3	O49447	ADP,ATP carrier protein 3, mitochondrial	-4.06	-2.02
SLC25A6	P12236	ADP/ATP translocase 3	8.42	8.91
cemA	Q09G34	Chloroplast envelope membrane protein	3.12	4.43
TMN3	Q9ZPS7	Transmembrane 9 superfamily member 3	-2.67	-2.26
TMN7	Q9LIC2	Transmembrane 9 superfamily member 7	-2.15	-1.74
TMEM230	Q5ZLH4	Transmembrane protein 230	1.09	2.23
TPT	P21727	Triose phosphate/phosphate translocator, chloroplastic	-2.30	-2.37
At2g37460	Q9ZUS1	WAT1-related protein At2g37460	-2.56	-1.94

At5g07050	Q9FL41	WAT1-related protein At5g07050	1.94	2.59
At3g28050	Q94JU2	WAT1-related protein At3g28050	1.06	2.09
**Protein transport (26)**				
ARF1	P51821	ADP-ribosylation factor 1	-6.02	-10.75
AGD5	Q9FL69	ADP-ribosylation factor GTPase-activating protein AGD5	1.06	-0.14
AP2M1	Q4R706	AP-2 complex subunit mu	8.66	8.26
ATG8C	A2YS06	Autophagy-related protein 8C	1.28	2.36
ATG8F	Q8VYK7	Autophagy-related protein 8f	1.48	1.97
At4g14600	Q8VXX9	Bet1-like protein At4g14600	1.23	2.00
BETAC-AD	O81742	Beta-adaptin-like protein C	-2.62	-2.08
Os11g0104900	Q2RBN7	Clathrin heavy chain 1	-1.99	-2.16
CHC1	Q0WNJ6	Clathrin heavy chain 1	-2.95	-2.79
EPSIN2	Q67YI9	Clathrin interactor EPSIN 2	-1.83	-2.48
RAN	Q4R4M9	GTP-binding nuclear protein Ran	9.31	8.28
RAN	P38548	GTP-binding nuclear protein Ran/TC4	-3.87	-3.06
RAN1	P38546	GTP-binding nuclear protein Ran1	-2.38	-2.26
SecA	Q41062	Protein translocase subunit SecA, chloroplastic	-2.55	-2.47
RAB11D	Q40194	Ras-related protein Rab11D	1.17	2.20
RAB11E	Q40195	Ras-related protein Rab11E	1.07	2.21
RABA6B	Q0WQN4	Ras-related protein RABA6b	1.69	2.76
RABC1	O23657	Ras-related protein RABC1	2.25	3.89
RABG3F	Q9LS94	Ras-related protein RABG3f	-2.51	-2.46
VIT1	Q9ZUA5	Vacuolar iron transporter 1	-1.05	-0.22
VPS2.1	Q9SKI2	Vacuolar protein sorting-associated protein 2 homolog 1	-4.71	-2.04
VPS32.1	O82197	Vacuolar protein sorting-associated protein 32 homolog 1	-2.47	-2.23
BP80	P93484	Vacuolar-sorting receptor 1	-2.22	-2.31
VAMP725	O48850	Vesicle-associated membrane protein 725	-2.83	-2.24
PVA22	B9DHD7	Vesicle-associated protein 2-2	1.24	2.01
PVA41	Q1ECE0	Vesicle-associated protein 4-1	1.00	1.59
**Lipid transport (9)**				
Fabp4	P04117	Fatty acid-binding protein, adipocyte	-3.14	-3.70
NLTP	Q39794	Non-specific lipid-transfer protein	1.03	2.33
NLTPH	Q39950	Non-specific lipid-transfer protein	1.77	2.62
NLTP1	Q42762	Non-specific lipid-transfer protein	5.11	6.20
LTP1	O24037	Non-specific lipid-transfer protein 1	-1.85	-1.91
NLTP3	Q43019	Non-specific lipid-transfer protein 3	1.87	2.30
At4g12500	Q9SU33	pEARLI1-like lipid transfer protein 3	3.88	4.59
SYT3	Q7XA06	Synaptotagmin-3	2.01	3.15
SYT4	A0JJX5	Synaptotagmin-4	-2.30	-1.96
**Sugar transport (4)**				
SWEET15	Q9FY94	Bidirectional sugar transporter SWEET15	4.97	6.81
STP1	P23586	Sugar transport protein 1	-1.01	-2.43
STP13	Q94AZ2	Sugar transport protein 13	2.06	3.17
SUT	Q03411	Sucrose transport protein	-2.09	-2.27
**Others (108)**				
AZG1	Q9SRK7	Adenine/guanine permease AZG1	-1.14	2.48
AZG2	Q84MA8	Adenine/guanine permease AZG2	3.52	5.88
ACTN1	P12814	Alpha-actinin-1	8.44	7.78
ACTN4	O43707	Alpha-actinin-4	8.88	8.62
Mtatp6	P00848	ATP synthase subunit a	-1.20	-1.58
ATP5F1A	A5A6H5	ATP synthase subunit alpha, mitochondrial	8.92	8.88
ATPF2	P31853	ATP synthase subunit b', chloroplastic	-2.07	-2.24
ATP5F1C	P05631	ATP synthase subunit gamma, mitochondrial	-9.21	-9.21
CSC1	Q5XEZ5	Calcium permeable stress-gated cation channel 1	-2.90	-1.90
At5g42610	Q9FJV7	Calcium uniporter protein 4, mitochondrial	2.79	3.91
ACA4	O22218	Calcium-transporting ATPase 4, plasma membrane-type	-3.05	-2.11
ACA5	Q7X8B5	Calcium-transporting ATPase 5, plasma membrane-type	-2.87	-1.74
POPTRDRAFT_834139	B9I0G0	CASP-like protein 2A1	1.23	2.43
GSVIVT00013502001	A7R385	CASP-like protein 2B1	1.37	1.82
CCX2	Q9FKP2	Cation/calcium exchanger 2	1.45	2.55
CHX23	Q8VYD4	Cation/H(+) antiporter 23, chloroplastic	1.74	2.97
CHX24	Q1HDT2	Cation/H(+) antiporter 24	2.01	2.89

CD63	P08962	CD63 antigen	9.58	9.57
CD81	P60033	CD81 antigen	8.21	8.68
CLIC1	O00299	Chloride intracellular channel protein 1	9.15	8.71
At1g62020	Q94A40	Coatomer subunit alpha-1	-2.16	-3.06
zgc:73324	Q6PBN4	Coenzyme Q-binding protein COQ10 homolog, mitochondrial	2.30	3.55
ATX1	Q94BT9	Copper transport protein ATX1	-0.22	0.31
COPT2	Q9STG2	Copper transporter 2	-1.13	-3.62
RAN1	Q9S7J8	Copper-transporting ATPase RAN1	-2.08	-2.85
CNGC18	Q9LEQ3	Cyclic nucleotide-gated ion channel 18	1.69	2.42
CHMP1A	Q8LE58	ESCRT-related protein CHMP1A	-4.50	-2.98
AP1	Q9ZTS2	Ferredoxin, chloroplastic	-1.94	-2.35
FRO2	P92949	Ferric reduction oxidase 2	-2.58	-1.65
FRO6	Q8RWS6	Ferric reduction oxidase 6	-2.06	-2.70
FTH1	P02794	Ferritin heavy chain	12.46	12.13
FTL	P02792	Ferritin light chain	5.80	6.64
FRI	Q94FY2	Ferritin, chloroplastic	-2.40	-1.92
FER2	Q8H1T3	Ferritin-2, chloroplastic	-2.38	-2.05
HIPP33	F4JZL7	Heavy metal-associated isoprenylated plant protein 33	-1.21	-1.69
NRT2.7	Q9LYK2	High affinity nitrate transporter 2.7	1.86	2.55
MFSD14A	Q96MC6	Hippocampus abundant transcript 1 protein	1.44	1.54
SUVH4	Q8GZB6	Histone-lysine N-methyltransferase, H3 lysine-9 specific SUVH4	1.71	2.61
IMPA1	Q96321	Importin subunit alpha-1	-2.58	-2.49
PHT1-4	Q96303	Inorganic phosphate transporter 1-4	1.73	2.67
INT1	Q8VZR6	Inositol transporter 1	-2.11	-2.40
ISU1	Q8LR34	Iron-sulfur cluster assembly protein 1	-0.59	-0.25
LAPTM4A	Q5RAH0	Lysosomal-associated transmembrane protein 4A	8.81	8.87
MSL2	Q56X46	Mechanosensitive ion channel protein 2, chloroplastic	1.18	2.00
MTP10	Q0WU02	Metal tolerance protein 10	0.63	4.56
MT4B	Q42377	Metallothionein-like protein 4B	7.34	9.20
YSL3	Q2EF88	Metal-nicotianamine transporter YSL3	-2.05	-2.20
BAC2	Q9CA93	Mitochondrial arginine transporter BAC2	1.35	2.36
Ucp1	P12242	Mitochondrial brown fat uncoupling protein 1	-12.42	-12.42
Slc25a20	Q9Z2Z6	Mitochondrial carnitine/acylcarnitine carrier protein	-1.77	-2.21
VDAC2	P42056	Mitochondrial outer membrane protein porin of 36 kDa	-3.31	-2.60
SFC1	Q9M038	Mitochondrial succinate-fumarate transporter 1	-1.06	-2.42
Slc25a19	Q9DAM5	Mitochondrial thiamine pyrophosphate carrier	-4.53	-3.66
MFSD5	Q0VC03	Molybdate-anion transporter	-3.41	-2.55
Mb	P04247	Myoglobin	2.40	1.86
OCT3	Q9SA38	Organic cation/carnitine transporter 3	-4.76	-7.90
OEP162	Q0WMZ5	Outer envelope pore protein 16-2, chloroplastic	2.34	4.14
ORP1C	Q8L751	Oxysterol-binding protein-related protein 1C	1.21	1.98
ORP4B	Q9SW00	Oxysterol-binding protein-related protein 4B	2.58	3.49
PNC1	B6ZJZ9	Peroxisomal adenine nucleotide carrier 1	2.85	2.64
SLC25A3	Q5R7W2	Phosphate carrier protein, mitochondrial	9.31	8.62
PHO1-H9	Q9LJW0	Phosphate transporter PHO1 homolog 9	-1.54	-2.59
PDR17	P53844	Phosphatidylinositol transfer protein PDR17	-2.41	-3.69
SFH8	F4IHJ0	Phosphatidylinositol/phosphatidylcholine transfer protein SFH8	-2.23	-2.26
PPT2	Q8H0T6	Phosphoenolpyruvate/phosphate translocator 2, chloroplastic	-1.84	-5.43
Os04g0656100	Q7XPY2	Plasma membrane ATPase	3.26	2.32
PMA1	Q08435	Plasma membrane ATPase 1	-2.89	-2.52
PMA3	Q08436	Plasma membrane ATPase 3	1.89	3.13
PMA4	Q03194	Plasma membrane ATPase 4	-2.26	-1.70
PLT5	Q8VZ80	Polyol transporter 5	-1.04	-2.71
PRA1F2	Q9C889	PRA1 family protein F2	1.18	2.50
At5g56450	Q9FM86	Probable ADP,ATP carrier protein At5g56450	1.20	2.34
PIP1-5	Q8LAA6	Probable aquaporin PIP1-5	-3.69	-2.31
TIP1-1	P50156	Probable aquaporin TIP1-1	-2.36	-2.90
TIP3-2	O22588	Probable aquaporin TIP3-2	6.04	7.71
CNGC5	Q8RWS9	Probable cyclic nucleotide-gated ion channel 5	-3.57	-2.64

At1g67300	Q9FYG3	Probable plastidic glucose transporter 2	1.39	2.55
BASS2	Q5VRB2	Probable sodium/metabolite cotransporter BASS2, chloroplastic	-1.19	-1.98
SULTR3;4	Q9LW86	Probable sulfate transporter 3.4	1.83	2.76
PROT1	P92961	Proline transporter 1	-1.42	-3.44
DTX51	Q9SZE2	Protein DETOXIFICATION 51	-1.85	-3.27
ELC	Q9LHG8	Protein ELC	3.58	5.66
NPF5.1	Q8VZR7	Protein NRT1/ PTR FAMILY 5.1	-1.04	-2.29
NPF5.8	Q9LFR1	Protein NRT1/ PTR FAMILY 5.8	2.19	3.37
NPF7.3	Q9LQL2	Protein NRT1/ PTR FAMILY 7.3	-2.44	-0.83
NFD4	F4I9E1	Protein NUCLEAR FUSION DEFECTIVE 4	-1.01	-1.79
TOC75-3	Q9STE8	Protein TOC75-3, chloroplastic	-3.05	-2.05
Slc36a2	Q8BHK3	Proton-coupled amino acid transporter 2	-6.61	-4.81
ACA13	Q9LIK7	Putative calcium-transporting ATPase 13, plasma membrane-type	1.30	1.43
AVP	P21616	Pyrophosphate-energized vacuolar membrane proton pump	-2.39	-2.39
REMO	P93788	Remorin	-2.41	-2.31
Atp2a2	O55143	Sarcoplasmic/endoplasmic reticulum calcium ATPase 2	2.00	1.61
NCL	Q8L636	Sodium/calcium exchanger NCL	-1.99	-3.49
NHX4	Q8S397	Sodium/hydrogen exchanger 4	2.24	3.70
Atp1a2	P06686	Sodium/potassium-transporting ATPase subunit alpha-2	-1.52	-1.47
SLAH3	Q9FLV9	S-type anion channel SLAH3	-1.51	-1.66
SULTR3;1	Q9SV13	Sulfate transporter 3.1	2.91	1.36
STXBP5	Q5T5C0	Syntaxin-binding protein 5	1.53	2.33
At3g15360	Q9SEU6	Thioredoxin M4, chloroplastic	-2.00	-1.96
TDT	Q8LG88	Tonoplast dicarboxylate transporter	1.05	2.21
TOC132	Q9SLF3	Translocase of chloroplast 132, chloroplastic	-3.00	-2.13
UTR4	Q9LDX3	UDP-galactose/UDP-glucose transporter 4	1.12	2.23
VATL	Q96473	V-type proton ATPase 16 kDa proteolipid subunit	-2.74	-1.88
CVA69.24	P31405	V-type proton ATPase catalytic subunit A	-2.51	-2.10
VHA-a2	Q9SJT7	V-type proton ATPase subunit a2	-2.13	-2.87
VHA-D	Q9XGM1	V-type proton ATPase subunit D	-3.58	-3.58
VATE	Q9SWE7	V-type proton ATPase subunit E	-2.67	-3.24
ZIP11	Q94EG9	Zinc transporter 11	-2.72	-2.03

Table S15. Sixty-five genes associated with polynucleotide metabolism at 5 and 25% PEG vs. CK.

**Gene name**	**SwissProt ID**	**Protein name**	**$\log_2$FC (5% PEG vs. CK)**	**$\log_2$FC (25% PEG vs. CK)**
ROC1	P49313	30 kDa ribonucleoprotein, chloroplastic	-2.49	-2.17
APT1	Q43199	Adenine phosphoribosyltransferase 1	-2.81	-2.63
CSP41A	Q9LYA9	Chloroplast stem-loop binding protein of 41 kDa a, chloroplastic	-2.08	-2.07
GIP	P04146	Copia protein	4.37	2.46
CDA1	O65896	Cytidine deaminase 1	1.13	1.86
RH7	Q39189	DEAD-box ATP-dependent RNA helicase 7	-2.34	-2.34
MSH6	O04716	DNA mismatch repair protein MSH6	-1.13	-1.80
REV7	Q94FL5	DNA polymerase zeta processivity subunit	1.26	1.06
At3g03300	Q3EBC8	Endoribonuclease Dicer homolog 2	1.21	1.58
ORF V	P03554	Enzymatic polyprotein	3.33	4.66
RBG7	Q03250	Glycine-rich RNA-binding protein 7	-2.28	-1.90
Gmpr	Q9DCZ1	GMP reductase 1	-2.50	-2.64
GSDA	Q94BU8	Guanosine deaminase	1.13	2.19
RNP1	Q8W034	Heterogeneous nuclear ribonucleoprotein 1	-2.71	-1.26
Hnrnpa1	P49312	Heterogeneous nuclear ribonucleoprotein A1	9.42	9.33
H1	P40267	Histone H1	-2.20	-2.45
At2g30620	P26569	Histone H1.2	-1.96	-2.01
OsI_011536	A2XHJ3	Histone H3.3	-1.99	-1.82
HMGL	P26585	HMG1/2-like protein	-3.13	-2.51
HOP2	Q9FX64	Homologous-pairing protein 2 homolog	1.96	2.95
LARP6A	Q94A38	La-related protein 6A	-2.08	-2.62
LARP6B	O80567	La-related protein 6B	-2.30	-1.98
MORF5	Q9C7Y2	Multiple organellar RNA editing factor 5, chloroplastic/mitochondrial	-3.14	-2.69

NSE4A	Q9C689	Non-structural maintenance of chromosomes element 4 homolog A	1.45	2.32
PAPS2	O82312	Nuclear poly(A) polymerase 2	-4.18	-2.48
NTF2	Q9FME2	Nuclear transport factor 2	-2.34	-3.78
NDK1	Q56E62	Nucleoside diphosphate kinase 1	-2.18	-2.02
NME2	P22392	Nucleoside diphosphate kinase B	9.22	9.40
Os04g0620700	Q7XTT4	Nucleolin 2	-2.20	-2.28
UBP1	Q9M427	Oligouridylate-binding protein 1	-2.52	-3.00
UBP1B	Q9LQI9	Oligouridylate-binding protein 1B	-2.35	-2.49
PCMP-E22	Q1PFA6	Pentatricopeptide repeat-containing protein At2g02750	1.01	1.92
PCMP-H44	Q9SI53	Pentatricopeptide repeat-containing protein At2g03880, mitochondrial	1.21	1.67
PCMP-H41	Q9SHZ8	Pentatricopeptide repeat-containing protein At2g22070	-2.59	-3.49
PABPC1	P11940	Polyadenylate-binding protein 1	3.78	3.83
PABN2	Q9FJN9	Polyadenylate-binding protein 2	-2.41	-2.43
PABN3	Q9LX90	Polyadenylate-binding protein 3	-4.62	-3.53
RBP45	Q9LEB4	Polyadenylate-binding protein RBP45	-2.34	-2.31
RBP47	Q9LEB3	Polyadenylate-binding protein RBP47	-2.20	-2.23
CID7	O64843	Polyadenylate-binding protein-interacting protein 7	-2.42	-2.60
CFIS1	Q94AF0	Pre-mRNA cleavage factor Im 25 kDa subunit 1	1.39	2.08
PRP8A	Q9SSD2	Pre-mRNA-processing-splicing factor 8A	-2.99	-2.52
AAP7	Q9FF99	Probable amino acid permease 7	1.97	3.22
At1g14650	Q8RXF1	Probable splicing factor 3A subunit 1	-1.87	-2.36
DCP5	Q9C658	Protein decapping 5	-2.08	-2.37
ML2	Q6ZI17	Protein MEI2-like 2	1.33	2.35
ML4	Q64M78	Protein MEI2-like 4	-2.01	-1.85
ORF 3	Q89703	Putative enzymatic polyprotein	2.07	2.44
PCMP-H22	Q9CAA8	Putative pentatricopeptide repeat-containing protein At1g68930	-1.17	-1.46
pol3	P04323	Retrovirus-related Pol polyprotein from transposon 17.6	-1.89	0.16
pol2	P20825	Retrovirus-related Pol polyprotein from transposon 297	2.42	3.80
RE2	Q9ZT94	Retrovirus-related Pol polyprotein from transposon RE2	0.22	0.02
rnhA	Q9KEI9	Ribonuclease H	2.25	3.12
SIGC	O24621	RNA polymerase sigma factor sigC	-2.28	-3.73
RBM39	Q5RC80	RNA-binding protein 39	-2.78	-2.04
RDR1	Q9LQV2	RNA-dependent RNA polymerase 1	2.40	1.97
RS41	P92966	Serine/arginine-rich splicing factor RS41	-3.09	-2.71
U2AF65A	Q9ZR39	Splicing factor U2af large subunit A	-2.35	-2.11
U2AF35A	Q9ZQW8	Splicing factor U2af small subunit A	1.38	2.15
trmB	Q2JJQ0	tRNA (guanine-N(7)-)-methyltransferase	2.40	2.88
RNU1	Q42404	U1 small nuclear ribonucleoprotein 70 kDa	-2.98	-2.49
RAD23C	Q84L31	Ubiquitin receptor RAD23c	-2.29	-4.29
UKL1	Q9FKS0	Uridine kinase-like protein 1, chloroplastic	-4.67	-4.45
Uck1	P52623	Uridine-cytidine kinase 1	-3.09	-2.79
ECT2	Q9LJE5	YTH domain-containing protein ECT2	-2.26	-2.41

Table S16. Two hundred and twenty-eight genes associated with translation at 5 and 25% PEG vs. CK.

Gene name	SwissProt ID	Protein name	log_2FC (5% PEG vs. CK)	log_2FC (25% PEG vs. CK)
RPN9A	Q8RWF0	26S proteasome non-ATPase regulatory subunit 13 homolog A	-2.72	-3.25
Psmd2	Q4FZT9	26S proteasome non-ATPase regulatory subunit 2	8.04	8.32
RPN1A	Q9SIV2	26S proteasome non-ATPase regulatory subunit 2 homolog A	-2.13	-2.41
RPT2A	Q9SZD4	26S proteasome regulatory subunit 4 homolog A	-3.20	-2.54
PRS6B	P54778	26S proteasome regulatory subunit 6B homolog	-3.70	-2.40
rps5	Q9ST69	30S ribosomal protein S5, chloroplastic	-2.17	-3.05
PRPS9	P82278	30S ribosomal protein S9, chloroplastic	-1.73	-1.99
rps10	O77082	40S ribosomal protein S10	-10.61	-10.61
RPS10	P46783	40S ribosomal protein S10	5.05	4.40
RPS10A	Q9SW09	40S ribosomal protein S10-1	-2.22	-2.92
Rps11	P62282	40S ribosomal protein S11	10.42	10.48
RPS12	Q76I81	40S ribosomal protein S12	9.83	10.02
Rps14	P62264	40S ribosomal protein S14	10.37	10.36
RPS15	P62842	40S ribosomal protein S15	9.71	9.96
RPS15A	Q9AT34	40S ribosomal protein S15a	-2.68	-2.54

RPS16	P46293	40S ribosomal protein S16	-3.50	-5.20
Rps16	P14131	40S ribosomal protein S16	9.72	9.89
RPS17	P08708	40S ribosomal protein S17	10.08	10.12
RPS18	P62272	40S ribosomal protein S18	10.60	10.79
RPS19	Q32PD5	40S ribosomal protein S19	9.59	10.39
RPS19C	Q9FNP8	40S ribosomal protein S19-3	-2.16	-2.82
RPS2	P15880	40S ribosomal protein S2	11.23	11.69
RPS20	P60866	40S ribosomal protein S20	9.58	9.48
RPS2B	Q93VB8	40S ribosomal protein S2-2	-2.54	-2.60
RPS23	P62266	40S ribosomal protein S23	9.74	9.20
Rps24	P62850	40S ribosomal protein S24	9.58	9.69
RPS24A	Q9SS17	40S ribosomal protein S24-1	-0.67	-1.04
RPS26C	Q9LYK9	40S ribosomal protein S26-3	-2.91	-2.91
RPS27	Q96564	40S ribosomal protein S27	1.32	1.57
RPS27L	Q3T0B7	40S ribosomal protein S27-like	-10.42	-10.42
RPS3	P23396	40S ribosomal protein S3	10.09	10.45
RPS3A	Q56JV9	40S ribosomal protein S3a	10.27	10.45
RpS4	Q4PMB3	40S ribosomal protein S4	-11.40	-11.40
RPS4X	Q76N24	40S ribosomal protein S4, X isoform	10.29	10.41
RPS4D	Q8VYK6	40S ribosomal protein S4-3	-2.02	-1.90
RPS5	Q5E988	40S ribosomal protein S5	9.65	9.70
Rps6	P62755	40S ribosomal protein S6	10.75	10.63
RPS6A	O48549	40S ribosomal protein S6-1	-2.20	-2.28
RPS7	A6H769	40S ribosomal protein S7	9.34	1.41
RPS8	Q4R6P8	40S ribosomal protein S8	10.22	10.30
RPS9	A9L913	40S ribosomal protein S9	9.95	10.00
179B	O80377	40S ribosomal protein SA	-2.13	-2.05
RPSA	P08865	40S ribosomal protein SA	10.49	10.54
PSRP6	Q9FKP0	50S ribosomal protein 6, chloroplastic	-2.49	-2.20
RPL1	Q9LY66	50S ribosomal protein L1, chloroplastic	-2.67	-2.61
RPL10	O80362	50S ribosomal protein L10, chloroplastic	-2.35	-2.08
RPL13	Q9SYL9	50S ribosomal protein L13, chloroplastic	-2.69	-2.24
RPL15	P25873	50S ribosomal protein L15, chloroplastic	-1.64	-1.46
RPL27	P30155	50S ribosomal protein L27, chloroplastic	-1.84	-2.48
RPL28	P30956	50S ribosomal protein L28, chloroplastic	-3.58	-2.94
RPL4	O80361	50S ribosomal protein L4, chloroplastic	-2.97	-2.09
RLA0	P50346	60S acidic ribosomal protein P0	-1.95	-2.17
RPLP0	P05388	60S acidic ribosomal protein P0	6.61	6.51
RLA2	P41099	60S acidic ribosomal protein P2	-2.53	-2.39
RPL10	Q9SPB3	60S ribosomal protein L10	-1.15	-3.20
Rpl10	Q6ZWV3	60S ribosomal protein L10	10.86	10.58
RPL10A	B8B9K6	60S ribosomal protein L10a	-2.23	-2.31
Rpl10a	P62907	60S ribosomal protein L10a	9.78	10.15
RPL11	Q3T087	60S ribosomal protein L11	9.91	10.23
RPL12	P30050	60S ribosomal protein L12	10.53	10.52
RPL13	P26373	60S ribosomal protein L13	10.26	10.41
RPL13B	P41127	60S ribosomal protein L13-1	-3.21	-1.91
RPL13A	P40429	60S ribosomal protein L13a	11.05	11.09
RPL13AD	Q9FKC0	60S ribosomal protein L13a-4	-2.56	-2.77
RPL15	P61313	60S ribosomal protein L15	9.76	9.92
RPL17	Q5XTY7	60S ribosomal protein L17	9.81	10.14
RPL17B	P51413	60S ribosomal protein L17-2	-1.82	-2.47
RPL18	Q07020	60S ribosomal protein L18	9.76	10.09
RPL18B	P42791	60S ribosomal protein L18-2	-2.32	-2.16
RPL18A	Q3T003	60S ribosomal protein L18a	9.67	10.10
RPL19	P84098	60S ribosomal protein L19	4.03	4.47
RPL19B	Q9LUQ6	60S ribosomal protein L19-2	-2.54	-1.91
RPL21	P46778	60S ribosomal protein L21	9.71	9.84
RPL23A	P49690	60S ribosomal protein L23	-2.89	-2.86
RPL23	P62829	60S ribosomal protein L23	9.99	10.32
Rpl23a	P62751	60S ribosomal protein L23a	9.99	10.26
RPL23A	O22644	60S ribosomal protein L23A	-2.65	-2.65
RPL24	Q862I1	60S ribosomal protein L24	9.28	9.78
RPL26	P61256	60S ribosomal protein L26	10.50	10.53
RPL27A	P46776	60S ribosomal protein L27a	10.39	10.25
RPL27AC	P49637	60S ribosomal protein L27a-3	-2.61	-2.36
RPL28	P46779	60S ribosomal protein L28	10.33	9.77

RPL28A	O82204	60S ribosomal protein L28-1	-2.08	-2.45
RPL29	P47914	60S ribosomal protein L29	5.16	5.29
RPL3	P39023	60S ribosomal protein L3	10.69	10.90
RPL30	O49884	60S ribosomal protein L30	-9.87	-6.61
RPL31	P62901	60S ribosomal protein L31	9.63	10.05
RPL32A	P49211	60S ribosomal protein L32-1	-3.00	-2.55
RPL35	P42766	60S ribosomal protein L35	9.61	9.38
RPL35AC	Q9C912	60S ribosomal protein L35a-3	-10.23	-6.88
RPL37A	Q5RBF9	60S ribosomal protein L37a	9.95	10.48
RPL4	P36578	60S ribosomal protein L4	6.66	6.59
RPL4A	Q9SF40	60S ribosomal protein L4-1	-2.43	-2.83
RPL5	P46777	60S ribosomal protein L5	9.78	9.12
RPL6	Q02878	60S ribosomal protein L6	10.18	9.76
RPL7	P18124	60S ribosomal protein L7	10.68	10.57
RPL7D	Q9LHP1	60S ribosomal protein L7-4	-2.29	-2.19
RPL7A	P62424	60S ribosomal protein L7a	11.23	11.11
RPL8	Q3T0S6	60S ribosomal protein L8	10.29	10.55
RPL9	P32969	60S ribosomal protein L9	10.20	10.30
AAG	Q9M6E9	Agglutinin-1	0.19	-1.62
POPTRDRAFT_821063	B9HQZ6	Alanine--tRNA ligase, chloroplastic/mitochondrial	-2.91	-1.82
APC11	Q9M9L0	Anaphase-promoting complex subunit 11	1.10	1.53
IBI1	Q9M084	Aspartate--tRNA ligase 2, cytoplasmic	-2.69	-2.09
CLPP3	Q9SXJ6	ATP-dependent Clp protease proteolytic subunit 3, chloroplastic	-2.31	-1.86
CLPP5	Q9S834	ATP-dependent Clp protease proteolytic subunit 5, chloroplastic	-2.16	-1.97
CLPR4	Q8LB10	ATP-dependent Clp protease proteolytic subunit-related protein 4, chloroplastic	-2.79	-3.10
BAG5	O65373	BAG family molecular chaperone regulator 5, mitochondrial	2.24	3.21
BT3	Q9SYL0	BTB/POZ and TAZ domain-containing protein 3	2.02	2.65
BT4	Q9FJX5	BTB/POZ and TAZ domain-containing protein 4	-1.12	-2.11
At1g55760	Q680K8	BTB/POZ domain-containing protein At1g55760	1.55	2.34
SR1IP1	Q66GP0	BTB/POZ domain-containing protein SR1IP1	-3.43	-2.08
CAL1	Q40401	Calreticulin	-2.86	-1.87
DJA6	Q9SJZ7	Chaperone protein dnaJ A6, chloroplastic	-3.03	-1.85
CPN60B1	P21240	Chaperonin 60 subunit beta 1, chloroplastic	-2.17	-2.30
CUL1	Q94AH6	Cullin-1	-1.99	-1.46
Dcaf8	Q8N7N5	DDB1- and CUL4-associated factor 8	1.28	2.05
RH3	Q8L7S8	DEAD-box ATP-dependent RNA helicase 3, chloroplastic	-2.25	-1.40
ATL76	Q6NML0	E3 ubiquitin-protein ligase ATL76	-2.79	-5.37
Neurl1b	Q0MW30	E3 ubiquitin-protein ligase NEURL1B	1.13	1.89
PUB23	Q84TG3	E3 ubiquitin-protein ligase PUB23	1.89	3.93
RHA2A	Q9ZT50	E3 ubiquitin-protein ligase RHA2A	1.08	2.25
RMA2	P93030	E3 ubiquitin-protein ligase RMA2	1.12	2.19
rnf12-b	Q7T037	E3 ubiquitin-protein ligase RNF12-B	-4.09	-4.22
RNF144A	P50876	E3 ubiquitin-protein ligase RNF144A	1.46	1.26
SGR9	Q8GXF8	E3 ubiquitin-protein ligase SGR9, amyloplastic	1.21	1.37
At5g37930	Q84K34	E3 ubiquitin-protein ligase SINA-like 10	5.09	4.32
At5g62800	Q9FM14	E3 ubiquitin-protein ligase SINA-like 11	-1.34	-3.41
SINAT2	Q9M2P4	E3 ubiquitin-protein ligase SINAT2	1.20	2.02
UPL3	Q6WWW4	E3 ubiquitin-protein ligase UPL3	-3.04	-2.22
UPL4	Q9LYZ7	E3 ubiquitin-protein ligase UPL4	1.14	1.96
WAV3	Q9LTA6	E3 ubiquitin-protein ligase WAV3	-1.14	-1.92
GCN2	P15442	eIF-2-alpha kinase GCN2	1.02	1.46
EF1A	P43643	Elongation factor 1-alpha	-2.13	-3.13
EF1A	P17786	Elongation factor 1-alpha	-5.68	-5.22
A1	P0DH99	Elongation factor 1-alpha 1	-2.42	-1.88
EEF1A1	Q66RN5	Elongation factor 1-alpha 1	7.55	7.58
At5g12110	Q84WM9	Elongation factor 1-beta 1	-2.67	-2.07
EEF1G	P26641	Elongation factor 1-gamma	10.25	10.66
eef-2	P29691	Elongation factor 2	-8.27	-11.00
EF2	O23755	Elongation factor 2	-2.44	-1.98
EEF2	Q5R8Z3	Elongation factor 2	10.34	10.50
fusA2	I1K0K6	Elongation factor G-2, chloroplastic	-2.39	-1.98
TUFA	Q40450	Elongation factor TuA, chloroplastic	-2.22	-2.37
HSPA5	P11021	Endoplasmic reticulum chaperone BiP	9.33	8.23

HSP90B1	Q4R520	Endoplasmin	8.23	8.02
HSP90	P35016	Endoplasmin homolog	-1.97	-2.24
EBP1	M1CZC0	ERBB-3 BINDING PROTEIN 1	-3.09	-2.81
IF4A9	Q40471	Eukaryotic initiation factor 4A-9	-2.82	-1.88
EIF4A1	P60842	Eukaryotic initiation factor 4A-I	9.32	9.42
Gspt1	Q8R050	Eukaryotic peptide chain release factor GTP-binding subunit ERF3A	-2.23	-2.14
ERF1-3	P35614	Eukaryotic peptide chain release factor subunit 1-3	-0.77	-0.03
N/A	P56331	Eukaryotic translation initiation factor 1A	-0.73	0.20
TIF3A1	Q40554	Eukaryotic translation initiation factor 3 subunit A	-2.03	-2.78
EIF4G	Q76E23	Eukaryotic translation initiation factor 4G	-2.65	-2.26
IF5A	Q9AXJ4	Eukaryotic translation initiation factor 5A	-2.99	-2.10
IF5A4	Q9AXQ3	Eukaryotic translation initiation factor 5A-4	-2.50	-3.75
EIF(ISO)4G1	Q93ZT6	Eukaryotic translation initiation factor isoform 4G-1	-9.16	-3.24
EDL2	Q9FLZ8	EID1-like F-box protein 2	1.48	2.15
AFR	Q8LAW2	F-box protein AFR	1.04	1.53
At2g27310	Q9XIN8	F-box protein At2g27310	1.43	1.41
At5g67140	Q9FH99	F-box protein At5g67140	1.30	2.45
CPR1	Q9SU30	F-box protein CPR1	1.13	1.79
PP2B15	O80494	F-box protein PP2-B15	-1.26	-2.37
SKIP23	Q3EBZ2	F-box protein SKIP23	1.14	1.93
SKIP11	Q8L736	F-box/kelch-repeat protein SKIP11	1.02	2.02
SKIP6	Q9SJ04	F-box/kelch-repeat protein SKIP6	-9.13	-9.13
At5g60570	Q9FKJ0	F-box/kelch-repeat protein At5g60570	1.08	1.78
At3g03360	Q84W80	F-box/LRR-repeat protein At3g03360	1.07	1.85
At1g08170	Q9SGE3	Histone H2B.2	2.67	4.04
At1g17710	Q9FZ62	Inorganic pyrophosphatase 2	1.11	1.80
ITM2B	Q5R876	Integral membrane protein 2B	9.54	9.87
BIP4	Q03684	Luminal-binding protein 4	2.38	3.42
NEAP2	F4K1B4	Nuclear envelope-associated protein 2	1.62	2.63
NUG2	Q9C923	Nuclear/nucleolar GTPase 2	-2.76	-2.56
NPM1	P06748	Nucleophosmin	9.32	9.15
PCKR1	Q39613	Peptidyl-prolyl cis-trans isomerase	-2.10	-2.04
CYP18-1	Q9LPC7	Peptidyl-prolyl cis-trans isomerase CYP18-1	1.80	2.71
CYP28	O65220	Peptidyl-prolyl cis-trans isomerase CYP28, chloroplastic	-2.90	-2.90
At4g39280	Q9T034	Phenylalanine--tRNA ligase alpha subunit, cytoplasmic	-2.85	-3.44
PAB8	Q9FXA2	Polyadenylate-binding protein 8	-1.98	-2.16
UBIQP	P42739	Polyubiquitin	-2.19	-3.07
BRG3	Q9LDD1	Probable BOI-related E3 ubiquitin-protein ligase 3	1.61	1.00
RHB1A	Q2HIJ8	Probable E3 ubiquitin-protein ligase RHB1A	1.54	2.89
XERICO	Q9SI09	Probable E3 ubiquitin-protein ligase XERICO	2.34	3.46
At1g77840	Q9S825	Probable eukaryotic translation initiation factor 5-2	-2.75	-2.86
ERD3	Q94II3	Probable methyltransferase PMT21	-3.08	-1.65
At2g34300	Q0WT31	Probable methyltransferase PMT25	-1.09	-3.75
At5g64030	Q8L7V3	Probable methyltransferase PMT26	-2.22	-2.36
MPPbeta	Q42290	Probable mitochondrial-processing peptidase subunit beta, mitochondrial	-3.84	-2.17
SEC	Q9M8Y0	Probable UDP-N-acetylglucosamine--peptide N-acetylglucosaminyltransferase SEC	-1.08	-0.27
AFB2	Q9LW29	Protein AUXIN SIGNALING F-BOX 2	-2.12	-2.31
P4HB	P07237	Protein disulfide-isomerase	9.79	9.52
PDIA3	P30101	Protein disulfide-isomerase A3	8.83	8.24
PDIA6	Q15084	Protein disulfide-isomerase A6	4.85	4.82
At5g01750	Q9LZX1	Protein LURP-one-related 15	2.54	3.45
SUI1	O82569	Protein translation factor SUI1 homolog	1.79	2.04
SUI1	O48650	Protein translation factor SUI1 homolog	-2.43	-2.44
DDB_G0268948	Q55EX9	Putative methyltransferase DDB_G0268948	2.13	2.72
FAM86B2	P0C5J1	Putative protein N-methyltransferase FAM86B2	1.13	1.53
APUM7	Q9C9R6	Putative pumilio homolog 7, chloroplastic	4.14	4.82
ATL21B	P0CH02	Putative RING-H2 finger protein ATL21B	5.63	6.96
R3HDM1	Q15032	R3H domain-containing protein 1	-2.34	-2.75
RNS2	P42814	Ribonuclease 2	1.86	3.20
ATL60	P0C035	RING-H2 finger protein ATL60	1.50	1.51
ATL66	Q9SRM0	RING-H2 finger protein ATL66	-1.17	-2.03
RPT2	Q682S0	Root phototropism protein 2	-1.94	-2.93
RUBB	P08927	RuBisCO large subunit-binding protein subunit beta, chloroplastic	-3.02	-1.91

FFC	P37107	Signal recognition particle 54 kDa protein, chloroplastic	-2.06	-1.71
SKP1A	Q39255	SKP1-like protein 1A	-1.98	-1.93
HSP70	Q02028	Stromal 70 kDa heat shock-related protein, chloroplastic	-1.57	-1.86
SBT1.5	Q9LUM3	Subtilisin-like protease SBT1.5	-1.51	-2.60
At2g36630	Q8S9J0	Sulfite exporter TauE/SafE family protein 4	-0.54	-0.14
CCT1	P28769	T-complex protein 1 subunit alpha	-3.11	-2.81
IF2CP	P57997	Translation initiation factor IF-2, chloroplastic	-2.01	-1.91
IF3-2	O82234	Translation initiation factor IF3-2, chloroplastic	-2.79	-2.13
tma7	Q4SUE2	Translation machinery-associated protein 7	-2.48	-2.52
At5g49980	Q9LTX2	Transport inhibitor response 1-like protein	-2.65	-2.16
Ufd1	P70362	Ubiquitin recognition factor in ER-associated degradation protein 1	1.12	1.96
UBI3	P62981	Ubiquitin-40S ribosomal protein S27a	-2.64	-2.01
RPS27A	P62992	Ubiquitin-40S ribosomal protein S27a	9.91	9.81
UBC14	P42747	Ubiquitin-conjugating enzyme E2 14	1.09	1.69
Os01g0962400	Q94DM8	Ubiquitin-fold modifier 1	1.33	2.67
PUB11	Q8GUG9	U-box domain-containing protein 11	-1.64	-2.72
PUB19	O80742	U-box domain-containing protein 19	4.54	5.18
PUB6	O48700	U-box domain-containing protein 6	1.16	1.22
VPE	P49045	Vacuolar-processing enzyme	2.31	3.49
Os01g0834700	Q0JHZ2	Zinc finger CCCH domain-containing protein 11	1.44	2.31

Table S17. Sequences of primer used in qRT-PCR validation.

Genes	Sequences (5' to 3')	Amplicon size (bp)
ACT	Forward: ACTATTGGTGCGGAGCGTTT<br>Reverse: CCGGGAACATTGTGGAACCT	178
**Drought stress (15)**		
ADH1	Forward: GCACCACCACAGAAAGATGAAG<br>Reverse: GCCTCATGACCAAAAATCCGAG	125
ADHIII	Forward: GTTTGTCTTCTGGGTTGCGG<br>Reverse: GCTTTAGCACCTTCTGCGAC	134
ANN1	Forward: CATCACACCACAGGGGACTT<br>Reverse: TTTGTGCCTTGCTCCTTGTG	187
CRY1	Forward: GGGTGTCAAGATGGTGGCTT<br>Reverse: ATCCCGCACGAGTGATATGG	188
CRY2	Forward: GCATTGGCTACCTGAGCTTG<br>Reverse: GGCGATCCCTTGCTACATCT	149
ERD14	Forward: CAGAAGAAGCCCGAGGATGT<br>Reverse: CCCCGGAAGTTTCTCCTTGAT	129
DRPD	Forward: CTTGACTGCGAAGTGTGGCT<br>Reverse: CCCAAAGAGCAGAACCGAAG	142
EDL3	Forward: TATGACATCGGCTCGGCTTG<br>Reverse: CCGTCATCCACTCCTTCGTT	162
HVA22E	Forward: GGCCCAGTTGTGATGTTGCT<br>Reverse: ACCAACCAAGCTGCCAAAATC	200
PLAT1	Forward: CGAGAGGGACACTTTGGATCT<br>Reverse: ACGTACCAACCGGGACTTTG	111
ARP1	Forward: GAGAGCTTGCGTTGATGCTG<br>Reverse: CCCCACCTGCTTGAAATCCT	188
ASPG1	Forward: TCGGTTGCAGAGTCAAGCAT<br>Reverse: GGAAAACTCAAACGCCACCG	157
ERD7	Forward: AGCTTTTTCCCGAGGACGAC<br>Reverse: CGTTTCCACCTTGGGAGAGG	168
REM4.2	Forward: GGCGGAAGTAGTGGAAGTGG<br>Reverse: TTGCATTTTGCCATGCCGAT	109
CDSP32	Forward: GTCATTCGGCTGTTGTGCAG<br>Reverse: AACACAGGGTCCACAATGCT	122
**Antioxidant enzyme activities (14)**		
BAS1	Forward: ATCCCATGTTCCCCGTTCTC<br>Reverse: GCCTCAAAGTCAGGTGCTTG	114
PER31	Forward: GCTTGAATGCCCCAATACCG<br>Reverse: GGAGACTTCCAACCACGGAG	152
PRXIIE-1	Forward: CTCCGCCACCATCTCTGTTG<br>Reverse: TTGTTAGCGGTGAGATCGGAG	108

PEX11C	Forward: AGATGTTGCCCGAACCGAAC<br>Reverse: TGCCAGGCTCTCCATTACTC	122
Gpx3	Forward: GTGGGGGCTTTGTGCCTAAT<br>Reverse: TTCCAGCGGATGTCATGGAT	172
Gpx4	Forward: CTAGTCGATCTGCATGCCCG<br>Reverse: GGCATCGTCCCCATTTACAC	174
APX1	Forward: CCACCAGTTGAAGGTCGTCT<br>Reverse: AGATGAGCGGGTTGGTAGTC	184
APX3	Forward: TGCGCTCCTATCATGCTTCG<br>Reverse: TGCGGAGTGAGAGTGTTCTTC	117
AFRR	Forward: CAGAACCATGGTGCATGCCT<br>Reverse: CATCCAGCACCCTACCATCC	174
MDAR4	Forward: TGGTTCTTTCCTTGAGGGTGG<br>Reverse: CTTCTCAAGTTCGCCCAGGT	100
CATA	Forward: TGACCGTGAACGAATCCCAG<br>Reverse: GGAGCACGAAGAAAATCCGC	117
CAT2	Forward: TGTCTCCCATCTCACTTGCG<br>Reverse: CGGGGATCACGGAGAGTTTC	120
PNC1	Forward: CTCCACTTCCACGACTGCTT<br>Reverse: AGATGTCTGCACACGAGACG	184
PNC2	Forward: GTGGAGTTGTCTCGTGTGCT<br>Reverse: GCCAACGATATGCGACCATC	118
**Soluble sugar and protein metabolism (33)**		
GLC1	Forward: CCGAGAGAAACTGGGGACTG<br>Reverse: GCATCGGTAGACCCTGCTTT	157
At2g16790	Forward: AACTTGGTGCTGGGATGTTCT<br>Reverse: GTTTTCCTTCCGCGGCTCTT	172
G6pc2	Forward: CAAGCCCATGTCTTGAGCAGT<br>Reverse: TACCATGACATACCAGACGCAC	101
GAPA	Forward: GCAACCCTGTCAACCTTCCT<br>Reverse: GGGGATGTCACCCTTTCCAG	152
PGM1	Forward: GGTATCTGGGCTGTTTTGGC<br>Reverse: GCCATCAATTCCTTCGCACC	185
Gcg	Forward: TTTACTTTGTGGCTGGATTGCT<br>Reverse: TGGGAAGCTGGGAATGATCTG	100
INVA	Forward: ACTGTCGGTGGACAAATGGC<br>Reverse: CAAGGATGCCGTTTCTGGGT	145
SUS2	Forward: TGGATGGTCAATTCCGGTGG<br>Reverse: AGGAAGCCCACAAGTCATGG	158
SPP1	Forward: ACAGTGGAGGGGTTGACTTG<br>Reverse: ATTAGGTGCTTTACCCTCGGC	104
Pfkl	Forward: TTGTGGAAGGAGGCGAGAAC<br>Reverse: CCGATGACACACACAGGTTGGT	178
FBP	Forward: AGCAAGAACAATCTGGGGCT<br>Reverse: AGAACACCTCGTTGGAGACG	189
ALFC	Forward: CTGAGTTCGTCAAGGGCCAG<br>Reverse: TGCAATTGTTTTCGCGGTCT	140
FBA6	Forward: TGAGCGTGTTCTAGCTGCAT<br>Reverse: CGGACGGTGTACTCAGCAAT	144
At4g10260	Forward: ATCCTAACGTTCGCTTGCCT<br>Reverse: TGCCACCTCATCATCACTCAC	110
BGAL	Forward: AATGGACACGAACCTGAGCC<br>Reverse: CACCAAAGTTCCATTCGGCG	142
GOLS1	Forward: CCAGCCAATTCCACCAGTGT<br>Reverse: TCTCGCTGCATGCTCTCTTC	153
OFUT7	Forward: ACATCTCCTTCAGAGCCACG<br>Reverse: GTGGCATTGATGATACGGGC	113
OFUT39	Forward: TGCAACGCTTGTAATCCCGT<br>Reverse: TCTGGTAGCTCGAATGGCTG	190
TPS6	Forward: CCATCCTTGAGTGGAGCCAT<br>Reverse: CCTGAAGAAAACTGCGTGCC	172
TPPA	Forward: ATGCTGGAAGTCATGGGATGG<br>Reverse: TCCTTGCCCTGCTCGTTAGT	100
MSR1	Forward: GACTAAGCCCGTGTTGGAGG	109

	Reverse: TACCACCACTGCATCAGCAAT	
MAN1	Forward: AGGGCTTGAAGGTTTCTATGGAG<br>Reverse: TGCTTACCAACTCTCTGGGT	142
DSP4	Forward: GATGGGTTTGATTTGCGGAGG<br>Reverse: AACATGTATGCCAGCGCAGT	140
PTST	Forward: CCTCTTACTTCCACACCGCC<br>Reverse: CGGTAGCCAAGAACTCCCTT	191
SS3	Forward: GCCTGCGGAAAATGGTTCTC<br>Reverse: GGGTTCCTTTGTGACCCCTC	163
APA1	Forward: ATTGCTCAGGAGGTTGTGCT<br>Reverse: TCCTTCTGCCCCAATTGCAT	104
FTSH5	Forward: GGGGTAAGCTGCCAAAAGGT<br>Reverse: GATCTCTCACCCTTCGTGCT	165
CYP-3	Forward: ATGCAGTCCTTGCTGTTGGT<br>Reverse: CACGGGGAATGATGCACAAG	155
LOC109345795	Forward: TTAGAGCCGATAGCCCCACT<br>Reverse: TGATGCGTCACCGGATTGAT	106
Os04g0650000	Forward: GCTGCTGTTGGTTATGGAACT<br>Reverse: GTTTCAGGCAAATTACGCTCC	116
RMD5	Forward: GTGCCGGTTGAATTGGGAAG<br>Reverse: GCCTCGGTAGGGCAATAAGG	191
SCPL34	Forward: CTGATGATTGCGAACGAGCC<br>Reverse: GGGCAGTCGTCTAACTCCAG	122
At4g32940	Forward: TTTTTGAGGGCCTCTTGCCT<br>Reverse: AGTCACCCAAGCAAGTCTCG	138
**Cell morphogenesis (12)**		
GMPM1	Forward: AGCAATCTGCCAAAGTCGGT<br>Reverse: GATGCCGTTCCTGTTCCTGT	112
ASP	Forward: TTTGACGGGTGCTCCAGTAG<br>Reverse: CCTTCAACTCCATCGGTCGT	186
ACT7	Forward: GTTGCACCACCAGAGAGAAAGT<br>Reverse: GGCCAGACTCTTCATACTCAGC	112
AC97	Forward: CGAGCTCAGCAGTCGAGAAG<br>Reverse: AAGTGGTCTCGTGAATGCCT	141
AP2	Forward: CCCCTAAAGAAGAGTCGCCG<br>Reverse: GCCGTGTCAAAACCACCAAG	140
At4g25140	Forward: TGACGACGGGGTTCTTGTTC<br>Reverse: TGTCCGTACTCCCTAGCCTT	157
At5g40420	Forward: TCGCCACCCTTTTACCAGTC<br>Reverse: AGCCAAGCCGAAGACAAGAG	149
OLE18	Forward: CTCATCGTTTCCAGCCCCAT<br>Reverse: CGTAATCCACCCTGTCCGAT	169
SPD1	Forward: GGTCGAGCTGTCTCTGGAAG<br>Reverse: CATCTGCCAACATTCTCGCC	133
SBP65	Forward: TTCGCTTGCGGAAATTGGTC<br>Reverse: TGCGAGGGTCTTTCAGTTCC	189
pec2a1a	Forward: TGGATGTCTCCGTCGCTTTC<br>Reverse: ATCCCATTTCAAACCGCCCT	120
At2g18540	Forward: TTTGTCCGAGCAATGGCAAC<br>Reverse: TCACTGTTGTGCTGAACCCC	153
**Transcription factor (12)**		
MYB4	Forward: ACCACCACCAGAACCCAATC<br>Reverse: ATGAACACCAGCACCGTTTG	168
MYB102	Forward: AGTTCCTCTCCCATCCGAAAC<br>Reverse: TTTCCCTCTCGTCTTCAGTGC	188
MYB330	Forward: AAACTCACCGCCCTCTCAAC<br>Reverse: CGGATTTTGCGTTTGCATCG	182
BZIP34	Forward: CGATTGCCGTGGAACTGTTG<br>Reverse: TTCAAACTCGTGGTGGTCGT	112
BZIP53	Forward: TTAGTTGACGAGCGGAAGCG<br>Reverse: TCCTAAGCCGGTTCACTTGG	118
WRKY6	Forward: TCAACGGCTGAGAGAGATGC<br>Reverse: TCGTCTGCTTCTTGCTCGTT	135
WRKY71	Forward: GTGGAGAGGTCCTACCAGGAT	138

	Reverse: CATCGGCGAGTGGGTTAGAG	
NAC019	Forward: GAAGTATCCAAACGGGTCGC<br>Reverse: GGTTCCTTTGGGGGCCTTAC	151
NAC92	Forward: AAAATTACGGCCAAGGATCGC<br>Reverse: TAATCTTGGACCCCTTACCCCT	139
NAC056	Forward: CACCACTCCCCGTTGCTATT<br>Reverse: AAGTACCATTCCTGCTCGCC	100
BHLH94	Forward: ACCAGGCCTCCATAGTTGGT<br>Reverse: TGCTTCTTCGTTGCCTTGTTG	113
BHLH128	Forward: ACTCTCAGTTTAGCCTGCCG<br>Reverse: TCCCTTTCGGCAATGCTTCT	142
**Hormone response (18)**		
GID1B	Forward: CCGCATTTACCAACCTGCAC<br>Reverse: AATGGCACTGTTTGCCGATG	145
ABP20	Forward: AACATCATCAAGGCGGCAGT<br>Reverse: GTTCTGATGCTCCAGGGTGT	139
AX10A	Forward: TTTCGCGGTTTATGTTGGGG<br>Reverse: AGGGAATTGTAAGCCCTCCC	146
ARF5	Forward: CTGAGAGCTGGCGATTCTGT<br>Reverse: AATGGTGAATGGGCTTCGGT	183
IAA16	Forward: GAGATTAGGACTGCCTGGAGC<br>Reverse: CAGCTTCACCAACACAGGAAC	131
SAUR32	Forward: GCAACTCAATGCGGAAAACC<br>Reverse: TCTCTGCAATTCGTCTGGTCG	156
AHK2	Forward: AGTGGCATGCTGGAGGAAAC<br>Reverse: TCAGCCTGCACAAAAGGTGT	116
AHP4	Forward: TGATGCTAACCCCCATTTCG<br>Reverse: CTAGCTTGCCGAAGTCGATG	119
ARR3	Forward: AGCGGTTGCTCACGATTTCT<br>Reverse: TCAAGGCCATTCACGTTCTG	106
CYP94B1	Forward: CGGCGGAATCTAAGGTGGTT<br>Reverse: GGCGAAACCGTTGAAGAAGG	121
GRXC9	Forward: TTCGCCAGAGAATCGACCG<br>Reverse: AAAAGCAGACGCTTCACCAC	107
CPN20	Forward: GGGGGCATCCTATTGCCTAC<br>Reverse: ACAATCCGGGTTCCAGTCTTC	128
PYL4	Forward: GTGTGGTCCTTAGTCCGTCG<br>Reverse: ATCTCAAGCCTCTCCGTGCT	158
NRP1	Forward: CGGCGAATCTTCTTGATGCG<br>Reverse: TGAGAAGAGTGGCTGTCGTG	124
ERF.C.3	Forward: TTTTCGATGCGTGGGTCACT<br>Reverse: TAAGGCCACAACAGGAGAGC	114
ERF1B	Forward: AAGTTCGCGGCGGAGATAAG<br>Reverse: TTTCCCGGAGCGATTCTTCT	175
ERF113	Forward: AAAGGCAGCCCGAGTATGG<br>Reverse: GAACTACTACCACTGCCGCT	174
EIN3	Forward: ATGCAGCACTGTGATCCTCC<br>Reverse: CTTGTATGGCGGTGGACCTT	135
